# Ancient RNA from Late Pleistocene permafrost and historical canids shows tissue-specific transcriptome survival

**DOI:** 10.1371/journal.pbio.3000166

**Published:** 2019-07-30

**Authors:** Oliver Smith, Glenn Dunshea, Mikkel-Holger S. Sinding, Sergey Fedorov, Mietje Germonpre, Hervé Bocherens, M. T. P. Gilbert

**Affiliations:** 1 Section for Evogenomics, The Globe Institute, Faculty of Health and Medical Sciences, University of Copenhagen, Copenhagen, Denmark; 2 Greenland Institute of Natural Resources, Nuuk, Greenland; 3 Mammoth Museum, Institute of Applied Ecology of the North of the North-Eastern Federal University, Yakutsk, Russia; 4 Directorate Earth and History of Life, Royal Belgian Institute of Natural Science, Brussels, Belgium; 5 Department of Geosciences, Palaeobiology, University of Tübingen, Tübingen, Germany; 6 Senckenberg Centre for Human Evolution and Palaeoenvironment, University of Tübingen, Tübingen, Germany; 7 Norwegian University of Science and Technology, University Museum, Trondheim, Norway; Wellcome Sanger Institute, UNITED KINGDOM

## Abstract

While sequencing ancient DNA (aDNA) from archaeological material is now commonplace, very few attempts to sequence ancient transcriptomes have been made, even from typically stable deposition environments such as permafrost. This is presumably due to assumptions that RNA completely degrades relatively quickly, particularly when dealing with autolytic, nuclease-rich mammalian tissues. However, given the recent successes in sequencing ancient RNA (aRNA) from various sources including plants and animals, we suspect that these assumptions may be incorrect or exaggerated. To challenge the underlying dogma, we generated shotgun RNA data from sources that might normally be dismissed for such study. Here, we present aRNA data generated from two historical wolf skins, and permafrost-preserved liver tissue of a 14,300-year-old Pleistocene canid. Not only is the latter the oldest RNA ever to be sequenced, but it also shows evidence of biologically relevant tissue specificity and close similarity to equivalent data derived from modern-day control tissue. Other hallmarks of RNA sequencing (RNA-seq) data such as exon-exon junction presence and high endogenous ribosomal RNA (rRNA) content confirms our data’s authenticity. By performing independent technical library replicates using two high-throughput sequencing platforms, we show not only that aRNA can survive for extended periods in mammalian tissues but also that it has potential for tissue identification. aRNA also has possible further potential, such as identifying in vivo genome activity and adaptation, when sequenced using this technology.

## Abstract 2

Ancient DNA is known to survive in cold environments for tens of millennia, but it is assumed that ancient RNA could not persist in such a way due to its relative instability. However, this study shows that under permafrost conditions, ancient RNA can survive well enough to show tissue specificity even in mammalian soft tissues.

## Introduction

The recent revolution in the sequencing of ancient biomolecules has allowed multiple layers of -omic information—including genomic [1], epigenomic [2,3], metagenomic [4,5], and proteomic [6,7]—to be gleaned from ancient and archaeological material. This wealth of evolutionary information almost all derives from either DNA or protein, biomolecules both traditionally thought to be considerably more stable than RNA. This is unfortunate, because transcriptome data have the potential to access deeper layers of information than genome sequencing alone. Most notably, these include assessments of the in vivo activity of the genome and assessing other aspects of ancient bio-assemblages, such as biotic colonisation/microbiomes [8], host–pathogen interactions [9], and the level of postmortem molecular movement within remains and surrounding media [10].

Despite the dominance of DNA, in recent years several studies have begun to explore whether or not RNA survives in archaeological substrates, particularly in the context of plant materials. Next-generation sequencing (NGS) approaches have uncovered viral RNA genomes in barley grains and faecal matter [11,12], environmentally induced differential regulation patterns of microRNA and RNA-induced genome modifications in barley grain [13,14], and general transcriptomics in maize kernels [15]. All but one of these datasets, however, have been derived from plant seed endosperm, which often facilitates exceptional preservation [16,17] and is known to be predisposed to nucleic acid compartmentalisation [18], thus allowing for reasonable expectations of such preservation. The conjecture that ribonucleases released during soft tissue autolysis would virtually annihilate RNA had, until recently, discouraged researchers from attempting such sequencing in animal tissues in favour of more stable molecules. This is exemplified by the fact that to date, ancient RNA (aRNA) data have been generated directly from ancient animal (human) soft tissues in only one example [19], and this was without utilising NGS technology. Instead, a targeted quantitative PCR (qPCR) approach was used, presumably intended to bypass extraneous noise that might be expected in ancient NGS datasets. The recent qPCR-based approach to microRNA identification demonstrated persisting specificity in permafrost-preserved human tissues [19] and thus opened the possibility of a more complete reconstruction of ancient transcripts in soft tissues when preserved under favourable conditions. While complexities surrounding the survival of purified RNA within a long-term laboratory storage setting are well documented [20,21], the complex thermodynamics of RNA lability and enzymatic interactions are themselves not well understood, especially within long-term postmortem diagenesis scenarios [22]. There is evidence suggesting that the survival of purified (modern) RNA is influenced by the specific tissue from where it originated [23], suggesting co-extraction of tissue-specific RNases is a significant problem. Others have suggested that the chemical structure of RNA is such that its theoretical propensity for spontaneous depurination is less than that of DNA [24]. Although strand breakage should occur more often, the observable depletion of purified RNA within a laboratory setting could be attributable to contamination from RNases that, speculatively, may be active in purified samples even when frozen. Because chemical and enzymatic interactions in archaeological or paleontological assemblages are generally unpredictable at the molecular level, it is possible that the activity of RNAses, and the susceptibility of RNA to those enzymes within a complex matrix of biomatter, could be slowed or arrested through uncharacterised chemical interactions. As such, it is possible that under environmental conditions such as desiccation or permafrost, aRNA may indeed persist over millennia.

Exceptionally well-preserved remains provide an opportunity to test this hypothesis. Given this, we decided to take advantage of some recently recovered samples exhibiting a range of ages and DNA preservation [25]. These 5 samples represent tissues from 3 individuals: skin from two historical wolves from Greenland (19th and 20th centuries CE), and liver, cartilage, and muscle tissue from a Pleistocene (approximately 14,000 years old) ‘wolf’ puppy from Tumat, Siberia (Table 1). We use the term ‘wolf’ in inverted commas as the domestication status of this individual is yet to be fully ascertained. Because the DNA of these samples was sequenced on both Illumina and BGISEQ, we felt these were ideal animal candidates to test for the persistence of aRNA in such contexts. The results presented here describe the oldest directly sequenced RNA, by a significant margin of at least 13,000 years, alongside younger tissues that still may be seen as novel substrates, given the prevailing RNA dogma. For context, the oldest RNA so far to have been recovered and verified without direct sequencing is approximately 5,000 years old [19], and the oldest RNA to be sequenced and verified is just over 700 years old [15].

**Table 1:** Basic sample details including age, tissue, and RNA extraction statistics.

Sample	Museum accession	Species	Tissue	Age	Location	Mass tissue (mg)	RNA (ng/uL)	Total (100 uL)	RNA from tissue (ug/g)
Skin 1	CN 1921	Wolf	Skin	Before 1869 CE	Uummannaq, Greenland	47.9	3.1	310	6.47
Skin 2	CN 214	Wolf	Skin	1925 CE	Rosenvinge Bugt, Greenland	134.7	4.61	461	3.42
Tumat cartilage	FRC	Canid	Cartilage	ca. 14122 YBP	Tumat, Siberia	665.3	3.19	319	0.48
Tumat liver	L	Canid	Liver	ca. 14122 YBP	Tumat, Siberia	612.9	3.54	354	0.58
Tumat muscle	M1	Canid	Muscle	ca. 14122 YBP	Tumat, Siberia	351.9	1	100	0.28
Blank	B	BLANK	n/a	n/a	n/a	0	0	0	0.00

Abbreviations: CE, common era; ca., circa; n/a, not applicable; YBP, years before present.

To verify with authenticity of the RNA described in this study, we performed several analyses including exon/exon boundary mapping, metagenomic profiling, damage pattern analysis, and a method to assign most likely tissue of origin when compared with a control dataset. To confirm the absence of platform-specific biases between sequencing platforms such as optical duplication, size biases, and preference for sequencing GC-rich reads, we sequenced each sample using the Illumina HiSeq-2500 platform and performed a technical replicate (library and sequencing) on the BGISEQ-500 platform. For clarity, the biological results and interpretations shown in the main text refer to HiSeq-2500 data because Illumina sequencing platforms are the most often used for sequencing ancient DNA (aDNA), with BGISEQ-500 comparisons referenced directly where necessary. A more detailed evaluation of the two sequencing platforms can be found in S1 Text.

Our analysis pipeline was a multifaceted approach, paying particular attention to read duplication (where multiple identical reads are sequenced, often a result of using more PCR cycles than would normally be required for library amplification of modern, fresh DNA/RNA). The need to de-duplicate datasets, in which identical reads are collapsed to a single read, is much debated amongst RNA researchers because of uncertainty about whether duplicates represent biological expression or an artefact of the PCR process [26]. Considering the short nature of our RNA reads and the generally high duplication rate, we surmised that these were more likely to be a PCR artefact than a reflection of biological expression. We performed all analyses using both the unmodified (‘duplicated’) and de-duplicated sets and found that in all cases, de-duplicated data made more biological sense. For our analyses, we compared the ancient samples against control NGS data of equivalent dog tissue, downloaded from the European Bioinformatics Institute (EBI)’s short read archive. These data represent transcriptomes for each tissue (liver, muscle, cartilage, skin) from healthy modern dogs, as part of the Broad Institute’s Canine Genomic Resources, and as such can be considered the ‘type’ tissue for canine transcriptomic work. To overcome potential biases arising from the uniquely short nature of the aRNA reads, we opted to test these data against control references using two distinct methods. One was a direct read-for-read regression using a statistical method often used for RNA comparisons (Varistran, ‘Method 1’) [27]. The second was based on coverage depth calculation of individual genes, followed by a cumulative scoring system based on the most-likely tissue assignation of those genes, derived from a multi-tissue Affymetrix expression array [28] (‘Method 2’). Finally, to assess the effect of ultrashort (between 15 and 30 nucleotides [nt]) fragments, we created additional control datasets from our existing primary and control sequencing data. First, we removed all fragments shorter than 30 nt from the aRNA, and second created a ‘simulated’ aRNA dataset from the modern control data. We then repeated our analyses on these new data and found that in general, retention of ultrashort molecules is appropriate for transcriptome mapping.

From the results presented here, we propose that the range of aRNA sources could now extend to permafrost samples of the Pleistocene age, thus opening up the possibility of using aRNA as a valuable biomolecular resource for future research. We should, however, emphasise that the samples presented here are exceptionally well preserved and exhibit varying degrees of preservation within a single individual, and so we are not suggesting that aRNA sequencing could now become as routine as aDNA work. We suggest that aDNA preservation could provide a tenuous proxy for potential aRNA preservation, although we would advise researchers to make no assumptions of relative preservation when considering attempting aRNA work, and consider the risks and benefits of further destructive analysis at these early stages of aRNA research.

## Results

### RNA recovery and sequence data from ancient tissues

From between 47 mg and 665 mg of tissues, including skin, cartilage, liver, and skeletal muscle, we recovered between 100 ng and 461 ng RNA (Table 1). Unsurprisingly, there was a marked difference between the ancient Tumat and historical samples: while the historical skin samples gave between 3.4 μg and 6.7 μg RNA per gram tissue, the ancient Tumat samples only gave between 0.28 μg and 0.57 μg per gram. After sequencing and mapping, we calculated the endogenous RNA content of the tissues to be between 7.4% and 80.0% using the HiSeq-2500 platform (Table 2).

**Table 2:** NGS data and mapping summary, with calculations of endogenous content and RNA enrichment factors.

Sequencing platform	Sample number	Museum accession	Species	Tissue	Age	Total reads post- adapter trimming	Genome	mRNA	rRNA	Proportion rRNA	tRNA	RNA enrichment factor	Endogenous percent
BGISEQ	Skin 1	CN 1921	Wolf	Skin	Before 1869 CE	69,053,233	26,043,866	6,858,947	16,714,271	31.03%	4,243,690	14.69	37.72%
	Skin 2	CN 214	Wolf	Skin	1925 CE	6,675,338	5,581,322	1,288,462	4,696,537	39.40%	354,381	15.62	83.61%
	Tumat C	FRC	Canid	Cartilage	ca. 14122 YBP	44,765,013	2,244,289	783,522	401,982	11.61%	32,077	7.46	5.01%
	Tumat L	L	Canid	Liver	ca. 14122 YBP	27,626,403	16,509,691	5,038,336	3,570,007	10.91%	7,617,698	13.52	59.76%
	Tumat M	M1	Canid	Muscle	ca. 14122 YBP	66,780,343	3,815,483	1,057,959	1,357,348	20.73%	317,792	9.85	5.71%
	Blank	B	BLANK	n/a	n/a	1,701,272	56,822	20,808	126,467	55.43%	24,069	41.47	3.34%
HiSeq	Skin 1	CN 1921	Wolf	Skin	Before 1869 CE	23,258,645	11,366,481	3,493,902	7,612,932	31.83%	1,441,633	15.18	48.87%
	Skin 2	CN 214	Wolf	Skin	1925 CD	32,927,602	26,320,301	5,618,346	19,883,788	36.95%	1,990,974	14.36	79.93%
	Tumat C	FRC	Canid	Cartilage	ca. 14122 YBP	20,915,948	2,354,199	1,064,732	209,067	5.71%	31,676	7.63	11.26%
	Tumat L	L	Canid	Liver	ca. 14122 YBP	6,811,527	4,114,476	1,882,220	1,192,800	14.94%	796,571	12.94	60.40%
	Tumat M	M1	Canid	Muscle	ca. 14122 YBP	39,878,232	2,932,798	1,099,000	818,537	16.44%	127,563	9.59	7.35%
	Blank	B	BLANK	n/a	n/a	1,339,288	75,612	91,929	9,498	5.33%	1,029	18.63	5.65%

Abbreviations: CE, common era; ca., circa; n/a, not applicable; NGS, next-generation sequencing; rRNA, ribosomal RNA; tRNA, transfer RNA.

### RNA enrichment

For each sample, we took the number of reads mapping to the entire genome and, similarly, the number of reads mapping to only the transcribed regions of the genome (mRNA, rRNA, and tRNA). We then divided the RNA read frequency with the whole-genome read frequency for each sample to give an enrichment factor (Table 2). We found between 7.4-fold and 15.6-fold enrichment for transcripts from HiSeq-2500 data. We found no significant age- or tissue-related correlation to enrichment level.

We subjected earlier DNA sequencing data from the same samples used in this paper [25] to the same transcriptome mapping pipeline as our RNA data in order to confirm that the enrichment of transcriptomic reads we saw in the RNA data was not spurious or the result of DNA contamination. As with the RNA data, we calculated the RNA enrichment factor for each sample. Whereas we saw at least 7.4-fold transcript enrichment for the RNA data, we saw only between 0.2- and 1.2-fold enrichment for the equivalent DNA data. Furthermore, while the RNA data showed that a large proportion of the endogenous content for each sample (between 5.7% and 37%) was of ribosomal origin, the ribosomal content of the endogenous DNA was significantly lower, between 0.09% and 0.15%, and we suspect more likely a representation of rRNA genes than their transcripts. Considering this, and the known high abundance of rRNA as a proportion of cellular RNA, this strongly suggests that the RNA sequencing (RNA-seq) dataset represents authentic RNA, with minimal, if any, DNA contamination.

To ensure that the mapping strategy using bowtie2 was not prone to biases, we repeated transcriptome mapping using bwa-aln v7.17 [29], using standard aDNA parameters (seed size disabled, allow indels). We found proportionally equal numbers of reads mapping to each sample but only around half as many overall when using bwa-aln (S1 Table). MapDamage analyses of the resulting BAM files gave similar results to those produced with bowtie2 (S1 Fig), suggesting that the greater stringency seen with bwa-aln does not result in any more authentic mapping.

### GC content and read duplication

The GC content of full reference transcripts falling within the 95th percentile of abundance was between 51% and 57% (S2 Table). We noted that the GC content of reads mapping to those transcript sets exhibited higher GC content than the transcripts themselves, which is not unexpected considering previous aRNA results [13,15,19]. On average, the de-duplicated datasets had 4.6% greater GC content than the references, and the unmodified (duplicated) datasets showed on average 7.3% higher GC content. This suggests a slight bias towards high-GC fragments being preserved, which is, again, not unexpected in RNA-seq data, given that transcribed regions of the genome are generally GC rich [30]. However, the uniquely short nature of read fragments, compared with a modern RNA dataset, combined with nonuniform GC content across a given transcript, suggests that the GC bias observed here does not skew the resulting transcription profiles. Due to the high number of PCR cycles (20) required to build libraries, it is unsurprising that we observed significant duplicate reads in all ancient samples, between 80.9% and 87.1%. However, at least some of this variance can be explained by ‘true’ transcript abundance, exemplified by the control data from modern material being between 20.9% and 39.4% duplicate reads. Further discussion of read duplication in RNA datasets can be found in S1 Text.

### Junction analysis

To further establish that we had sequenced RNA as opposed to contaminant single-stranded DNA (ssDNA), we assessed the frequencies of reads straddling intron-exon (splice) junctions and those straddling exon-exon junctions. Only reads crossing these boundaries were included in our analysis, as opposed to those that were merely proximal to the junctions. With RNA-seq data, we would expect to observe a high proportion of exon-exon reads to demonstrate that precursor mRNA processing has taken place in active transcripts, but we would also expect to see a degree of intron/exon reads representing precursor mRNA themselves. We found that in all cases, the number of reads mapping to exon/exon junctions was greater, often by orders of magnitude, than those mapping to splice junctions (S3 Table). In particular, the Skin 2 and Tumat liver samples respectively showed 186-fold and 68.5-fold more reads mapping to exon-exon junctions than splice junctions. We then repeated this analysis using DNA data generated from the same samples, as a negative control [25]. We found the DNA data showed the opposite trend to RNA-seq data, with exon-exon junctions being significantly underrepresented compared with splice junctions in all cases. These analyses further suggest that our primary data represent authentic aRNA.

### Damage profiles

Damage profiles were not consistent with typical aDNA profiles, although the expectations for comparing RNA and DNA in this manner are unknown due to a general lack of aRNA NGS data. mapDamage analysis of earlier DNA sequencing of the same samples showed profiles that were typical of aDNA, although at low levels for samples as old as the Tumat canid. Unsurprisingly, the two samples with the lowest levels of damage were the historical skin tissues. Interestingly, the liver sample, which showed the greatest similarity to its modern counterpart in transcriptome analysis, had the lowest damage levels of all tissues from the Tumat canid, further suggesting its exceptional preservation.

The RNA profiles themselves showed either low levels of damage throughout when de-duplicated, and some elevated C > U transitions towards the centre of the molecule (S2 Fig, S3 Fig). Interestingly, the damage appears at lower levels than the equivalent DNA samples. The damage was generally limited to C > U misincorporations as opposed to G > A misincorporations, which is consistent with data deriving from a single-stranded library construct. Damage patterns were more pronounced when duplicates were retained, which is unsurprising considering the high level of sequence duplication. We also note that the damage in general is more pronounced in data from the HiSeq-2500 platform. Following removal of ultrashort fragments, we found a more ‘typical’ damage profile, with discernible C > T damage at both 5′ and 3′ ends of reads (S4 Fig, S5 Fig) and a more typical excess purine pattern at fragment sites. As with the majority of aRNA damage analyses presented here, these observations are not necessarily unexpected; however, more data are needed from other sample types for palaeogenomics researchers to create these expectations.

### Statistical inter- and intra-tissue comparisons of ancient transcriptomes (Method 1)

Over the entire dataset, ordination and clustering revealed that the ancient samples were globally more similar to each other than to the control samples and vice versa. However, when considering individual ancient/historical samples against all control samples, we found that the ancient Tumat liver and historical Skin 2 samples were most similar to their modern counterparts. Clustering also revealed a set of 71 genes with relatively highly abundant transcripts across all, or most, ancient samples in comparison with the control samples (S6 Fig, S2 Table).

**Fig 1:**
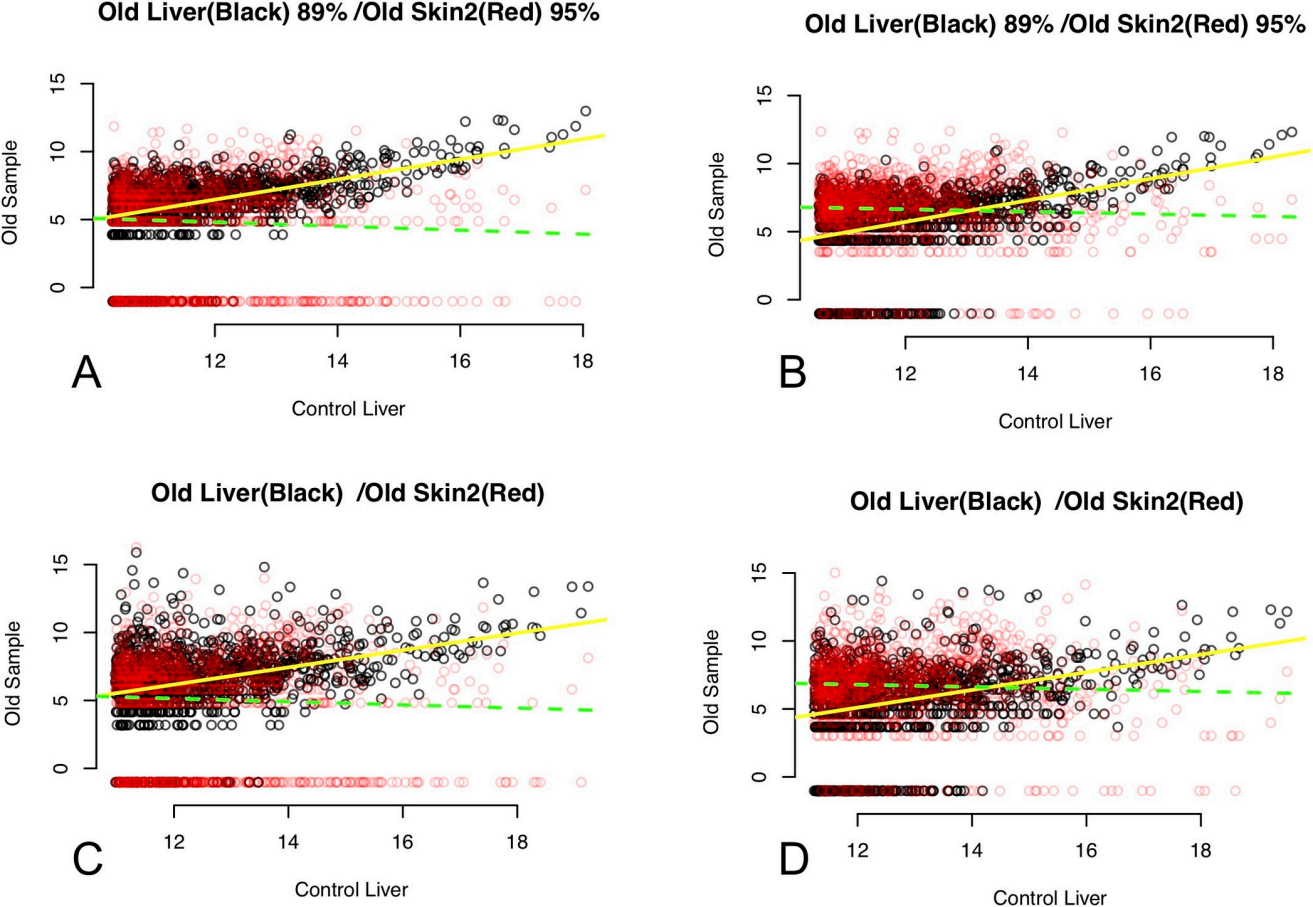
Regressions of ancient liver and historical skin samples, Method 1: Relationships between 95th percentile of expressed genes in each control tissue sample (x-axis) and each ancient sample or control samples from other tissues (y-axis). Black points in graphs comparing ancient samples are the relationships between the control tissue and the equivalent ancient tissue. Red points overlaid show the relationship between the control tissue and other ancient tissues specified in the graph subtitle. Yellow lines are least squares linear regression fit for black points. Green lines are least squares linear regression fit for red points. Filled lines indicate a significant linear regression. Dashed lines indicate a nonsignificant linear regression. (A) BGISEQ-500 data, de-duplicated; (B) HiSeq-2500 data, de-duplicated; (C) BGISEQ-500 data, duplicates retained; (D) HiSeq-2500 data, duplicates retained. The underlying data for this figure are derived from Varistran output, summaries of which can be found in S2 Data and S3 Data.

Considering the most highly expressed genes (i.e., 95th percentile) in each control tissue, there were some relationships of note between control and ancient samples. There was a significant relationship between control liver and ancient liver, with control liver expression explaining 16% (adjusted *R*^2^ values) of the variation in ancient liver transcript abundance (S2 Data; Fig 1). Control liver gene expression was more similar to ancient liver transcript abundance in comparison with any of the other ancient samples or any of the other control samples (S1 Data). Similarly, there was a significant relationship between control skin gene expression and transcript abundance in the historical Skin 2 sample, with control skin expression explaining 8% of the variation in historical Skin 2 transcript abundance (S1 Data; S7 Fig, S8 Fig, S9 Fig, S10 Fig). There was also a marginally significant relationship between control skin and historical Skin 1 (*P* = 0.012, α = 0.01); however, it explained only a very small amount of the variation in Skin 1 transcript abundance (0.4%; S1 Data). Control skin gene expression was more similar to both historical skin sample transcript abundance(s) in comparison with any of the other ancient samples; however, there were also significant relationships with all other control tissues (S1 Data). There was no relationship between control cartilage gene expression and ancient cartilage transcript abundance, although there was a relationship with Skin 2 transcript abundance, control liver, and control skin gene expression (S1 Data). There were no significant relationships between control muscle gene expression and any of the ancient samples or the other control samples. All pairwise regression parameters and details are provided in S2 Data and S3 Data.

### Tissue specificity when compared with the Canine Normal Tissue Database (Method 2)

**Fig 2:**
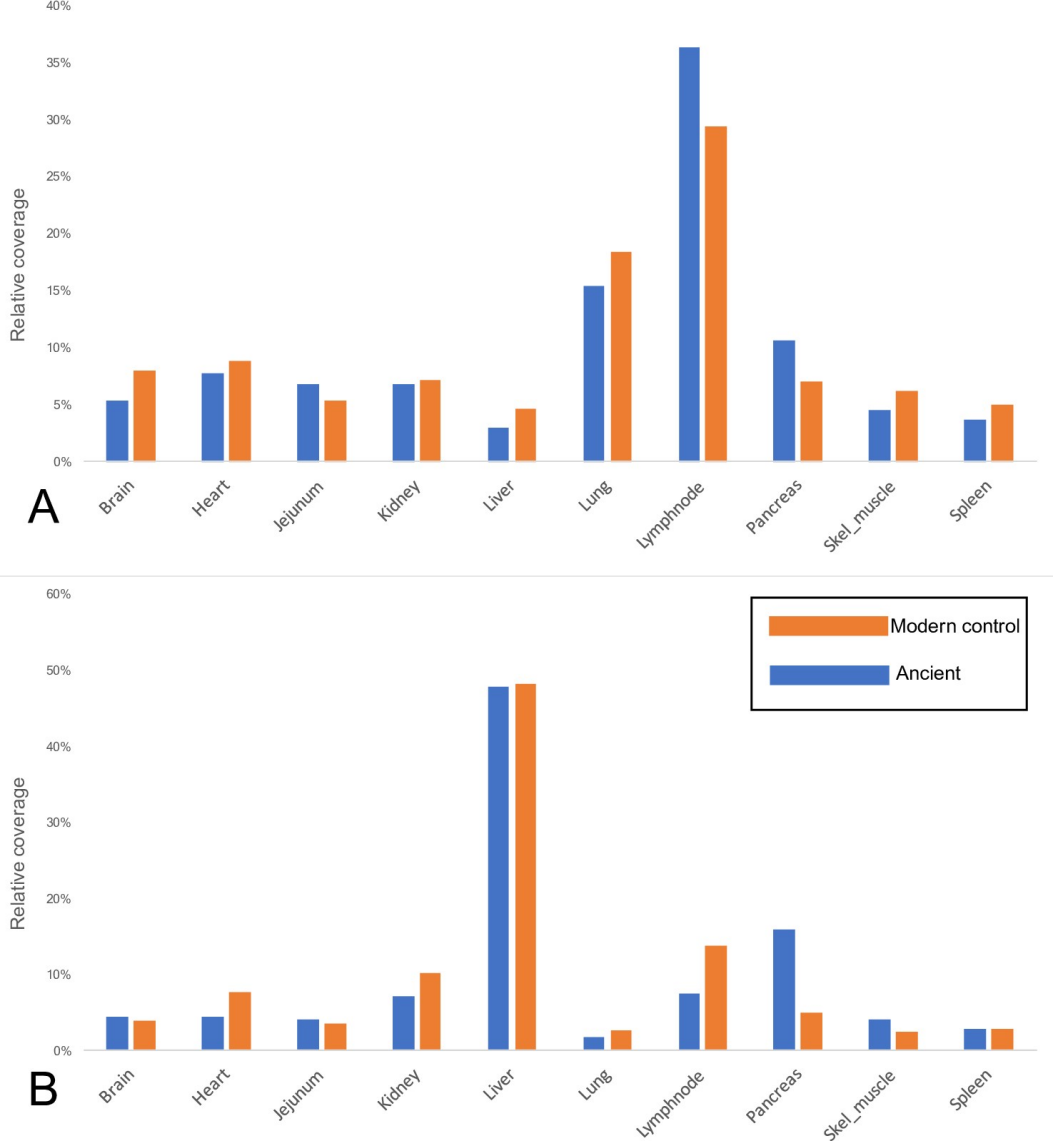
Comparison of ancient and control tissues using Method 2. Coverage scores (y-axis) were calculated based on the mean coverage of reads to each named gene in the CanFam3.1 transcriptome, followed by filtering to the 95th percentile of all genes represented. Each gene was then assigned a most-associated tissue based on data from an Affymetrix array derived from 10 canine tissues (x-axis). Each tissue was then assigned a cumulative score based on the coverage scores of each gene in the 95th percentile. Orange bars represent modern control tissues and blue bars represent ancient/historical tissues. (A) Historical Skin 2 versus control skin. (B) Ancient Tumat liver versus control liver. The underlying data for this figure can be found in S7 Data under Tissue_summary tabs, and are derived from primary data found in S4 Data under the Skin2_HS and Tumat_liver_HS tabs and from S5 Data under the ctrl_skin and crtl_liver tabs.

**Fig 3:**
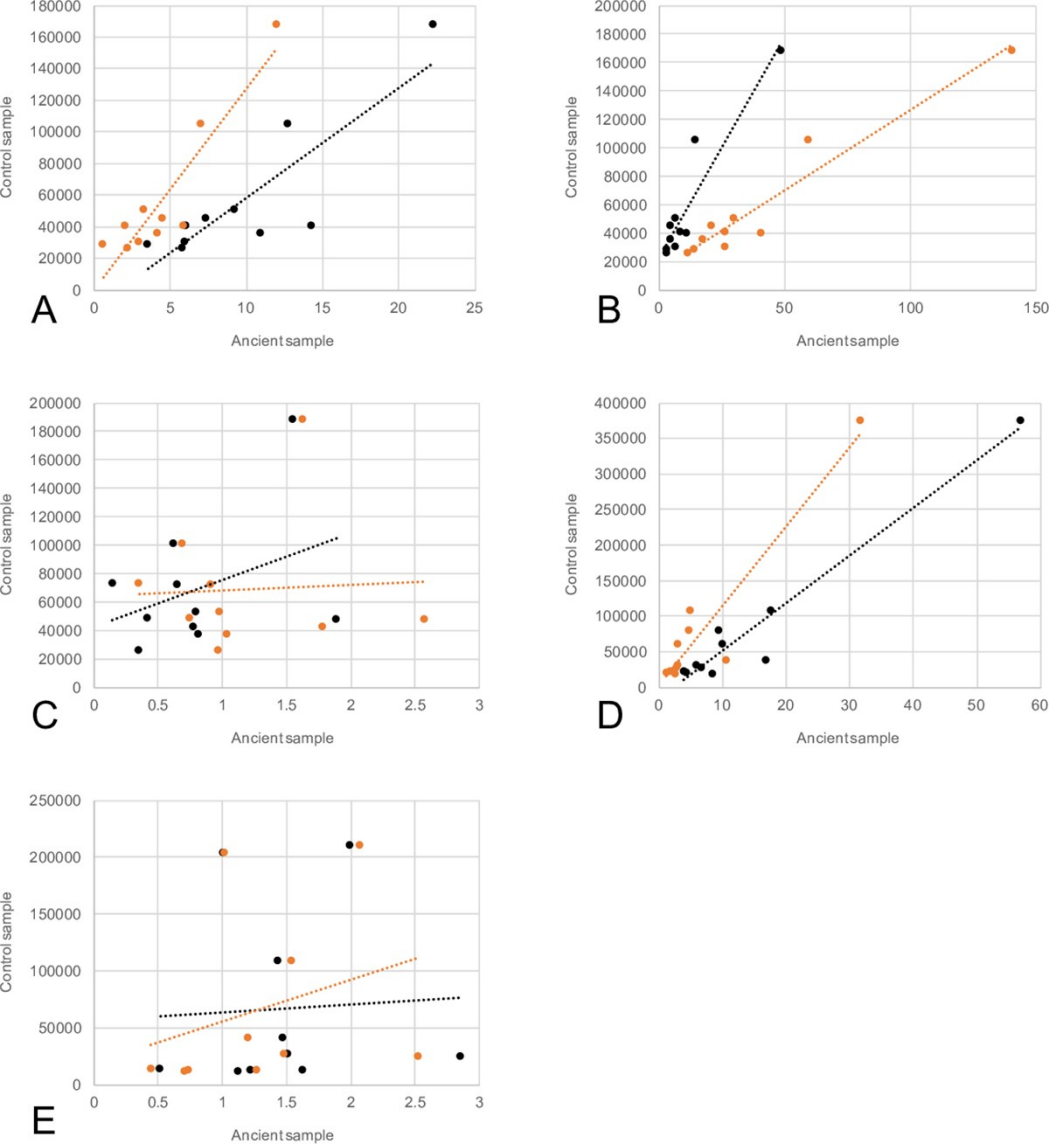
Regressions of all samples, Method 2: Relationships between the 95th percentile of expressed genes in ancient tissues (x-axis) versus control samples (y-axis). Values are calculated based on per-tissue scores (see Methods), having removed duplicate reads from mapping data. Black data points and trend line refer to BGISEQ-500 data, while orange data points and trendline refer to Illumina HiSeq-2500 data. (A) Skin 1, (B) Skin 2, (C) Tumat cartilage, (D) Tumat liver, and (E) Tumat muscle. The underlying data for this figure can be found in S7 Data, ‘Regressions’ tab.

Like our observations from Method 1, we found that the historical Skin 2 and the ancient Tumat liver tissues showed significantly more similarity to their modern control counterparts than the other historical/ancient tissues (Fig 2; S4 Data; S5 Data). Of the 14,300-year-old Tumat samples, we found virtually no correlation between ancient and control data when compared with the canine normal tissue array (Method 2) using muscle (*r*^2^ = 0.07) and cartilage (*r*^2^ = 0.01). However, we observed a high degree of similarity with liver tissue, when similarly compared with modern data (*r*^2^ = 0.94, Fig 3; S7 Data). We noted that several highly expressed genes in the ancient liver tissue are associated with liver function, including apolipoproteins, fetuins, and retinol-binding proteins.

A high level of similarity between historical and modern skin tissues (*r*^2^ = 0.70 for Skin 1 and 0.87 for Skin 2) was also observed using Method 2 (Fig 3; S7 Data). We noted that highly expressed genes in both ancient and controls are associated with skin and connective tissue, including collagen and several keratin-producing genes (S4 Table).

### Long-fragment aRNA and ‘simulated ancient’ datasets

To test whether ultrashort fragments seriously affect the tissue assignations by way of spurious mapping, we repeated analysis of ‘Method 1’, ‘Method 2’, and damage profiles on aRNA reads that were 30 nt or greater. To do this, we simply selected mapped reads ≥30 nt in length from existing BAM files, then reran coverage calculations as described for Method 1 (S3 Data) and Method 2 (S6 Data), and ran mapDamage v2.0.9 on each new BAM to check for differences when ultrashort fragments were removed (S3 Fig). For Method 1, we found that any similarity between modern and ancient data was severely reduced; for example, the proportion of expressed genes in liver tissue with duplicates removed went from 90% to 14% when ultrashort fragments were removed (S2 Data and S3 Data, S11 Fig). We noted a less extreme decrease for Skin 2, from 99% to 89%, which could be explained by a generally higher proportion of the original data being of longer fragments for Skin 2, resulting in a less drastic loss of data. For Method 2, we found that samples Skin 2 and Tumat liver still showed a high level of similarity to their modern controls (*r*^2^ = 0.84 and 0.81, respectively) and also a high degree of similarity to their short-read-included counterparts (S5 Table). Interestingly, we noticed that the Skin 1 sample, when filtering out ultrashort reads, showed a significant drop in similarity to both skin control and original Skin 1 aRNA, suggesting that in certain circumstances, ultrashort molecules have enough mapping complexity to be crucial to identifying tissue specificity.

To further validate the use of ultrashort fragments, we took the control datasets and created a ‘simulated ancient’ dataset for each, ranging from 15 to 50 nt in length, deriving from the original control data, for all 4 control samples. We then ran these new ‘simulated ancient’ data through our Method 2 pipeline, for comparison to others. We found that the simulated dataset had almost identical tissue identifiers to the original control samples (S6 Table, S12 Fig), again suggesting that ultrashort molecules do not necessarily result in spurious mapping, where the complexity is great enough.

### Metagenomic analysis

To explore microorganism presence and further validate the authenticity of our RNA reads, we performed four metagenomic analyses; first, on the tRNA fraction, to validate the origin of the data as being canine due to the relatively high interspecies sequence divergence of tRNA. We found that in all cases, the vast majority (>86.5%) of reads were assigned either directly or directly basal to canine tRNA, further suggesting the authenticity of our data. We further checked this method by checking for overlap (sharing) between tRNA-assigned and rRNA-assigned reads. In all cases, we found zero overlapping reads, again suggesting authenticity.

Secondly, we performed a full metagenomic BLAST against the NCBI nt database using the blast+ 2.6.8 suite, using a random subset of 100,000 reads for each sample. We modified the parameters to include ultrashort fragments by minimising the word score to 10 and collated reads falling at the *Canis lupus* node, and to compensate for highly conserved sequences, all those falling at or within the Mammalia node. We found that for samples Skin 2 and Tumat liver, the levels of expectedly assigned reads were high—at least four times those of the other tissues (S6 Table).

Thirdly, we repeated the full metagenomic BLAST against the nt database as above, only subsampling reads that mapped to the CanFam3.1 transcriptome during initial mapping. We found proportionally expected assignations to canis and mammal nodes, in particular for Skin 2 and Tumat liver samples (S6 Table). Blank assignations were negligible.

Lastly, we looked for evidence of viral infection from RNA viruses (both single-stranded RNA [ssRNA] and double-stranded RNA [dsRNA]) in all the sequenced tissues, noting that previous aRNA work has revealed RNA viral genomes in ancient material [11,31]. We found no evidence of viral sequences in our RNA data.

## Discussion

Our results show the proof of principle that under permafrost conditions, tissue-specific transcriptome profiles are potentially recoverable from mammalian soft tissues preserved over thousands of years. Because the survival of RNA for such long periods of time is unexpected, verification of the data’s authenticity is important. By comparing the RNA data to equivalent DNA data and assessing key characteristic differences between aRNA and aDNA data, such as reads mapping splice junctions versus exon-exon junctions (S7 Table), the quantity of ribosomal RNA (rRNA) in the samples, and overall transcriptome enrichment, we have shown the expected differences to be present and thus believe the data presented here are truly representative of aRNA.

We suggest that in contexts conducive to biomolecular preservation, aRNA (or ‘palaeotranscriptome’) analysis could provide a number of standard additional facets to the biomolecular archaeological toolkit. With further research, we anticipate these could be expanded to include tissue identification, metagenomic palaeopathology of RNA viruses, and identifying specific in vivo processes concerned with individual genomes and their underlying causes, such as climate, diet, trauma, and disease.

### Tissue specificity in ancient tissues

Our choices of primary methods to assess similarity between ancient and modern tissues were informed by a ‘standard’ approach of statistical normalisation of raw read counts mapping to individual transcripts (Method 1), followed by a new method based on coverage depth calculation of each individual gene. We explored a second option because of the ultrashort nature of our RNA data, which is explored in more detail later in this section, in which we considered that transcripts reconstructed from heavily truncated reads would be more accurately characterised according to depth and thus take into account the transcript length. While count-based normalisation is not usually an issue for modern data, in which mapped bases are often the entire length of the read, short fragments may result in biases resulting from the length of the reference. For example, two 25-nt reads mapping to one gene would carry twice the statistical weight of one 50-nt read at the same locus, where both sizes would be present in an aDNA/aRNA dataset.

Of the 2 historical skin samples and 3 ancient tissue samples, 2 samples (Skin 2 and Tumat liver) exhibited signals strongly associated with their modern counterparts. The ancient liver sample in particular, despite being the oldest of the three individuals, showed the greatest similarity to its control sample. Of particular note is that when compared with the reference Affymetrix array using Method 2, prior to comparative analysis with the control sample, 80% of the 10 most abundant transcripts and 50% of the 50 most abundant transcripts are biologically sensible, i.e., are genes primarily associated with liver tissue. Within those 50, 5 were class A and C apolipoprotein isoforms involved in lipid transport and, crucially, synthesised within the liver [32]. Three different isoforms of alpha-2 glycoprotein, associated with liver function in mammals [33], were present (see S4 Data), as were several fibrinogen and fetuin-B genes, which are also liver derived [34,35]. While simple identifications such as these are by no means conclusive, we took them as a starting point to perform more detailed statistical analyses. However, we noted that far from being an isolated incident, other, different tissues exhibited similar superficial equivalence to their controls. The Skin 2 sample contained 19 keratin-associated isoforms within the most abundant 50 transcripts, alongside several proline-rich proteins, both of which are associated with dermal tissue. Several microRNA genes were also highly represented, although a reference set for canine microRNA tissue specificity does not include skin [36], and so concrete conclusions about those transcripts cannot be made.

In addition to tissue differentiation, it was encouraging to note that in all tissues, the most highly expressed gene without tissue-specific assignment in our scoring matrix was the RNA Component of Signal Recognition Particle 7SL1 (RN7SL1) cytoplasmic RNA, which forms part of the ribosomal nucleosome complex. In highly degraded tissues, the significant presence of rRNA is expected [15] and therefore is further evidence of RNA enrichment. rRNA itself accounted for between 5.7% and 39.4% of the reads, again with no obvious correlation to tissue type or age, but again with similar results between sequencing platforms (*r*^2^ = 0.90). Similarly, all ostensibly connective tissues included a predicted collagen alpha-like gene (LOC102152155) as the second- or third-most expressed locus, although a specific named homologue could not be identified for downstream statistical analysis.

### aRNA preservation in permafrost and historical tissues

While the sample set is small, we noted that the ostensibly best-preserved tissue in the Tumat 2 individual is the deepest (liver), and the least well-preserved is the most superficial (cartilage). The muscle tissue, while intermediate, was closer in quality to the cartilage. Although we cannot make a confident assertion, we suspect that, at least concerning a small animal preserved in permafrost, the deepest tissues might have a higher proportion of endogenous DNA/RNA because of the fact that external microbial or other environmental activity would be initially present on the outer tissues. This is reflected in the lesser endogenous content of the outer tissues. Microbial activity on surface tissues being arrested by rapid freezing before reaching deeper tissues would also explain the higher endogenous content of the liver. It is also logical that a transcriptionally active tissue such as liver would exhibit greater specificity through time due to the absolute (as opposed to proportional) levels of nucleic acids in the tissue itself. We hypothesise that degradative enzymes in liver tissue would have no effect on the proportion of endogenous RNA, given the overall rapid freezing of the animal, as discussed above. With regards to historical samples, it is unsurprising that the older of the two skin tissues shows weaker RNA preservation, although this may have been affected by hitherto unknown and different preservation methods and individual postmortem histories.

As with any extraordinary claim, the veracity of our results is hugely important. Therefore, we analysed our RNA-seq data in conjunction with equivalent DNA data to eliminate the possibility of DNA contamination, by looking at exon-exon junctions, overall mapping proportions, biologically relevant tissue-specific transcriptome activity, and rRNA content. The results of these analyses all show compelling evidence of the authenticity of the RNA data, reinforcing once more the exceptional character of these remains for palaeobiological and palaeophysiological research on extinct mammals or ancient representatives of still extant species. Our analyses of the blank sequencing data consistently showed a lack of contamination, beginning in laboratory measurements of RNA (Table 1). We consistently observed negligible blank results from the mapped sequence data (Table 2, S1 Table) and equally negligible results from our junction analysis (S3 Table) and metagenomic analyses (S6 Table), often producing precisely zero hits. Certain analyses such as mapDamage were simply not possible due to a lack of blank data, and comparative analysis using Method 1 and/or Method 2 would produce essentially meaningless results due to their comparative nature. GC content analysis (S2 Table, S13 Fig, S1 Text) also showed nothing of note.

### The use of ultrashort fragments in palaeotranscriptomic research

A major concern of the recovered RNA was the ultrashort nature of the fragments following time-dependent degradation. The general instability of RNA compared with DNA is well known, and so it is plausible that aRNA breaks down to considerably shorter-length fragments than aDNA from the same tissues. We observed this phenomenon with these samples (S14 Fig, S15 Fig, S16 Fig, S17 Fig), in which DNA fragment length is longer than equivalent RNA at around 40 nt. Thus, we noted that a high proportion of our aRNA reads were shorter (15–30 nt) than the usually accepted minimum for aDNA (around 30 nt). Issues with mapping ultrashort reads such as these to genomic positions are prone to errors and mismapping [37], and so we decided to attempt to compensate for this rather than form our conclusions based on a heavily depleted dataset, which we thought likely to skew our interpretations as a result of excessive false-negative alignments.

Before reanalysing our data, we postulated that aRNA would be inherently less prone to mismatched or false-positive alignments than DNA because of reference complexity. The transcriptomic reference, unlike the equivalent genome, is relatively free of low-complexity or repeat regions often found in intergenic or untranscribed regions. We have had previous success in reconstructing aRNA genomes using such ultrashort molecules to high coverage [11], and so we decided to use a similar size threshold to analyse the data presented here. However, to confirm that mismapping of ultrashort reads was not introducing errors into our results, we performed several additional analyses by creating two new datasets from our original data. One analysis, for aRNA samples only, contained only aRNA reads greater than 30 nt in length (‘long-fragment aRNA’).

A second analysis was based on the modern control data, which has an almost universal >200-nt insert size (as would be expected from fresh RNA-seq data generated on 100 nucleotide paired-end [PE]), and so ‘native’ fragment patterns cannot be analysed for comparison. We therefore simulated an equivalent aRNA dataset by randomly sampling reads and fragmenting them in silico to equal numbers of predefined reads between 15 and 50 nt in length (‘simulated ancient’). We then repeated our analyses for tissue specificity and damage profiles and compared the altered data results with the originals.

Our reanalysis revealed an extremely strong correlation between the ‘original’ control datasets and their ‘simulated ancient’ counterparts (see S12 Fig and S6 Table), with *r*^2^ values of between 0.88 and 0.99, suggesting that our method for establishing tissue specificity is unaffected by ultrashort reads in which the reference is complex enough. Conversely, our finding that the correlation between ‘long-fragment aRNA’ and the ‘original’ control was universally lower, particularly in samples in which tissue specificity could not be established (Skin 1, Tumat cartilage, and Tumat muscle), suggests that ultrashort reads in fact improve the accuracy of tissue calling. The loss of signal in Method 1 following removal of ultrashort reads further suggests that retention of ultrashort reads in some cases is justified and necessary.

We also noted that of the two ancient samples that showed strong correlation between their controls, they were usually more similar to the unedited original control samples than to the ‘long-fragment aRNA’ datasets (S6 Table). We speculate that the poor correlation between long-fragment aRNA and controls in the three poorer-quality samples could be due to a lack of data, because the majority of those were under 30 nt. Because the aRNA original showed marginally higher correlation to the original control than to the ‘simulated ancient’, we further suspect that ultrashort reads are valid, in certain circumstances. Although data are lacking, we noted that the tissues showing greater potential for this method all have reasonably high endogenous content, which could be a proxy for overall RNA integrity. The fact that the control samples showed almost identical profiles to the ‘simulated control’ samples, these samples deriving from close to 100% endogenous content, would support this. However, considerations such as postmortem damage and the effect of these lesions on mapping ultrashort fragments should be considered further (see S1 Text).

The results of the junction analysis further suggest the authenticity of our data; as previously discussed, reads only crossing junction boundaries were considered in our calculations, as opposed to concentrations of reads nearby these boundaries. Overall, we found enrichment for exon-exon boundaries to be several orders of magnitude greater than splice junctions in our RNA dataset, and in most cases, several orders of magnitude less in the DNA data. Some DNA samples showed comparable (but nonetheless smaller) numbers of splice junction to exon-exon boundaries, for reasons unknown, although we speculate that because RNases are not routinely used in aDNA preparations, these ‘phantom’ RNA reads may be the result of RNA duplexes forming sticky-end double-stranded RNA and subsequently being incorporated into DNA library preparations. We do not, however, believe that this detracts from the interpretation of our aRNA as authentic, based on the RNA junction data.

Damage profiles of the ‘long-fragment’ aRNA sets (i.e., containing reads only over 30 nt) showed slightly clearer damage patterns than the original datasets, with C > U misincorporations at both ends, as previously observed [11] with low-level G > A misincorporations at the 3′ end (S4 Fig, S5 Fig). The introduction of ultrashort molecules apparently masks the damage signals, possibly due to damaged reads being discarded where the seed length is too long to accommodate a positive alignment and a terminal base modification. However, our confirmation of tissue specificity in other analyses suggests that these ultrashort reads contain valuable information and so should not be discarded. Instead, we recommend isolation of longer fragments such as those above 30 nt for damage validation and authentication, and using complete ultrashort datasets for transcriptome analysis later on.

### The future of aRNA

Research using ancient biomolecules is moving in leaps and bounds, breaking barriers particularly in terms of throughput, sample age, starting material, and the range of biomolecules at our disposal. aRNA, although touched upon in very recent literature, is still relatively unstudied. Perceptions about what aRNA can inform us about that DNA or proteins cannot, and a more general instability, lead many to dismiss it as unlikely and unnecessary. These data represent the oldest aRNA from any source to be recovered and sequenced, by a significant margin of at least 13,000 years, and show that under some conditions, aRNA can remain intact well enough to identify specific transcriptomic profiles approximately 9,000 years earlier than the current oldest isolated (unsequenced) aRNA. Previous research in plants has identified the potential to uncover aRNA viruses and monitor in vivo activity in long-dead organisms, and this may also be true of animal tissues. However, we stress that these were exceptionally well preserved and not prone to typical enzymatic or autolytic process that occur in mammalian decomposition, and so cannot be considered to be an ‘average’ representation of nucleic acid preservation in ancient soft tissues. This research does, however, suggest that in certain circumstances, the processes of autolysis and putrefaction can be sufficiently arrested in permafrost animal remains, and as such, in vivo processes can now be identified in samples of great interest to current research themes. This potential may not be limited to permafrost samples but might extend to other low-temperature climates such as Greenland, Alaska, Canada, and Antarctica. Equally, source material need not be limited to soft tissues; as previous research has shown, a variety of organic materials are potential sources of aRNA (most notably seed endosperm), and so there is potential to explore aRNA preservation in bone, keratin, or even sediments from such environs. Of course, data for ancient metatranscriptomics are nonexistent at the time of writing, and consequently no such assumptions can be made until further research has been done. Optimistically, we anticipate that other biomolecular analysis may be used to complement and cement our understanding of in vivo processes; for example, quantitative palaeoproteomic approaches, still in their infancy, could be enhanced using relative transcriptome data. Additionally, stable isotope data could further be complemented by these data; nitrogen isotopic analysis of different tissues indicate that Tumat puppy#2 was still sucking its mother’s milk when it died, and so it may be possible, with more samples, to establish individual developmental stages through transcriptomic and isotopic complementary data.

In conclusion, we suggest that as an untapped biomolecular resource, aRNA has potential to enrich the current body of palaeogenomic study. Although still a field very much in its infancy, aRNA study not only has the potential to provide verification for tissue identification but also to enhance or validate other areas of biomolecular archaeological research, such as epigenomics, palaeoproteomics, and stable isotope analysis. Continuing the palaeopathological perspective, we note that several viruses of importance historically and in modernity such as HIV, yellow fever, West Nile virus, ebola, rabies, hepatitis C, influenza, and measles have RNA genomes. The potential value in establishing their evolutionary trajectories, along with the aforementioned in vivo processes, makes clear the future utility of aRNA.

## Methods

### Ethics statement

This study utilises tissues from vertebrate animals (wolves). However, the ancient nature of these samples means that no ethical issues arise from this work.

### Samples

To explore the viability of aRNA survival, we chose samples considered to have varying potential for success, given endogenous DNA content from previous genome analysis [25], but with at least two with a subjectively high potential. Three of the samples represent different tissues (cartilage, liver, and muscle) from the same individual: a remarkably well-preserved large canid puppy, with a calibrated radiocarbon age of 14,233 ± 34 yBP (ETH-73412; 12,297–12,047 cal BC; 95.4% probability using OxCal v4.2.4) [38], from the village of Tumat in Siberia, Russia. Two puppies were found at the Tumat site, and these analyses concern only puppy #2 (see Table 1). Full descriptions of the samples can be found in Mak and colleagues, 2017 [25]. The three tissue samples from the Tumat puppy were ideal, because they represent varying degrees of preservation from the same individual of advanced ^14^C age. The other two samples, CN214 and CN1921, are both historical skins (hides) from Greenlandic wolves, shot in 1925, and prior to 1869, respectively. Both are currently housed within the Greenland collection at the Natural History Museum of Denmark.

### Laboratory work

All pre-PCR steps of laboratory work, including RNA extraction, oligonucleotide processing, and library construction, were performed in dedicated aDNA facilities equipped with anteroom and positive air pressure. The aDNA facility is physically isolated from PCR areas. All standard approaches to working with ancient biomolecules (PPE clothing, double-layered gloves, deep cleaning, facemasks, etc.) were followed. aDNA laboratory guidelines are, in principle, very similar to (if not more stringent than) standard RNA practices; in any case, all plasticware and reagents used were nuclease-free, and all surfaces were kept sterilised at all times. For all steps of pre-PCR work, laminar flow and fume hood cabinets were used when appropriate.

### RNA extraction and purification

Extraction and library construction were performed around protocols designed towards microRNA, due to the presumption that it would be necessary to isolate and sequence ultrashort fragments from ancient assemblages, given that RNA fragmentation is a time-dependent diagenetic process [11,15]. RNA was isolated from tissues using an Ambion miRvana kit, following the protocol for total RNA isolation, with the following modifications: prior to digestion, tissues were flash frozen in liquid nitrogen and ground to powder using a mortar and pestle. Tissue powder was then incubated in 1 mL of Lysis/Binding buffer for 65 hours at 37°C. Organic extraction with acidic pH 4.2 phenol:chloroform was done to enable phase separation of RNA and DNA [39]. We opted for this method over DNase treatment because we have previously observed significant inefficiencies of DNase when using aDNA as a substrate, often resulting in partial digestion of RNA [40]. We performed organic extraction twice to ensure the purity of RNA, as described [41]. All other steps were performed according to the manufacturer’s instructions; briefly, salt-based precipitation was initiated using a proprietary salt mixture, and consolidated with excess ethanol. RNA was then isolated on a spin-column-attached silica membrane, which was then washed three times using included buffers. RNA was eluted in 50 μL, applied at 95°C as per the recommended protocol. The quantity of purified RNA was measured using the Qubit RNA HS assay. Due to known and suspected issues in fluorescence quantification in degraded or fragmented nucleic acid extractions [42], a DNA measurement was not taken using Qubit. We instead opted to measure the level of DNA carryover by quantifying the level of mapping to untranscribed regions of the genome, defining the untranscribed regions as the inverse of any and all transcripts, coding or otherwise, as defined from the CanFam3.1 genome annotation (gff) file. We subsequently elected to build platform-specific RNA libraries and sequence on two different platforms, the Illumina HiSeq-2500 and the BGISEQ-500, to allow us to explore platform-dependent biases in data generation alongside establishing the survival of aRNA.

### Illumina library construction

cDNA libraries were constructed using a NEBNext Multiplex Small RNA Library Prep Set for Illumina according to the manufacturer’s instructions. We opted for this method over other RNA library preparations because of the increased specificity of RNA molecules being incorporated into the library and proven sequence recovery of ultrashort molecules [43]. Briefly, a pre-adenylated 3′ adapter is first ligated to the 5′ end of the RNA molecule. This ATP-free ligation step is facilitated by an RNA ligase mutant, which is truncated to prevent RNA adenylation and thus ligation, unless pre-adenylation of the donor molecule has already occurred [44]. This takes advantage of the 3′ hydroxyl group unique to RNA and thus facilitates enrichment of RNA over potential contaminant DNA. Next, a reverse transcription primer is annealed to the 3′ adapter. Then, a standard ssRNA ligation step allows ligation of the 5′ adapter to the RNA molecule to be amplified. Reverse transcription to create single-indexed cDNA libraries based on the RT primer is followed by indexing PCR. Libraries were amplified with between 16 and 20 cycles of PCR using the included polymerase mastermix and submitted directly for sequencing.

### BGISEQ-500 library construction

For BGISEQ-500 libraries, we utilised the same NEBNext kit with modified adapters and primer oligos appropriate to the BGISEQ-500 platform. We based oligo sequences on those published previously [25] and utilised indexing primers over indexing adapters to reduce costs and improve protocol simplicity, opting for a single 5′ phosphorylated 5′ adapter and adenylated 3′ adapter. Because 5′ adenylation of the 3′ adapter is necessary to RNA-specific library construction as detailed above, the custom BGISEQ-500 3′ adapter was adenylated at the 5′ end using a NEB 5′ Adenylation kit. Libraries were similarly amplified with between 16 and 20 cycles of PCR. With the BGISEQ-500 libraries only, post-PCR products were circularised to form DNA nanoballs (DNBs) based on the standard protocol for the platform [25]. DNB production was performed by BGI Europe immediately prior to sequencing.

### Sequencing

Illumina libraries were pooled at equimolar concentrations and sequenced at 80 nt, single-end (SE80), on the HiSeq-2500 platform at the Danish National High-Throughput Sequencing Centre. BGI libraries were equally pooled to equimolar concentrations, circularised, and sequenced as SE50 using the BGISEQ-500 platform at BGI Europe, Copenhagen. Demultiplexing was performed in-house, and resulting FastQ files were delivered electronically.

### Adapter removal

Illumina and BGI adapters were removed from their respective datasets using cutadapt v.1.11 [45], using default parameters for single-end reads, 10% allowed mismatch, and a minimum size retention of 15 nt.

### Read alignment

Sequencing reads from the ancient samples were initially aligned to the CanFam3.1 genome using bowtie2 [46], under default parameters for single-end data. This was done to assess the overall endogenous content, including potential DNA contaminants, and in relation to previous estimates of endogenous content of the samples [25]. Resulting SAM files were converted to sorted BAM files and filtered by mapping the quality score (minimum q = 20). The analysis was then repeated using identical parameters, only instead using the CanFam3.1 transcriptome as the reference, and again using canine rRNA and tRNA reference sequences from which to calculate the RNA enrichment factors. tRNA sequences were downloaded from GtRNAdb [47] and rRNA sequences were obtained from the Silva rRNA database [48]. Mapping files were de-duplicated, although mapping files with duplicates retained were kept for comparative analyses. Control data were aligned to the CamFam3.1 transcriptome using default parameters for paired-end data in bowtie2. We performed identical analysis on our extraction blank library and ran any mapped reads through ncbi BLAST+, using default parameters to the nt database, followed by metagenomic analysis using MEGAN [49] to ensure no contamination. From the MEGAN analysis we found that all mapped extraction blank reads returned primarily basal or highly conserved assignments, and negligible read numbers were assigned to canids for both Illumina and BGI platforms (2 reads and 39 reads), respectively.

### Junction analysis

We used tophat v2.1.2 [50] to generate an index of exon-exon junctions from the CanFam3.1 genome annotation and also to map raw, trimmed, de-duplicated RNA-seq reads back to that index. We then collated the number of reads straddling exon-exon junctions from the tophat junctions.bed output. We generated intron and exon bedfiles from the CanFam3.1 genome annotation and used the bedtools intersect function to assess the number of reads straddling splice junctions. First, we created a BAMfile of reads overlapping exon junctions from our original mapping BAMfiles and fed that output back into the bedtools intersect to repeat the analysis, using the intron bedfile instead of the exon bedfile. We used the output from this second round of bedtools intersect to collate read numbers. We then repeated this analysis using raw, trimmed DNA reads generated previously [25] to compare the two types of data.

### Damage pattern analysis

Cytosine deamination patterns of reads aligned to the CanFam3.1 transcriptome were assessed using mapDamage 2.06 [51]. While the samples had previously showed expected damage patterns from genome sequencing [25], the expectations of similar analysis for RNA are largely unknown due to factors such as single strandedness and sequence-specific secondary structure formation. We assessed damage profiles on BAM files resulting from both genomic and transcriptomic mapping.

### Control and reference data

For direct transcriptomic comparison, we analysed equivalent, modern NGS data deriving from the same four dog tissue types (skin, cartilage, liver, and skeletal muscle). Appropriate data for all tissues were found at the ENA Short Read Archive bioproject, accession PRJNA396033, experiment accessions SRX3055179 (cartilage), SRX3055151 (liver), SRX3055143 (skin), and SRX3055142 (muscle). For reference data on relative expression levels between dog tissues, we used Affymetrix array data collated from the Canine Normal Tissue Database, bioproject accession PRJNA124245 [28].

### Expression analysis

Because gene-specific expression analysis has not been performed on ancient material, we attempted two forms of analysis. Method 1 is a direct comparison of control NGS data (see ‘Control and reference data’) to ancient sequencing data. Method 2 was achieved by employing an independent, non-NGS expression array reference [28] with which both modern control NGS and ancient/historical NGS datasets would be compared. Both modern and ancient/historical data were subject to the same analysis.

Both analyses relied on first calculating a relative measure of expression for individual genes within each sample. To generate this, we used the samtools depth function to describe the coverage depth for each position of each transcript, and divided the total coverage for all positions by the length of the transcript to generate a mean coverage value for each. The unique nature of these data creates uncertainties regarding duplicate removal considering excess PCR cycles and short fragments, so we therefore opted to perform analyses using combinations of de-duplicated and duplicates-retained mapping between ancient and control samples. We found that de-duplication, in particular applied to the ancient samples, is more appropriate for these kinds of data (see Discussion).

The direct comparison method (Method 1) involved firstly performing a variance stabilising transformation on transcript raw count data, using the Varistran R package (incorporating the edgeR package) [27,52]. Varistran employs library size normalisation (by total number of reads, not fragment length) using edgeR’s TMM normalisation, then applies Anscombe's [53] variance stabilising transformation for the negative binomial distribution [27]. Because no replicates were available for each of the ancient samples or controls, dispersion was estimated across the entire dataset (blindly). These normalised data were used for comparison between samples across the entire dataset using Varistran package functions, producing ordination biplots and a distance-based heatmap with hierarchal clustering. Biplots were produced by centring rows (genes) by subtracting their global means, performing singular value decomposition, and these data were plotted; the expression level of a gene in a particular sample, relative to the average expression level of that gene, is approximated by the dot product of the sample position and the gene position (personal communication, P. Harrison). Heatmaps were produced by calculating cosine distance, performing hierarchical clustering with *hclust()*, and refining clustering using the ‘optimal leaf ordering’ algorithm from the seriation package [54] in order to minimise sharp changes between neighbours without otherwise changing the tree.

To directly compare expression levels between control and ancient/historic samples within and between tissue types, the transformed data for each tissue type were filtered for transcripts within and above the upper 95th percentile of expression levels (i.e., the most highly expressed genes for each tissue type in a given sample). Data below the 95th percentile were discarded, to compensate for noise associated with low-level transcripts [55]. Pairwise linear regression analyses were then performed comparing control tissue expression (explanatory variable) to expression in all ancient/historic tissues (response variable[s]). We corrected for multiple testing [55] using Bonferroni corrections: for each control tissue there were 5 comparisons with ancient/historic samples, so linear models were considered significant at an α of 0.01. When comparing control tissues to other control tissues, there were 3 comparisons, so linear models were considered significant at an α of 0.0166. Linear models between control samples and both ancient and other control samples were only considered relevant if their slope was positive.

For Method 2, we first created a simple reference set from the Affymetrix array deriving from the Canine Normal Tissue Database [28]. This was used to describe the tissue with which each annotated gene was most associated, for example, apolipoprotein 1 (APO1) is most associated with liver tissue, collagen is most associated with skin, etc. This resulted in a simple gene name to tissue pairing matrix describing one tissue per gene. We then created a second matrix from the CanFam3.1 transcriptome, describing the specific gene name in relation to the gene description (i.e., predicted homology or confirmed) and reference (Genbank ID) to which the data were mapped. For each sample, we took transcripts and associated Genbank references within and above the 95th percentile of expression levels (as calculated earlier using samtools depth) [27,54,55] to create a final matrix of gene, coverage, and most associated tissue. Then, for each sample, we cumulatively scored each of the 10 tissues listed in the Affymetrix array, according to the gene/tissue pairing described in matrix 1. We performed this analysis for all ancient and modern sequencing data, and compared like-for-like sample tissues using a linear regression. We used these analyses to assess the similarity of the modern and ancient datasets based on their appearance when compared with the limited tissue set represented from the Affymetrix array.

### GC content analysis

We assessed the GC content on a per-transcript basis of the CanFam3.1 transcriptome, using a Perl script. We then isolated the transcripts from within the 95th percentile of expression levels as described earlier, for consistency. Then, the GC content of individual short reads mapping to those transcripts was calculated on a per-sample basis, from de-duplicated and duplicates-retained BAM files (S2 Table).

### Metagenomic analysis

For viral infection analysis, we downloaded complete genomes for all available ssRNA and dsRNA viruses known to infect vertebrates from the NCBI Genome resource. Then, we mapped all raw reads to the virus dataset using bowtie2 and extracted the mapped reads into fasta format. We then subjected these reads to a full metagenomic BLAST to confirm their viral origin. For tRNA species authentication, we extracted all reads previously mapped to known canine tRNA sequences and performed a full metagenomic BLAST against the entire nt database. For general metagenomic analysis of mapped and raw reads, we subsampled 100,000 reads from each sample using seqtk v1.2 and ran similarly, only using a word score of 10. All BLAST analyses were performed using the NCBI blast+ v.2.6.0 suite, on a standalone high-performance cluster. Taxonomic assignations were viewed using MEGAN v5.11,3 [49].

### Construction of long-fragment aRNA and ‘simulated’ aRNA data

To assess how much ultrashort fragments affect tissue identification, we first removed all fragments under 30 nt from existing mapping BAM files using the samtools v1.4 ‘view’ function and an awk one-liner. We then repeated downstream analyses using these new BAM files as the source data.

To create a proxy for aRNA from modern data we know to map well to the reference, we selected 1 million reads at random from each of the control datasets. Using the seqtk v1.2 sample function, we piped that output through the seqtk ‘trimfq’ function with–s and–e options set to retrieve 15 bases from each randomly subsampled read. We then repeated this 34 times, each time selecting one extra base to create 35 subsets of length 15–50. These subsets were then merged and treated as a single dataset for each tissue, giving 4 simulated aRNA datasets.

## Supporting information

S1 Fig mapDamage profiles of ancient tissues mapped to the CanFam3.1 transcriptome showing nt misincorporations at relative positions from the centre towards the terminal ends of the sequencing read, using bwa-aln as primary mapper and de-duplicated reads only. (A) and (B) Skin 1; (C) and (D) Skin 2; (E) and (F) Tumat cartilage; (G) and (H) Tumat liver; (I) and (J) Tumat muscle. nt, nucleotide.
(TIF)

S2 Fig mapDamage profiles of ancient tissues mapped to the CanFam3.1 transcriptome showing nt misincorporations at relative positions from the centre towards the terminal ends of the sequencing read, using bowtie2 as primary mapper. Red lines indicate C > U misincorporations, blue lines indicate G > A misincorporations, and grey lines indicate others. (A) Skin 1, de-duplicated; (B) Skin 1, duplicates retained; (C) Skin 2, de-duplicated; (D) Skin 2, duplicates retained; (E) Tumat cartilage, de-duplicated; (F) Tumat cartilage, duplicates retained; (G) Tumat liver, de-duplicated; (H) Tumat liver, duplicated retained; (I) Tumat muscle, de-duplicated; (J) Tumat muscle, duplicates retained. Derived from BGISEG-500 data. The underlying data for this figure can be found in S1 Data. nt, nucleotide.
(TIF)

S3 Fig mapDamage profiles of ancient tissues mapped to the CanFam3.1 transcriptome showing nt misincorporations at relative positions from the centre towards the terminal ends of the sequencing read, using bowtie2 as primary mapper. Red lines indicate C > U misincorporations, blue lines indicate G > A misincorporations, and grey lined indicate others. (A) Skin 1, de-duplicated; (B) Skin 1, duplicates retained; (C) Skin 2, de-duplicated; (D) Skin 2, duplicates retained; (E) Tumat cartilage, de-duplicated; (F) Tumat cartilage, duplicates retained; (G) Tumat liver, de-duplicated; (H) Tumat liver, duplicated retained; (I) Tumat muscle, de-duplicated; (J) Tumat muscle, duplicates retained. Derived from HiSeq-2500 data. The underlying data for this figure can be found in S1 Data. nt, nucleotide.
(TIF)

S4 Fig mapDamage profiles of ancient tissues mapped to the CanFam3.1 transcriptome sequenced on BGISEQ-500 showing nt misincorporations at relative positions from the centre towards the terminal ends of the sequencing read, using bowtie2 as primary mapper, de-duplicated reads only, and only incorporating reads of 30 nt or greater. (A) Skin 1; (B) Skin 2; (C) Tumat cartilage; (D) Tumat liver; (E) Tumat muscle. The underlying data for this figure can be found in S1 Data. nt, nucleotide.
(TIF)

S5 Fig mapDamage profiles of ancient tissues mapped to the CanFam3.1 transcriptome sequenced on HiSeq-2500 showing nt misincorporations at relative positions from the centre towards the terminal ends of the sequencing read, using bowtie2 as primary mapper, de-duplicated reads only, and only incorporating reads of 30 nt or greater. (A) Skin 1; (B) Skin 2; (C) Tumat cartilage; (D) Tumat liver; (E) Tumat muscle. The underlying data for this figure can be found in S1 Data. nt, nucleotide.
(TIF)

S6 Fig Hierarchical clustering heatmap of similarity between samples (see Methods for details) for the top 500 genes with the most differences between samples. (A) BGISEQ-500 data, de-duplicated; (B) HiSeq-2500 data, de-duplicated; (C) BGISEQ-500 data, duplicates retained; (D) HiSeq-2500 data, duplicates retained.
(TIF)

S7 Fig Regressions for samples sequenced on the BGISEQ-500 platform, de-duplicated, Method 1. (A) comparison to skin; (B) comparison to cartilage; (C) comparison to liver; (D) comparison to muscle. See legend for Fig 1 for details.
(TIF)

S8 Fig Regressions for samples sequenced on the BGISEQ-500 platform duplicates retained, Method 1. (E) Comparison to skin; (F) comparison to cartilage; (G) comparison to liver; (H) comparison to muscle. See legend for Fig 1 for details.
(TIF)

S9 Fig Regressions for samples sequenced on the HiSeq-2500 platform, de-duplicated, Method 1. (I) Comparison to skin; (J) comparison to cartilage; (K) comparison to liver; (L) comparison to muscle. See legend for Fig 1 for details.
(TIF)

S10 Fig Regressions for samples sequenced on the HiSeq-2500 platform, duplicates retained, Method 1. (M) Comparison to skin; (N) comparison to cartilage; (O) comparison to liver; (P) comparison to muscle. See legend for Fig 1 for details.
(TIF)

S11 Fig Regressions for Skin 2 and liver samples, Method 1, using only reads of 30 nt or greater. (A) Skin 2; (B) Tumat liver. nt, nucleotide.
(TIF)

S12 Fig Regression of coverage: control data versus ‘simulated ancient’ control data using data points from Method 2. (A) Skin; (B) liver; (C) muscle; (D) cartilage. The underlying data for this figure can be found in S8 Data.
(TIF)

S13 Fig GC content histograms according to sequencing platform and duplicate removal. For all panels: blue line, Skin 1; orange line, Skin 2; grey line, Tumat cartilage; yellow line, Tumat liver; black line, Tumat muscle; green line, blank. (A) BGISEQ-500, duplicated removed; (B) HiSeq-2500, duplicated removed; (C) BGISEQ-500, duplicates retained; (D) HiSeq-2500, duplicates retained.
(TIF)

S14 Fig Length distribution plots of BGISEQ-500 RNA-seq. (A) Skin 1; (B) Skin 2; (C) Tumat cartilage; (D) Tumat liver; (E) Tumat muscle. The underlying data for this figure can be found in S9 Data. RNA-seq, RNA sequencing.
(TIF)

S15 Fig Length distribution plots of HiSeq-2500 RNA-seq. (A) Skin 1; (B) Skin 2; (C) Tumat cartilage; (D) Tumat liver; (E) Tumat muscle. The underlying data for this figure can be found in S10 Data. RNA-seq, RNA sequencing.
(TIF)

S16 Fig Length distribution plots of BGISEQ-500 DNA-seq from Mak and colleagues. (A) Skin 1; (B) Skin 2; (C) Tumat cartilage; (D) Tumat liver; (E) Tumat muscle. The underlying data for this figure can be found in S11 Data. DNA-seq, DNA sequencing.
(TIF)

S17 Fig Length distribution plots of HiSeq-2500 DNA-seq from Mak and colleagues. (A) Skin 1; (B) Skin 2; (C) Tumat cartilage; (D) Tumat liver; (E) Tumat muscle. The underlying data for this figure can be found in S12 Data. DNA-seq, DNA sequencing.
(TIF)

S18 Fig mapDamage plots of DNA data from Mak and colleagues, 2018. (A–E) Sequenced on the BGISEQ-500 platform. (F–J) Sequenced on the HiSeq-2500 platform. (A) Skin 1; (B) Skin 2; (C) Tumat cartilage; (D) Tumat liver; (E) Tumat muscle. (F) Skin 1; (G) Skin 2; (H) Tumat cartilage; (I) Tumat liver; (J) Tumat muscle. Red lines, frequency of C > U misincorporations; blue lines, frequency of G > A misincorporations; yellow lines, soft-clipped bases from unaligned reads; grey lines, other misincorporations.
(TIF)

S19 Fig Comparison of data generated by BGISEQ-500 and HiSeq-2500 platforms. (A) Endogenous content of sequencing reads by tissue (see S4 Table). (B) Regressions of Method 2 between platforms. Red circles, Skin 1; white circles, Tumat cartilage; blue circles, Skin 2; black circles, Tumat liver; grey circles, Tumat muscle. (C) Mean GC content of reads by tissue, depending on duplication. Red triangles, reads mapping to the 95th percentile and above of expression after mapping and de-duplication. White triangles, all mapped reads with de-duplication. Grey triangles, all mapped reads without de-duplication. (D) RNA enrichment factor by tissue type. The underlying data for this figure can be found in S13 Data.
(TIF)

S20 Fig Biplot ordination of standardised individual gene expression (blue points) and similarity between individual samples (red points) along two dimensions (see Methods for details). (A) BGISEQ-500 data, de-duplicated; (B) HiSeq-2500 data, de-duplicated; (C) BGISEQ-500 data, duplicates retained; (D) HiSeq-2500 data, duplicates retained. All sample labels ending ‘cntl’ are modern controls. All sample labels ending ‘rmd’ are duplicate-removed samples. Cart, cartilage; ill, Illumina sequencing; Liv, liver; Mus, muscle.
(TIF)

S1 Table Basic mapping statistic comparison of aligners bwa-aln and bowtie2. (TIF)

S2 Table Mean GC content of mapped reads depending on selection and (de)duplication. (TIF)

S3 Table Junction analysis of RNA-seq and DNA data derived from the same samples. Reads mapping over splice junctions and exon-exon junctions were collated for each sample and molecule type, and enrichment factors calculated. In all cases, RNA-seq data show significantly more exon-exon junction coverage than splice junctions, highlighting their authenticity. Conversely, the opposite trend is seen for DNA data. RNA-seq, RNA sequencing.
(TIF)

S4 Table Method 2 final scores according to Affymetrix array tissue derived from modern and ancient NGS datasets. Top half, scores following de-duplication. Lower half, scores with duplicate reads retained. NGS, next-generation sequencing.
(TIF)

S5 Table Regression (*r*^2^) values for comparisons resulting from Method 2. The three tissues most similar to their modern counterparts are highlighted in bold.
(TIF)

S6 Table Taxonomic assignments from metagenomic BLAST analysis, of both mapped and raw data reads. (TIF)

S7 Table Basic NGS statistics of DNA data, subjected to the same analysis as the RNA-seq data of the same samples. Note that the rRNA proportion and overall RNA enrichment factors are significantly less than those of the RNA-seq data. NGS, next-generation sequencing; RNA-seq, RNA sequencing; rRNA, ribosomal RNA.
(TIF)

S8 Table Mean and standard deviations of coverage to the dog 18s rRNA. rRNA, ribosomal RNA.
(TIF)

S1 Data mapDamage outputs corresponding to S2 Fig, S3 Fig, S4 Fig and S5 Fig. (ZIP)

S2 Data Regression table of Method 1. Details of linear regression analysis of the 95th percentile of genes expressed in each control tissue, compared with each ancient tissue and other control tissues. Models marked in bold have the slope in the expected direction (positive) and are significant at Bonferroni alphas adjusted for multiple comparisons (ancient tissues alpha = 0.01, control tissues alpha = 0.0166).
(XLSX)

S3 Data As S2 Data, with only reads of length 30 nt or greater. Details of linear regression analysis of the 95th percentile of genes expressed in each control tissue, compared with each ancient tissue and other control tissues. Models marked in bold have the slope in the expected direction (positive) and are significant at Bonferroni alphas adjusted for multiple comparisons. nt, nucleotide.
(XLSX)

S4 Data Scoring matrix for Method 2 arranged in tabs by tissue and sequencing platform. Briefly, columns A and B are the static tissue/gene pairs generated from the CNTD Affymetrix array. Column D is the NCBI reference for each gene found on the CanFam3.1 transcriptome, column F the full gene description, and column G the derived gene name/locus (LOC) ID. Column E is the mean coverage depth of that gene after mapping. Column H is a lookup formula to assign each gene a most-related tissue from the 10 listed on CNTD. Column I is the 95th percentile value of coverage. Columns J–S are the total cumulative scores assigned to each of the 10 tissues following associated-gene/score pairing. CNTD, Canine Normal Tissue Database.
(XLSX)

S5 Data Scoring matrix for Method 2, as S4 Data, only for modern control data only. Briefly, columns A and B are the static tissue/gene pairs generated from the CNTD Affymetrix array. Column D is the NCBI reference for each gene found on the CanFam3.1 transcriptome, column F the full gene description, and column G the derived gene name/locus (LOC) ID. Column E is the mean coverage depth of that gene after mapping. Column H is a lookup formula to assign each gene a most-related tissue from the 10 listed on CNTD. Column I is the 95th percentile value of coverage. Columns J–S are the total cumulative scores assigned to each of the 10 tissues following associated-gene/score pairing. CNTD, Canine Normal Tissue Database.
(XLSX)

S6 Data As S4 Data, with only reads of length 30 nt or greater. Scoring matrix for Method 2 arranged in tabs by tissue and sequencing platform. Briefly, columns A and B are the static tissue/gene pairs generated from the CNTD Affymetrix array. Column D is the NCBI reference for each gene found on the CanFam3.1 transcriptome, column F the full gene description, and column G the derived gene name/locus (LOC) ID. Column E is the mean coverage depth of that gene after mapping. Column H is a lookup formula to assign each gene a most-related tissue from the 10 listed on CNTD. Column I is the 95th percentile value of coverage. Columns J–S are the total cumulative scores assigned to each of the 10 tissues following associated-gene/score pairing. CNTD, Canine Normal Tissue Database; nt, nucleotide.
(XLSX)

S7 Data Raw data corresponding to Fig 3. (XLSX)

S8 Data Raw data corresponding to S12 Fig. (XLSX)

S9 Data Raw data corresponding to S14 Fig. (XLSX)

S10 Data Raw data corresponding to S15 Fig. (XLSX)

S11 Data Raw data corresponding to S16 Fig. (XLSX)

S12 Data Raw data corresponding to S17 Fig. (XLSX)

S13 Data Raw data corresponding to S19 Fig. (XLSX)

S14 Data As S4 Data, only with duplicate reads retained in the analysis. (XLSX)

S1 Text Further discussion of the metagenomic analyses, RNA damage profiles, issues surrounding sequence duplication and aRNA datasets, GC content of aRNA, and a comparison of the two sequencing platforms used in this study. aRNA, ancient RNA.
(DOCX)

## Abbreviations

aDNA: ancient DNA
APO1: apolipoprotein 1
aRNA: ancient RNA
DNB: DNA nanoball
dsRNA: double-stranded RNA
EBI: European Bioinformatics Institute
NGS: next-generation sequencing
nt: nucleotide
PE: paired-end
qPCR: quantitative PCR
RNA-seq: RNA sequencing
RN7SL1: RNA Component of Signal Recognition Particle 7SL1
rRNA: ribosomal RNA
ssDNA: single-stranded DNA
ssRNA: single-stranded RNA
