# Supplementary figures and images for: Ancient RNA from Late Pleistocene permafrost and historical canids shows tissue-specific transcriptome survival
Source: PLoS Biol. 2019 Jul 30;17(7):e3000166. doi: 10.1371/journal.pbio.3000166 (PMC6667121; doi:10.1371/journal.pbio.3000166)

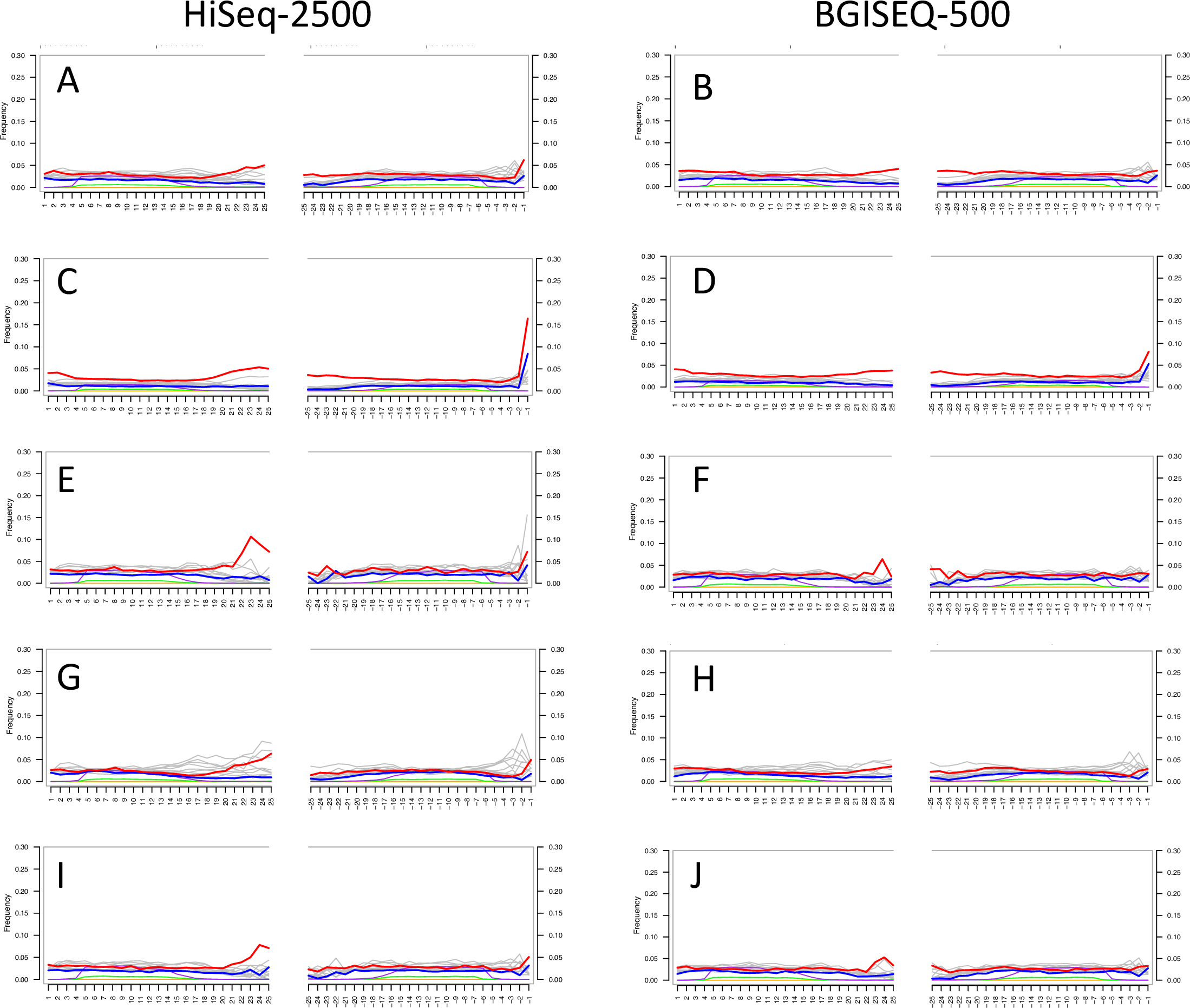

Supplement: S1 Fig — (A) and (B) Skin 1; (C) and (D) Skin 2; (E) and (F) Tumat cartilage; (G) and (H) Tumat liver; (I) and (J) Tumat muscle. nt, nucleotide. (TIF) [file pbio.3000166.s001.tif]

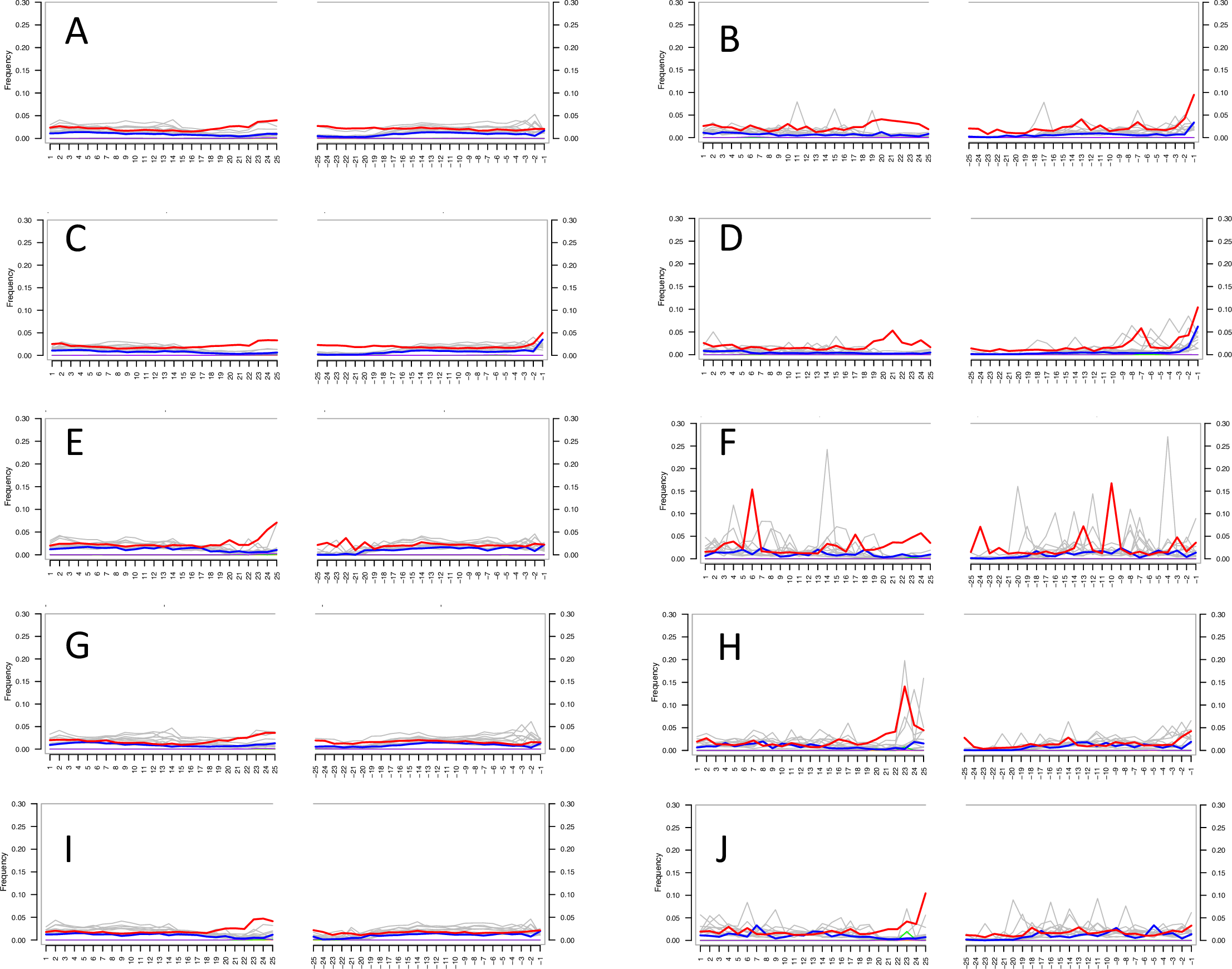

Supplement: S2 Fig — Red lines indicate C > U misincorporations, blue lines indicate G > A misincorporations, and grey lines indicate others. (A) Skin 1, de-duplicated; (B) Skin 1, duplicates retained; (C) Skin 2, de-duplicated; (D) Skin 2, duplicates retained; (E) Tumat cartilage, de-duplicated; (F) Tumat cartilage, duplicates retained; (G) Tumat liver, de-duplicated; (H) Tumat liver, duplicated retained; (I) Tumat muscle, de-duplicated; (J) Tumat muscle, duplicates retained. Derived from BGISEG-500 data. The underlying data for this figure can be found in S1 Data. nt, nucleotide. (TIF) [file pbio.3000166.s002.tif]

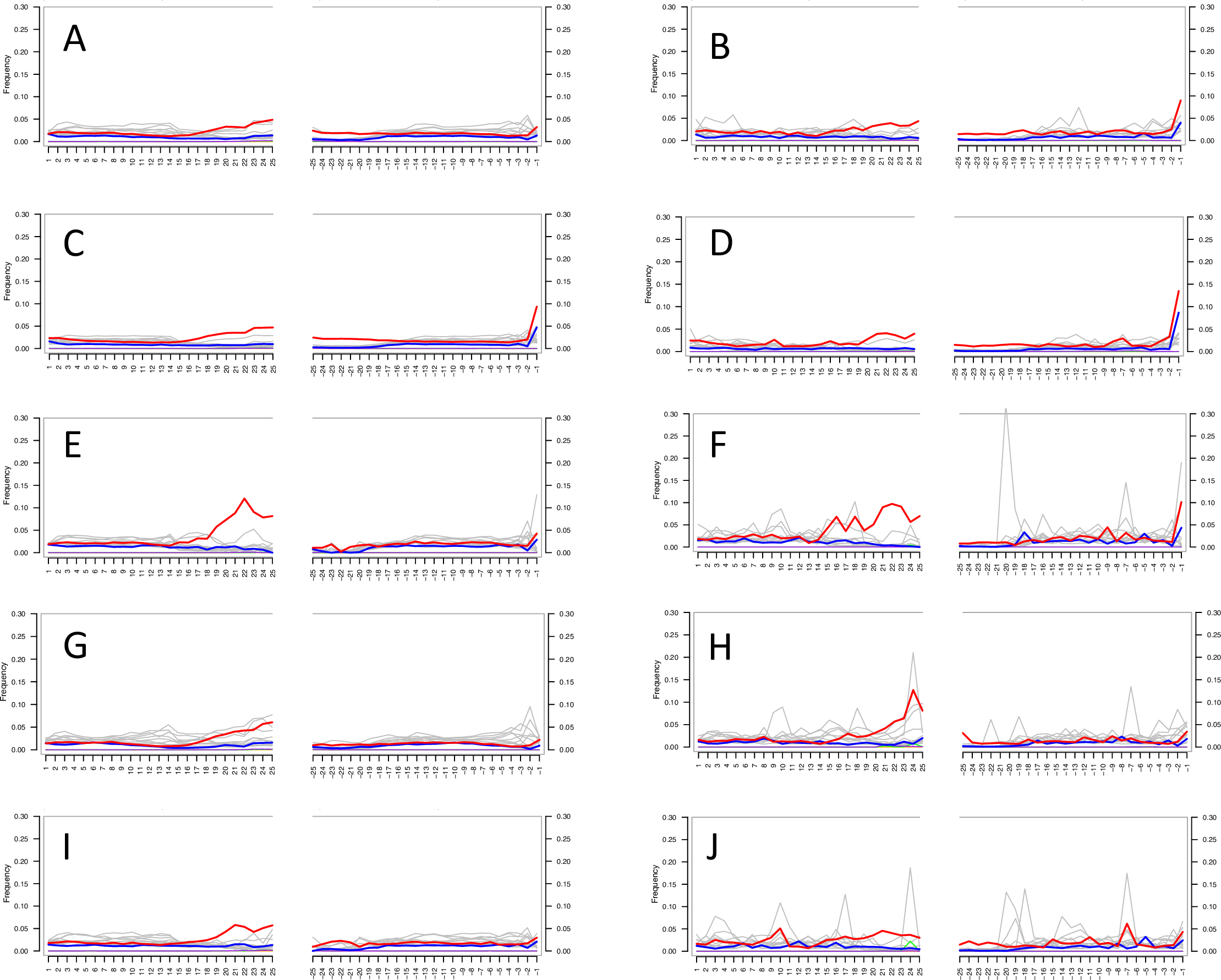

Supplement: S3 Fig — Red lines indicate C > U misincorporations, blue lines indicate G > A misincorporations, and grey lined indicate others. (A) Skin 1, de-duplicated; (B) Skin 1, duplicates retained; (C) Skin 2, de-duplicated; (D) Skin 2, duplicates retained; (E) Tumat cartilage, de-duplicated; (F) Tumat cartilage, duplicates retained; (G) Tumat liver, de-duplicated; (H) Tumat liver, duplicated retained; (I) Tumat muscle, de-duplicated; (J) Tumat muscle, duplicates retained. Derived from HiSeq-2500 data. The underlying data for this figure can be found in S1 Data. nt, nucleotide. (TIF) [file pbio.3000166.s003.tif]

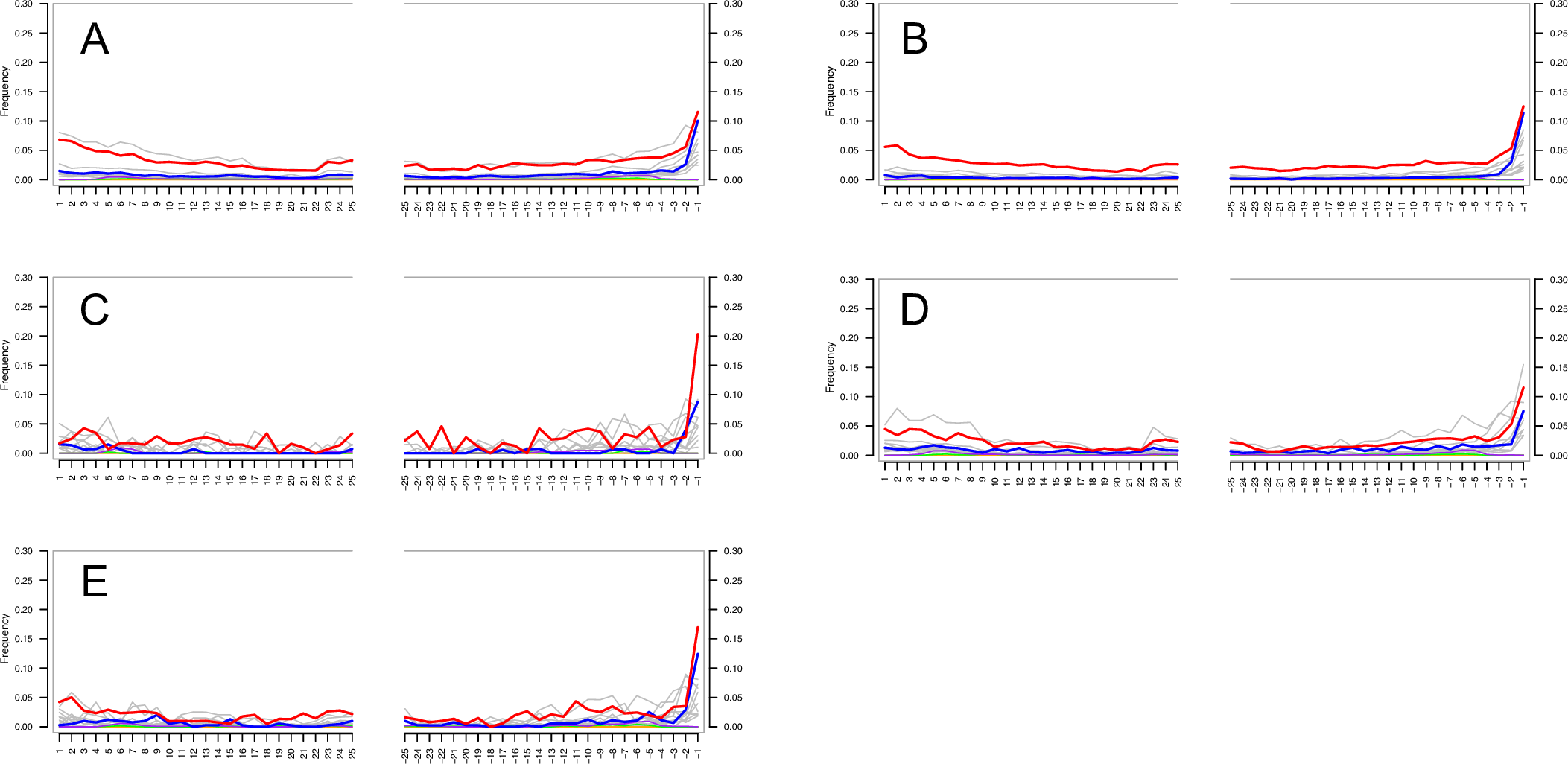

Supplement: S4 Fig — (A) Skin 1; (B) Skin 2; (C) Tumat cartilage; (D) Tumat liver; (E) Tumat muscle. The underlying data for this figure can be found in S1 Data. nt, nucleotide. (TIF) [file pbio.3000166.s004.tif]

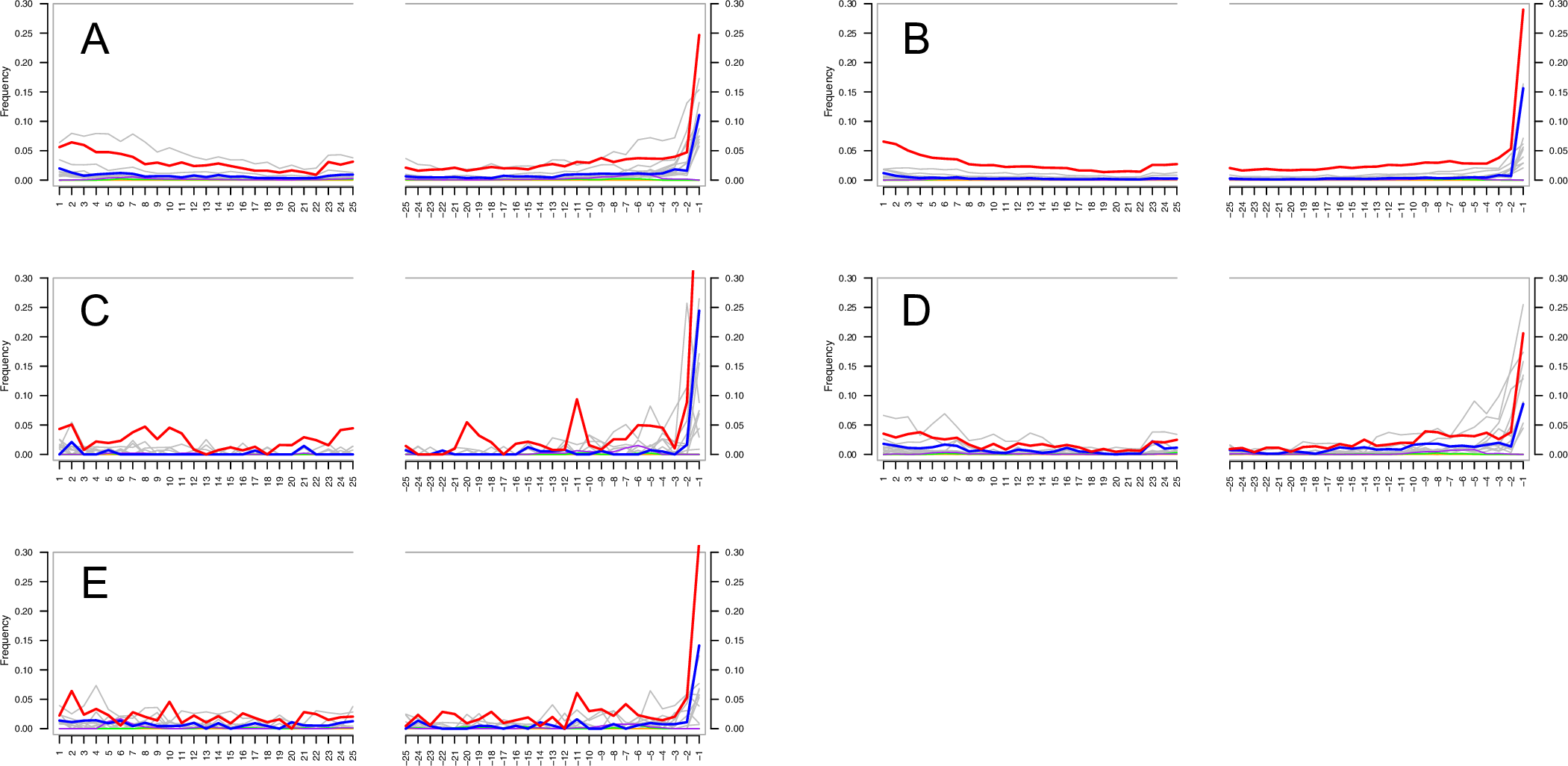

Supplement: S5 Fig — (A) Skin 1; (B) Skin 2; (C) Tumat cartilage; (D) Tumat liver; (E) Tumat muscle. The underlying data for this figure can be found in S1 Data. nt, nucleotide. (TIF) [file pbio.3000166.s005.tif]

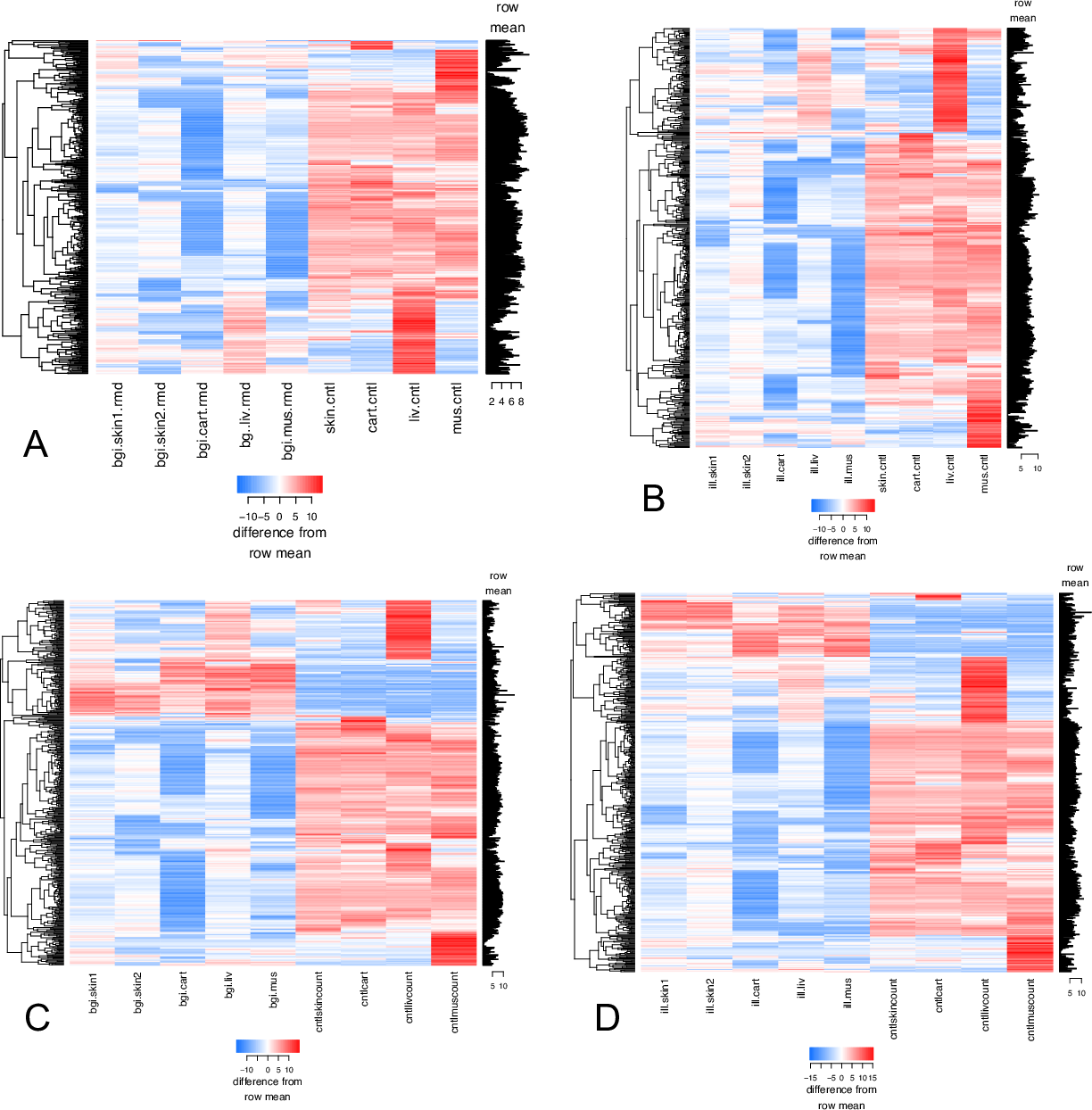

Supplement: S6 Fig — (A) BGISEQ-500 data, de-duplicated; (B) HiSeq-2500 data, de-duplicated; (C) BGISEQ-500 data, duplicates retained; (D) HiSeq-2500 data, duplicates retained. (TIF) [file pbio.3000166.s006.tif]

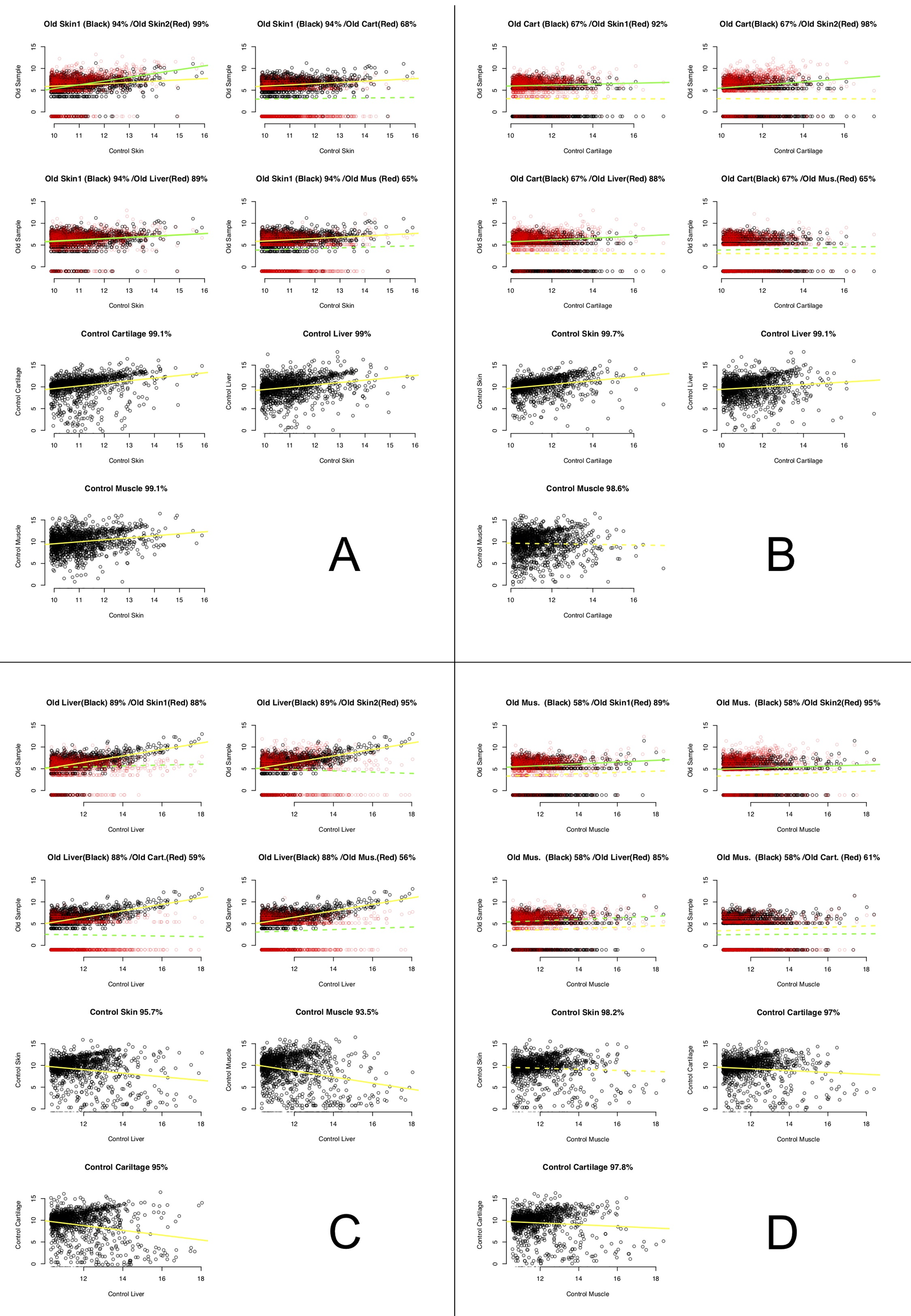

Supplement: S7 Fig — (A) comparison to skin; (B) comparison to cartilage; (C) comparison to liver; (D) comparison to muscle. See legend for Fig 1 for details. (TIF) [file pbio.3000166.s007.tif]

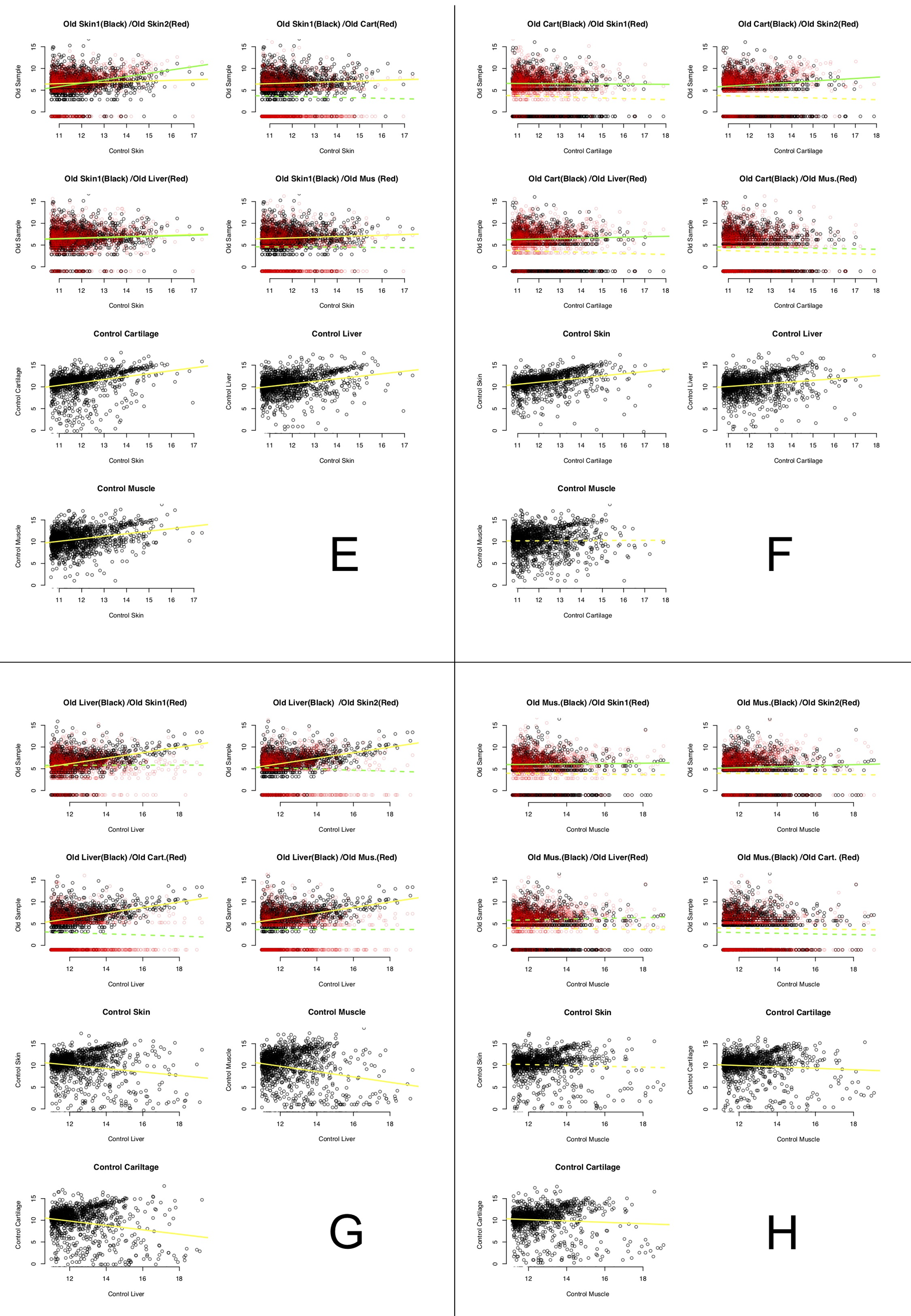

Supplement: S8 Fig — (E) Comparison to skin; (F) comparison to cartilage; (G) comparison to liver; (H) comparison to muscle. See legend for Fig 1 for details. (TIF) [file pbio.3000166.s008.tif]

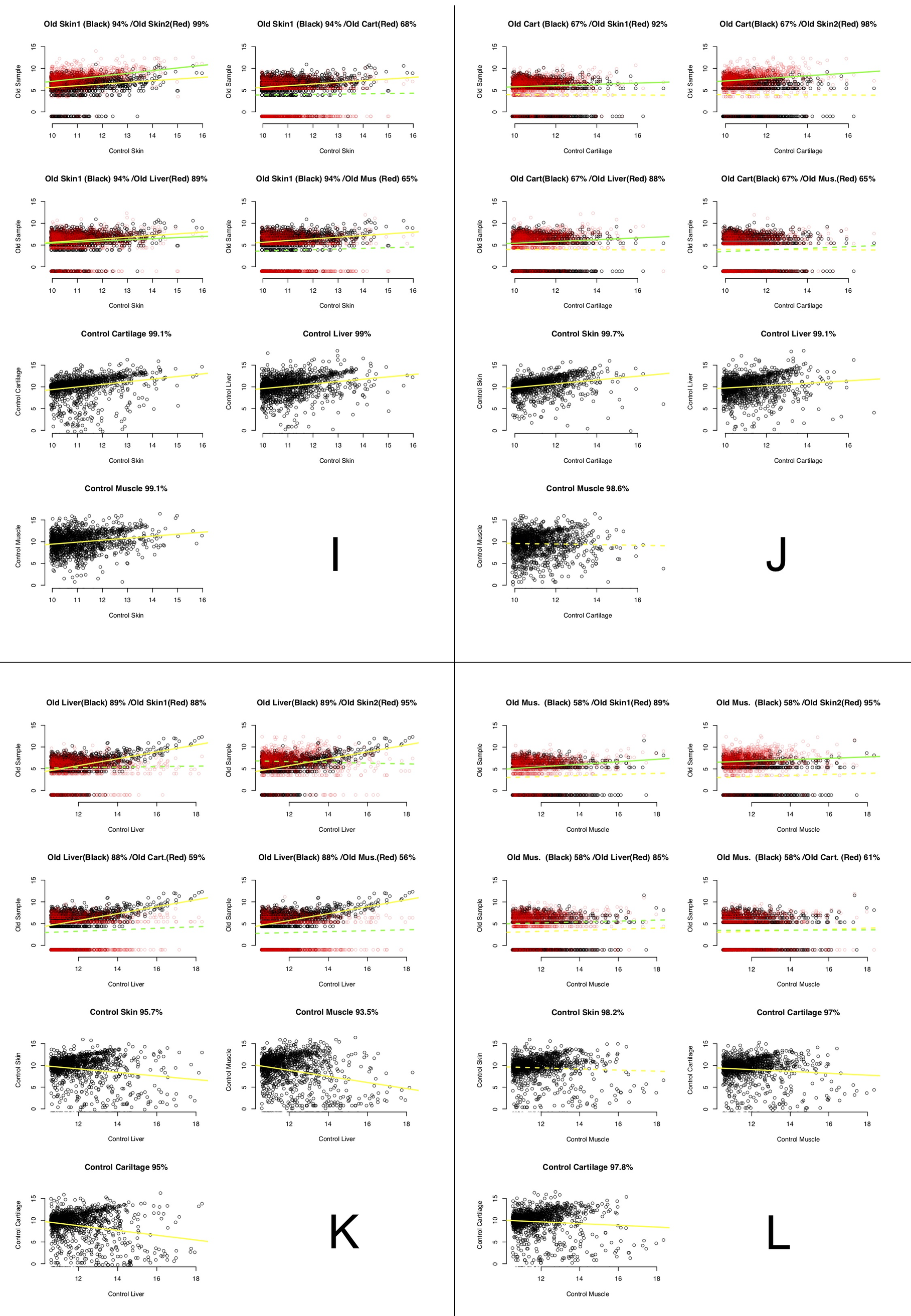

Supplement: S9 Fig — (I) Comparison to skin; (J) comparison to cartilage; (K) comparison to liver; (L) comparison to muscle. See legend for Fig 1 for details. (TIF) [file pbio.3000166.s009.tif]

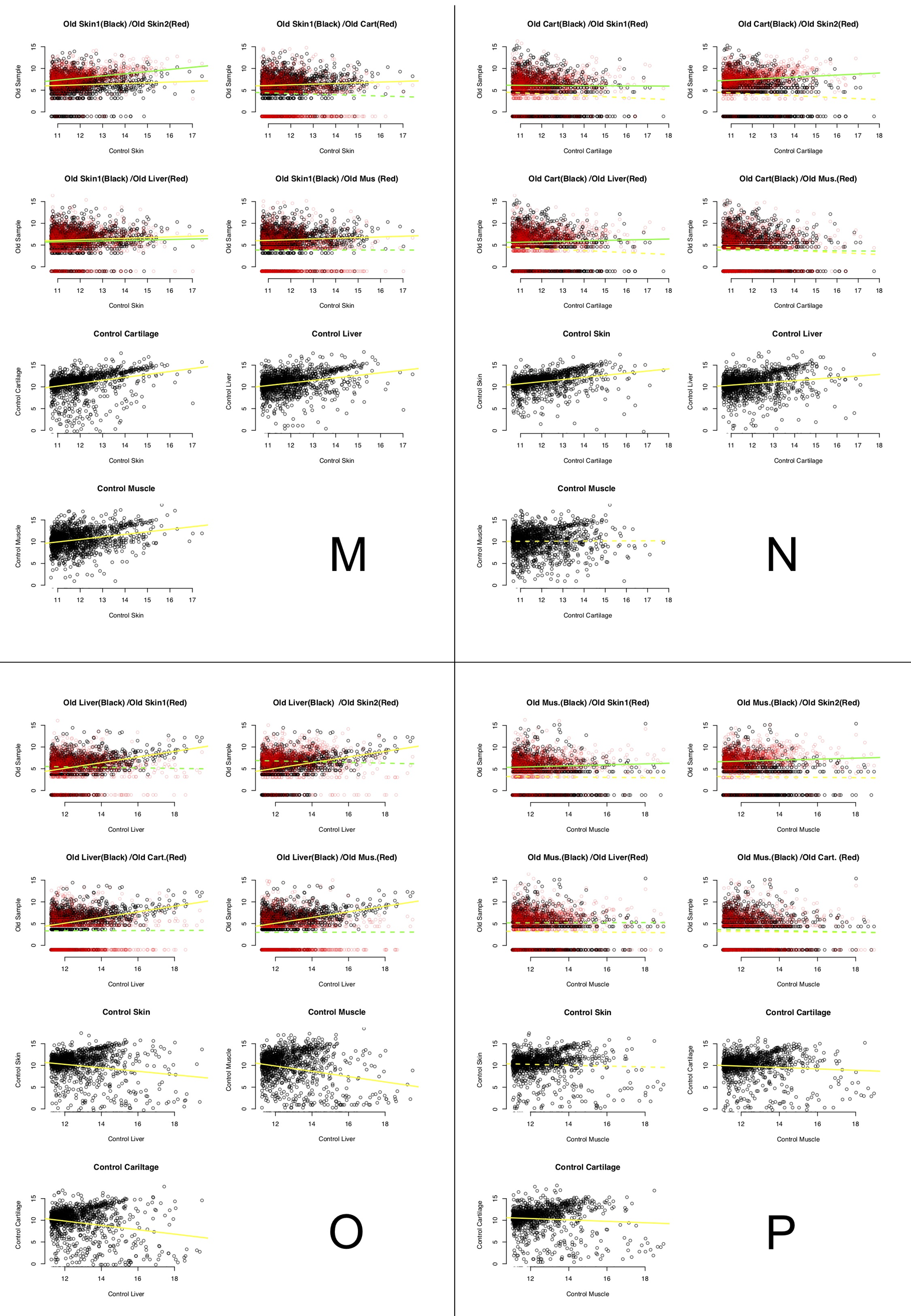

Supplement: S10 Fig — (M) Comparison to skin; (N) comparison to cartilage; (O) comparison to liver; (P) comparison to muscle. See legend for Fig 1 for details. (TIF) [file pbio.3000166.s010.tif]

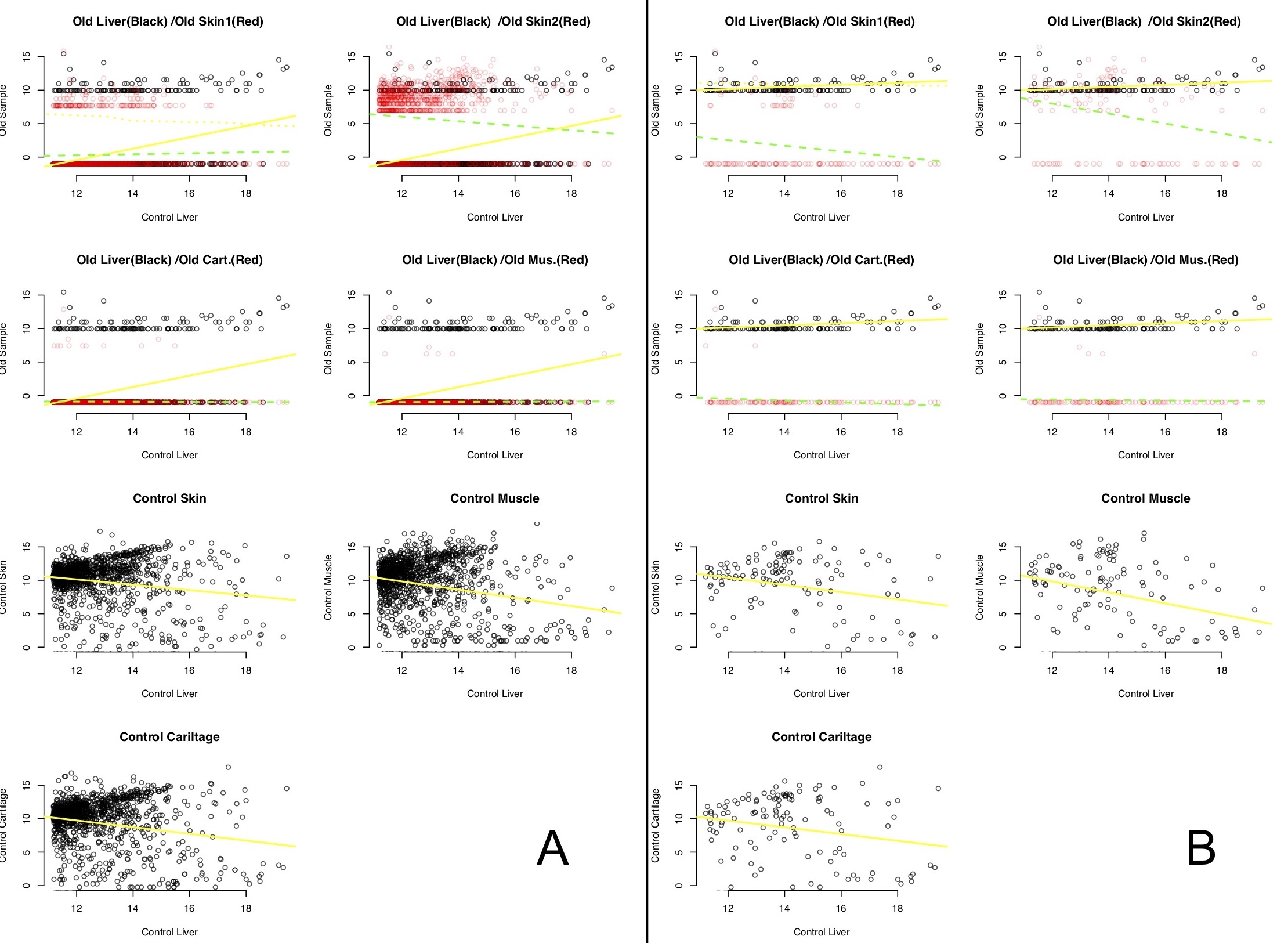

Supplement: S11 Fig — (A) Skin 2; (B) Tumat liver. nt, nucleotide. (TIF) [file pbio.3000166.s011.tif]

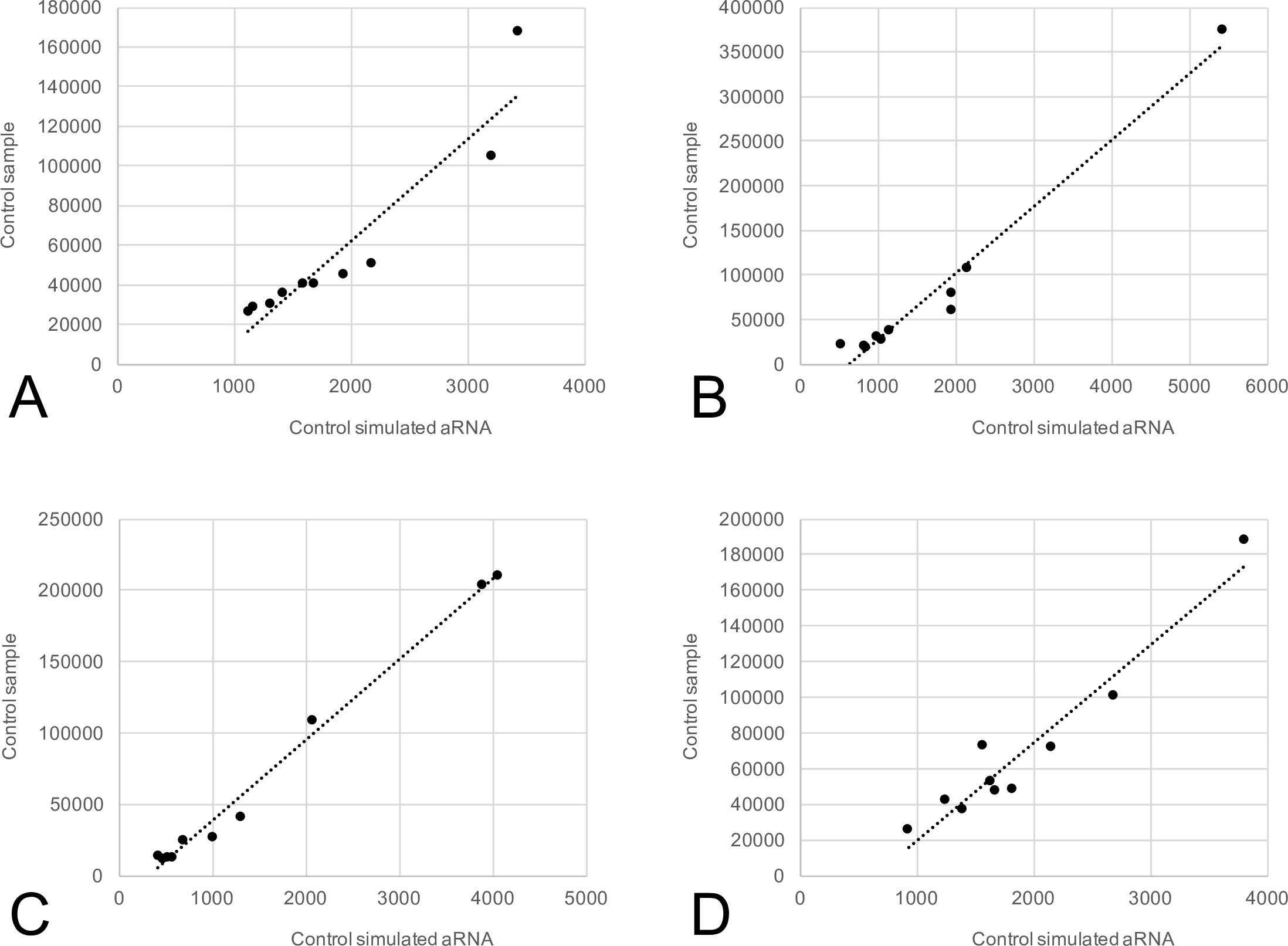

Supplement: S12 Fig — (A) Skin; (B) liver; (C) muscle; (D) cartilage. The underlying data for this figure can be found in S8 Data. (TIF) [file pbio.3000166.s012.tif]

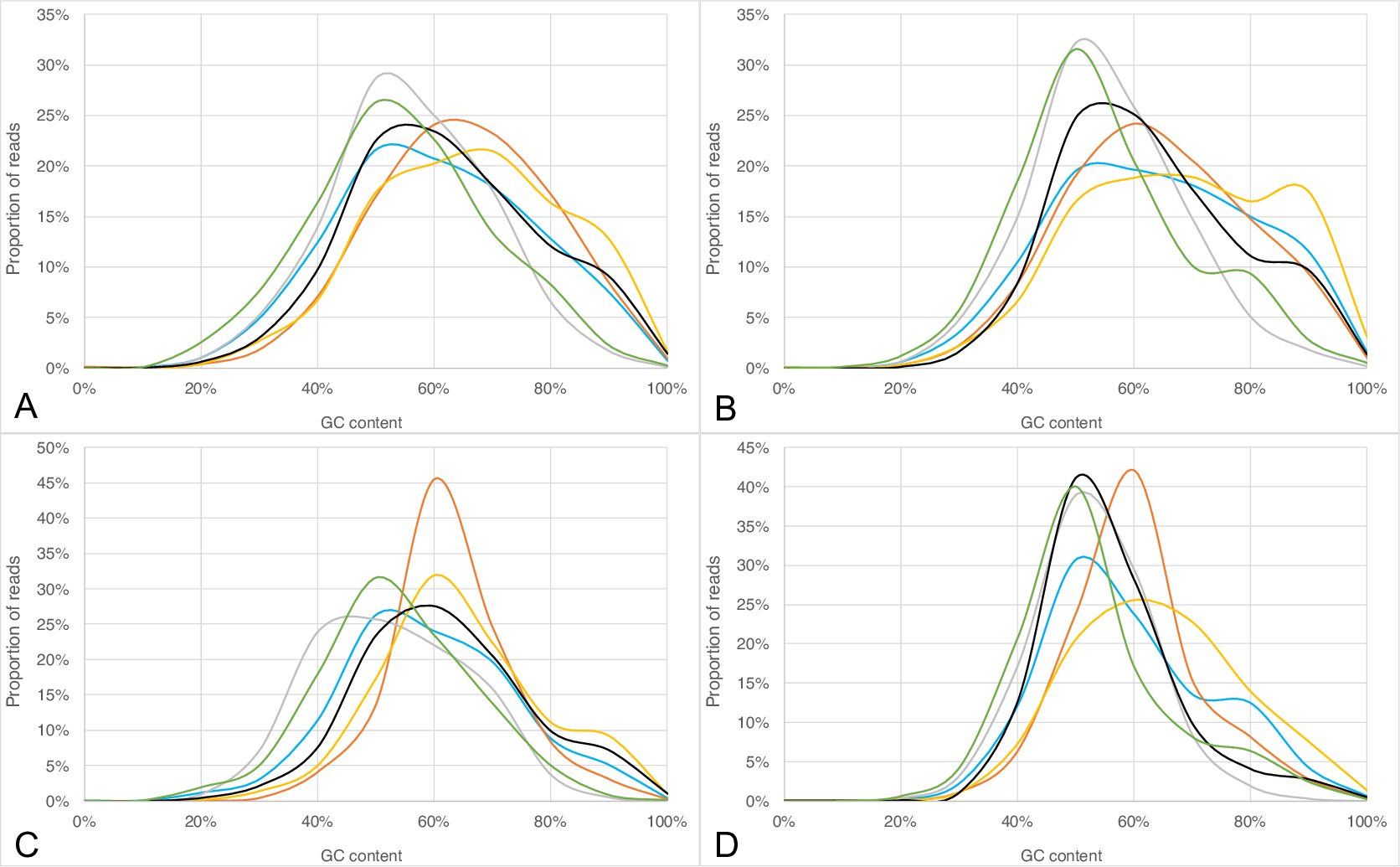

Supplement: S13 Fig — For all panels: blue line, Skin 1; orange line, Skin 2; grey line, Tumat cartilage; yellow line, Tumat liver; black line, Tumat muscle; green line, blank. (A) BGISEQ-500, duplicated removed; (B) HiSeq-2500, duplicated removed; (C) BGISEQ-500, duplicates retained; (D) HiSeq-2500, duplicates retained. (TIF) [file pbio.3000166.s013.tif]

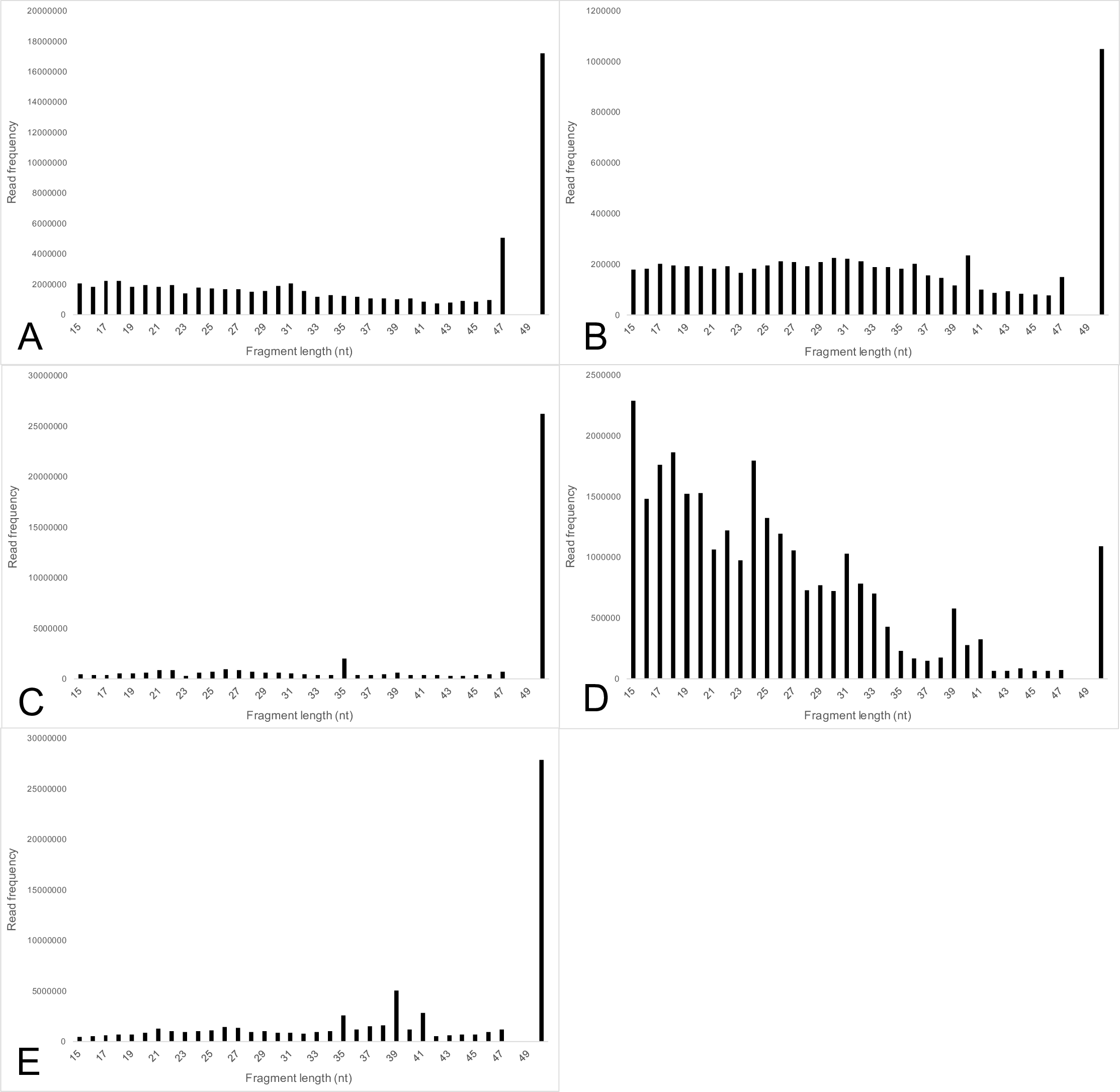

Supplement: S14 Fig — (A) Skin 1; (B) Skin 2; (C) Tumat cartilage; (D) Tumat liver; (E) Tumat muscle. The underlying data for this figure can be found in S9 Data. RNA-seq, RNA sequencing. (TIF) [file pbio.3000166.s014.tif]

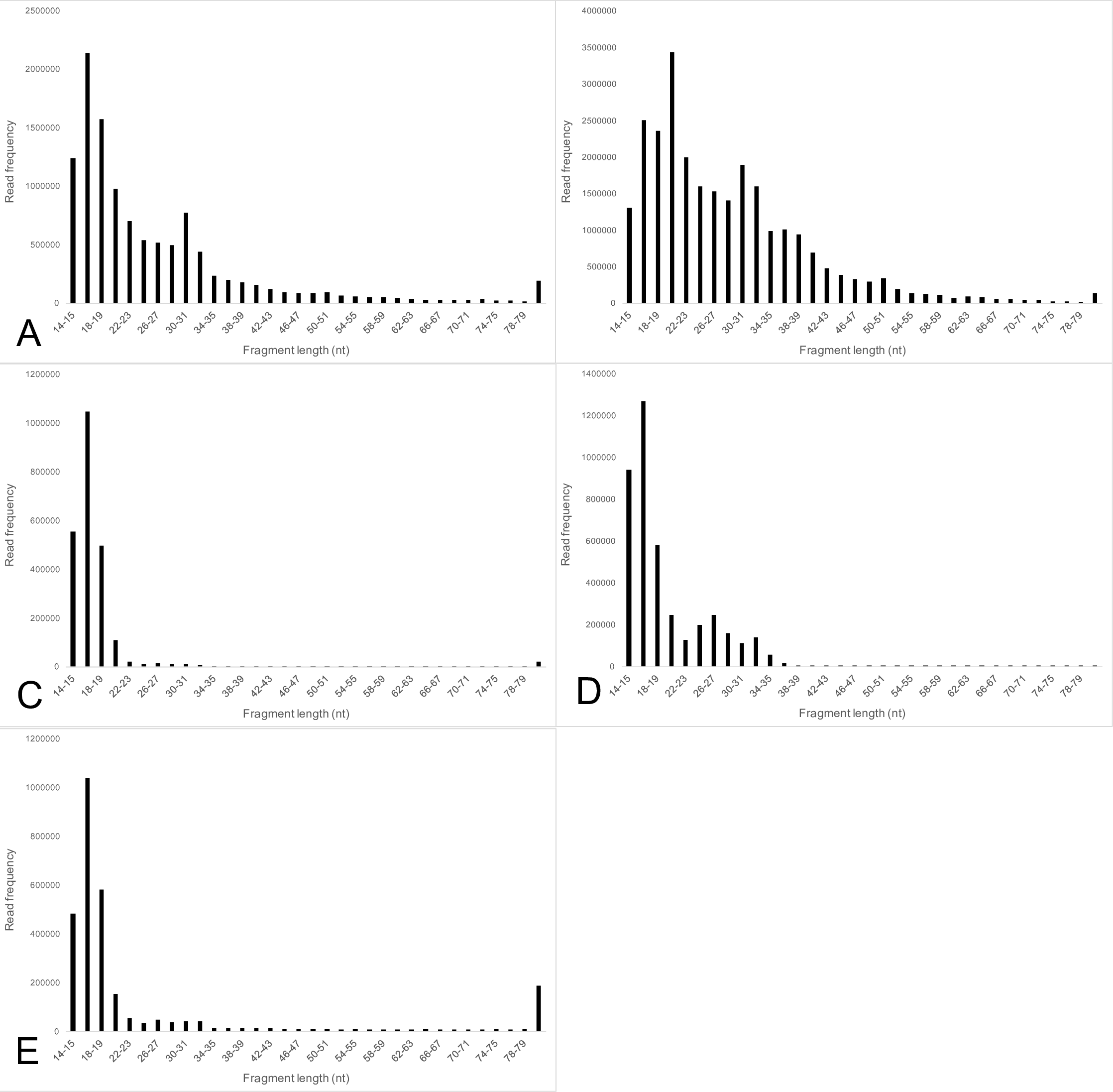

Supplement: S15 Fig — (A) Skin 1; (B) Skin 2; (C) Tumat cartilage; (D) Tumat liver; (E) Tumat muscle. The underlying data for this figure can be found in S10 Data. RNA-seq, RNA sequencing. (TIF) [file pbio.3000166.s015.tif]

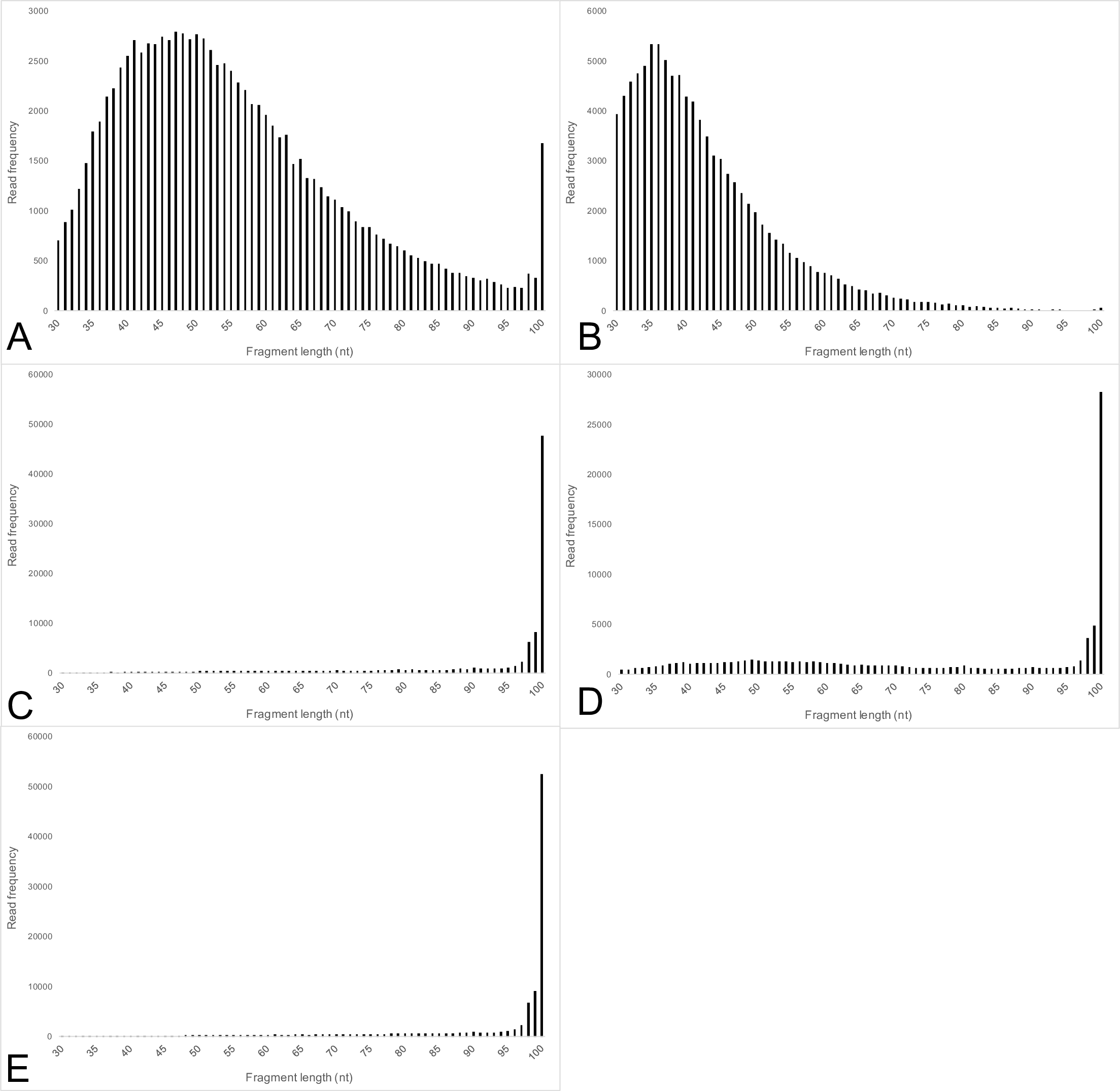

Supplement: S16 Fig — (A) Skin 1; (B) Skin 2; (C) Tumat cartilage; (D) Tumat liver; (E) Tumat muscle. The underlying data for this figure can be found in S11 Data. DNA-seq, DNA sequencing. (TIF) [file pbio.3000166.s016.tif]

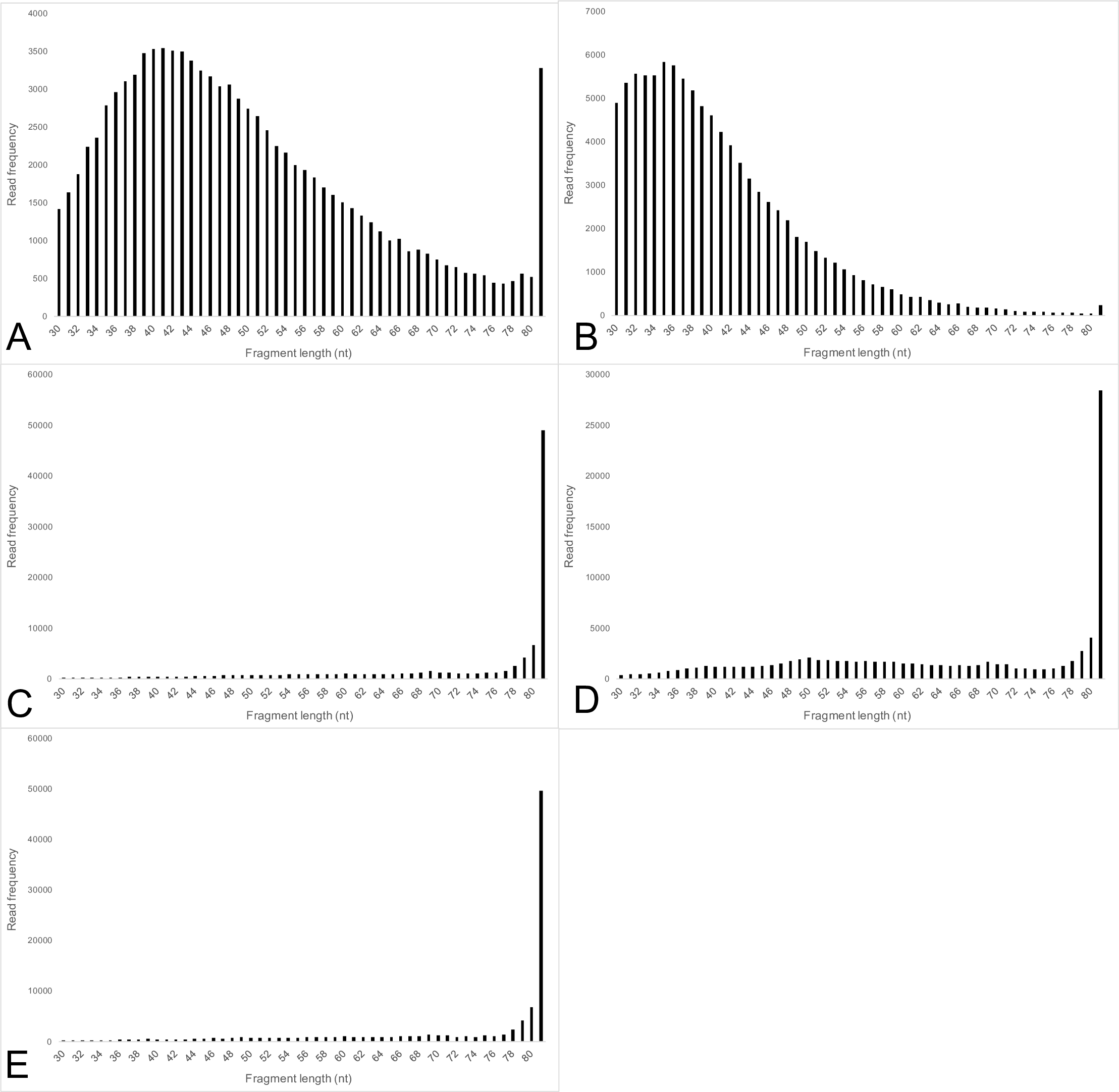

Supplement: S17 Fig — (A) Skin 1; (B) Skin 2; (C) Tumat cartilage; (D) Tumat liver; (E) Tumat muscle. The underlying data for this figure can be found in S12 Data. DNA-seq, DNA sequencing. (TIF) [file pbio.3000166.s017.tif]

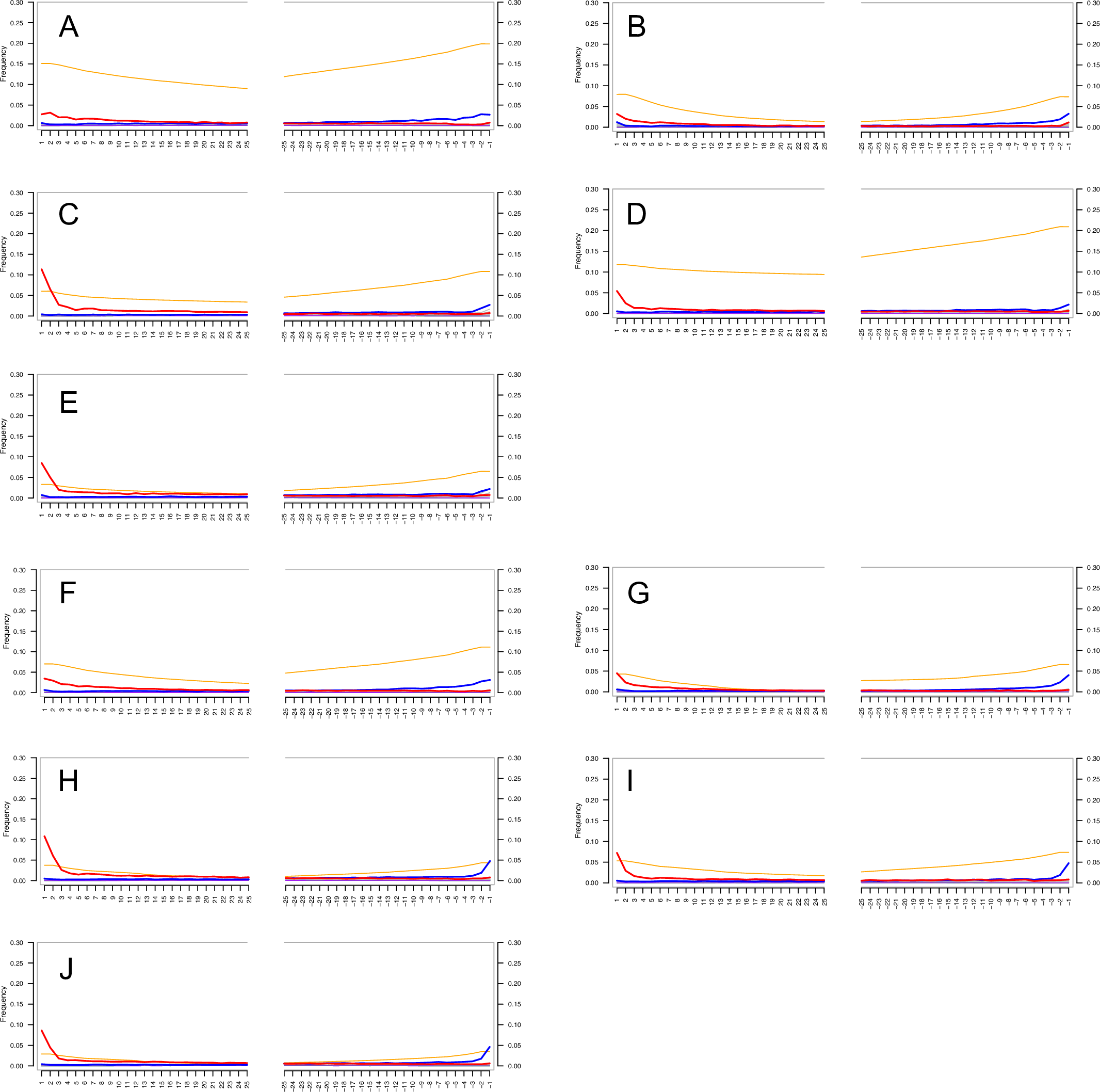

Supplement: S18 Fig — (A–E) Sequenced on the BGISEQ-500 platform. (F–J) Sequenced on the HiSeq-2500 platform. (A) Skin 1; (B) Skin 2; (C) Tumat cartilage; (D) Tumat liver; (E) Tumat muscle. (F) Skin 1; (G) Skin 2; (H) Tumat cartilage; (I) Tumat liver; (J) Tumat muscle. Red lines, frequency of C > U misincorporations; blue lines, frequency of G > A misincorporations; yellow lines, soft-clipped bases from unaligned reads; grey lines, other misincorporations. (TIF) [file pbio.3000166.s018.tif]

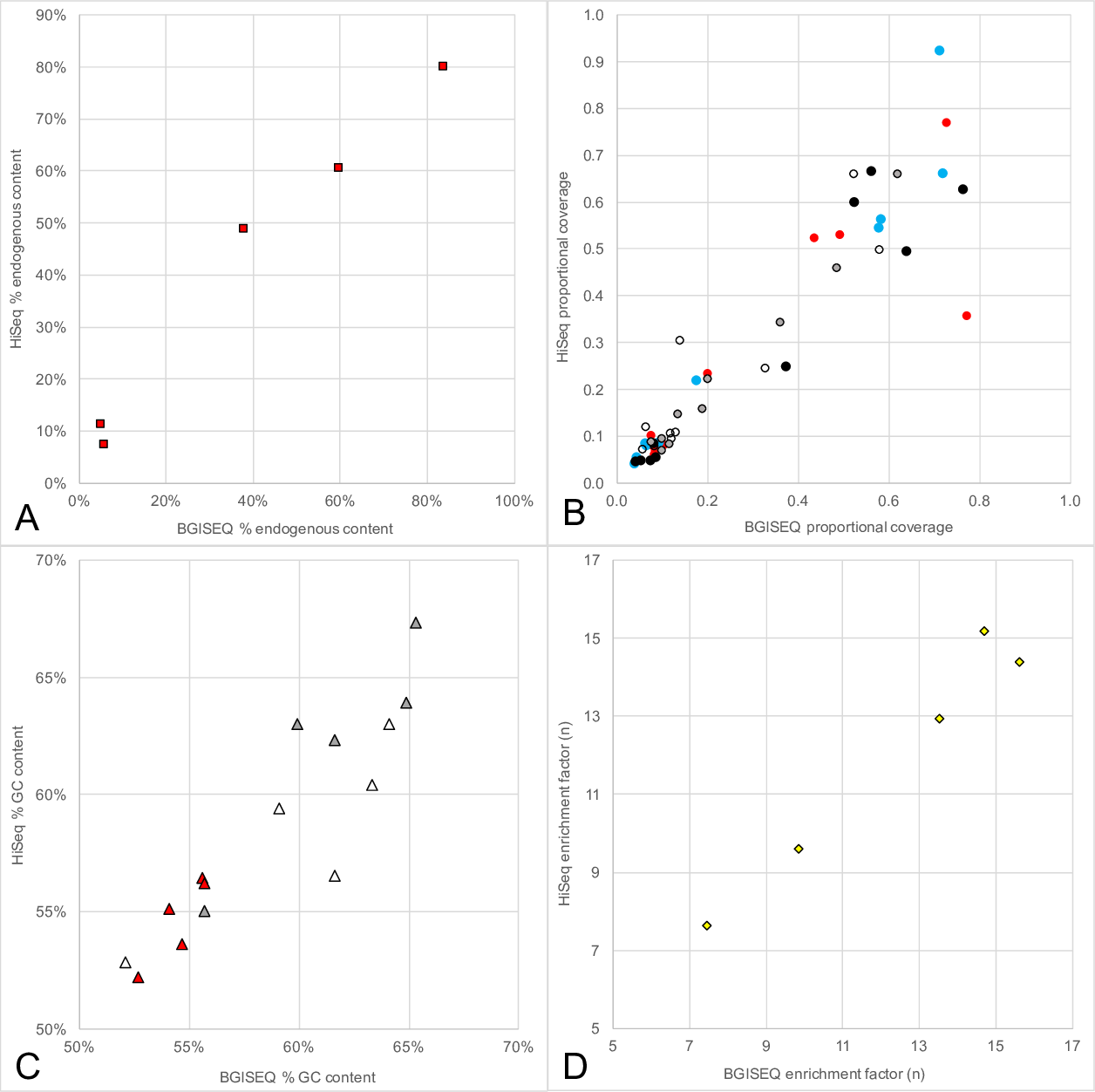

Supplement: S19 Fig — (A) Endogenous content of sequencing reads by tissue (see S4 Table). (B) Regressions of Method 2 between platforms. Red circles, Skin 1; white circles, Tumat cartilage; blue circles, Skin 2; black circles, Tumat liver; grey circles, Tumat muscle. (C) Mean GC content of reads by tissue, depending on duplication. Red triangles, reads mapping to the 95th percentile and above of expression after mapping and de-duplication. White triangles, all mapped reads with de-duplication. Grey triangles, all mapped reads without de-duplication. (D) RNA enrichment factor by tissue type. The underlying data for this figure can be found in S13 Data. (TIF) [file pbio.3000166.s019.tif]

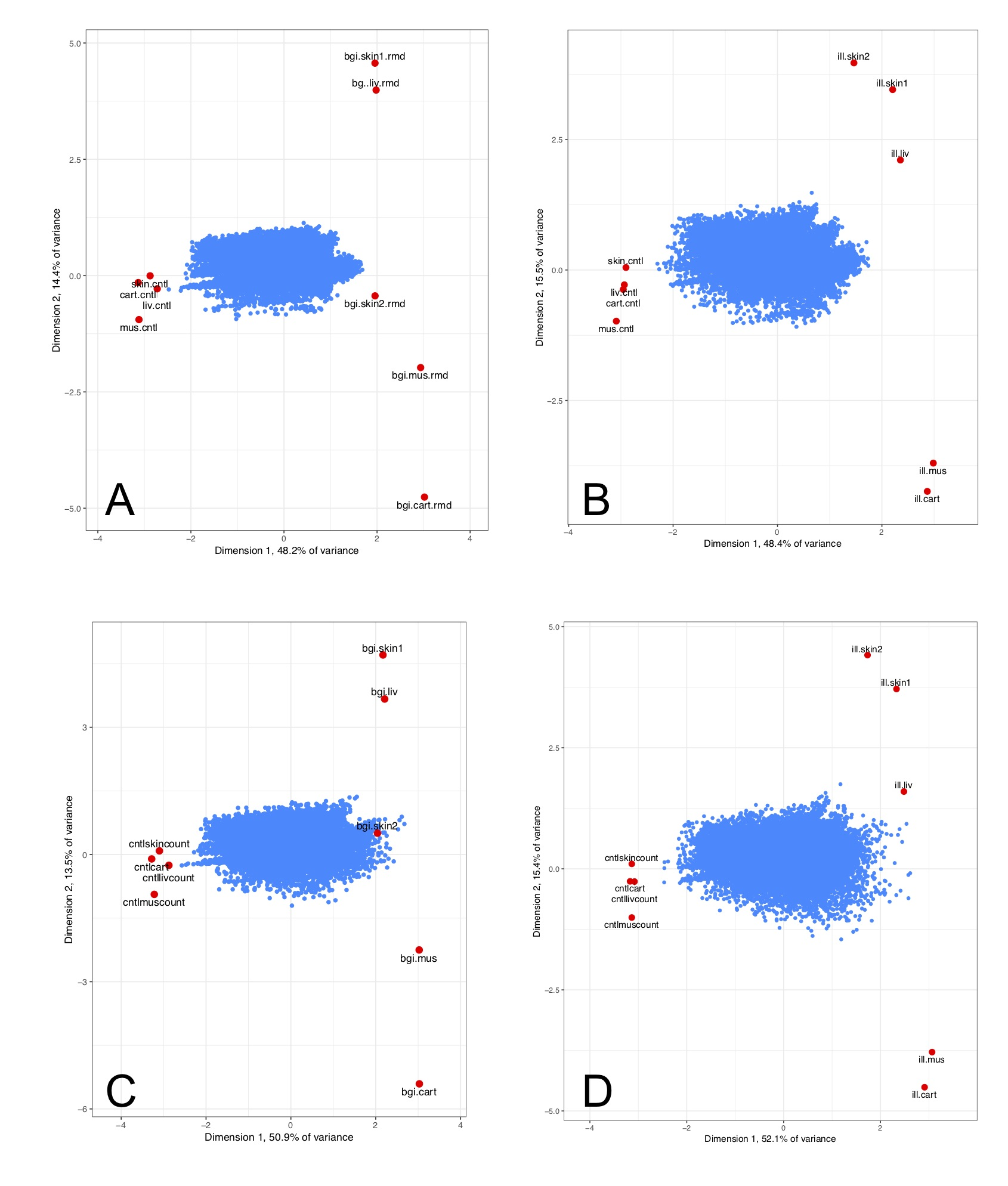

Supplement: S20 Fig — (A) BGISEQ-500 data, de-duplicated; (B) HiSeq-2500 data, de-duplicated; (C) BGISEQ-500 data, duplicates retained; (D) HiSeq-2500 data, duplicates retained. All sample labels ending ‘cntl’ are modern controls. All sample labels ending ‘rmd’ are duplicate-removed samples. Cart, cartilage; ill, Illumina sequencing; Liv, liver; Mus, muscle. (TIF) [file pbio.3000166.s020.tif]

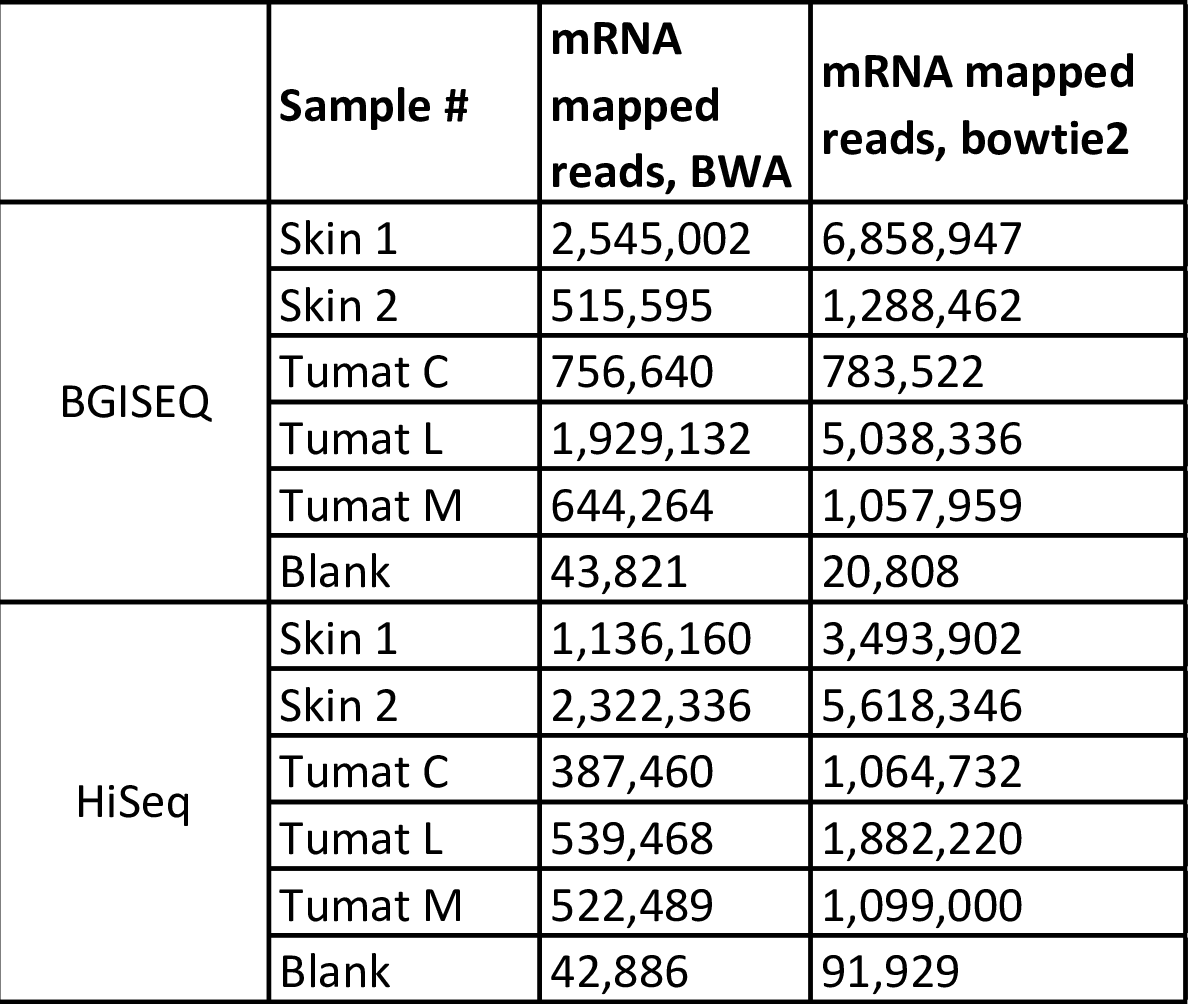

Supplement: S1 Table — (TIF) [file pbio.3000166.s021.tif]

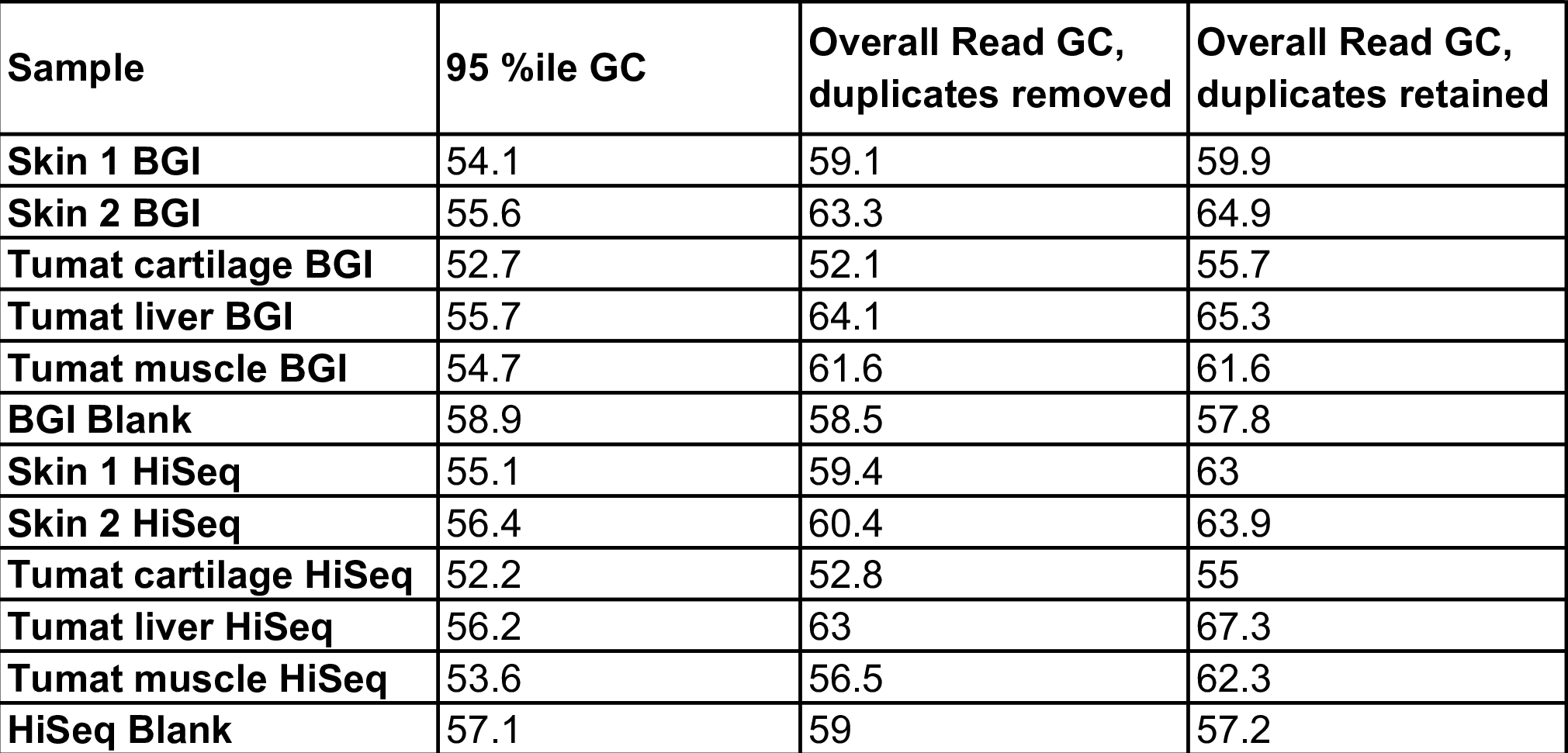

Supplement: S2 Table — (TIF) [file pbio.3000166.s022.tif]

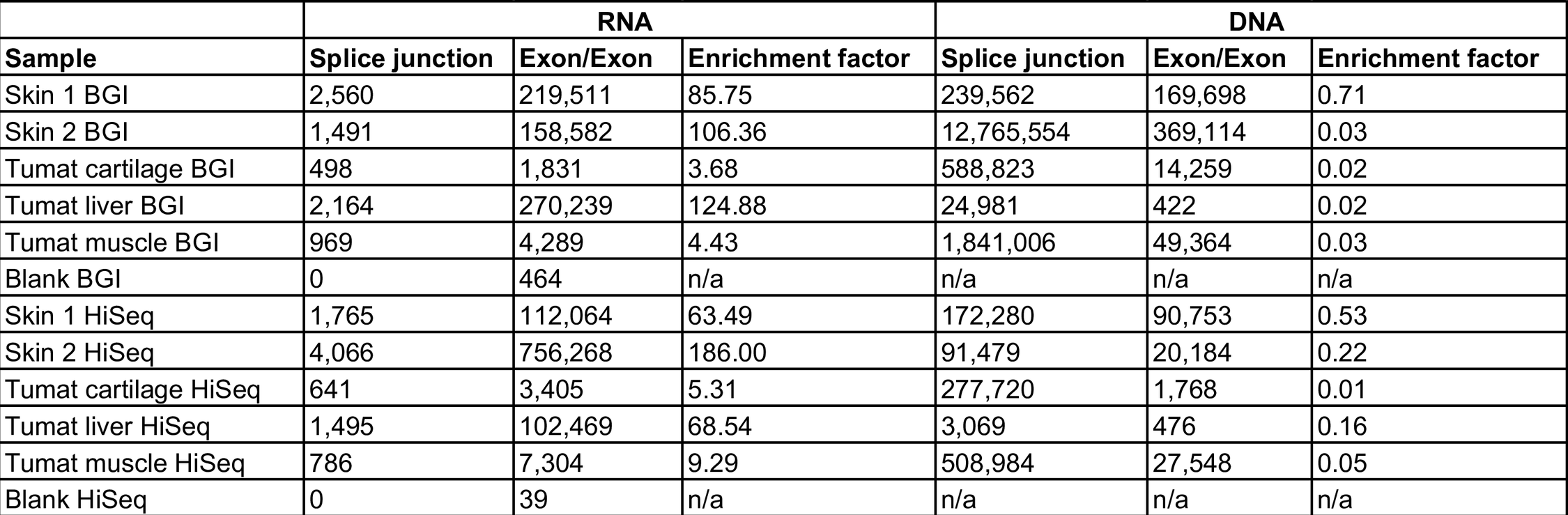

Supplement: S3 Table — Reads mapping over splice junctions and exon-exon junctions were collated for each sample and molecule type, and enrichment factors calculated. In all cases, RNA-seq data show significantly more exon-exon junction coverage than splice junctions, highlighting their authenticity. Conversely, the opposite trend is seen for DNA data. RNA-seq, RNA sequencing. (TIF) [file pbio.3000166.s023.tif]

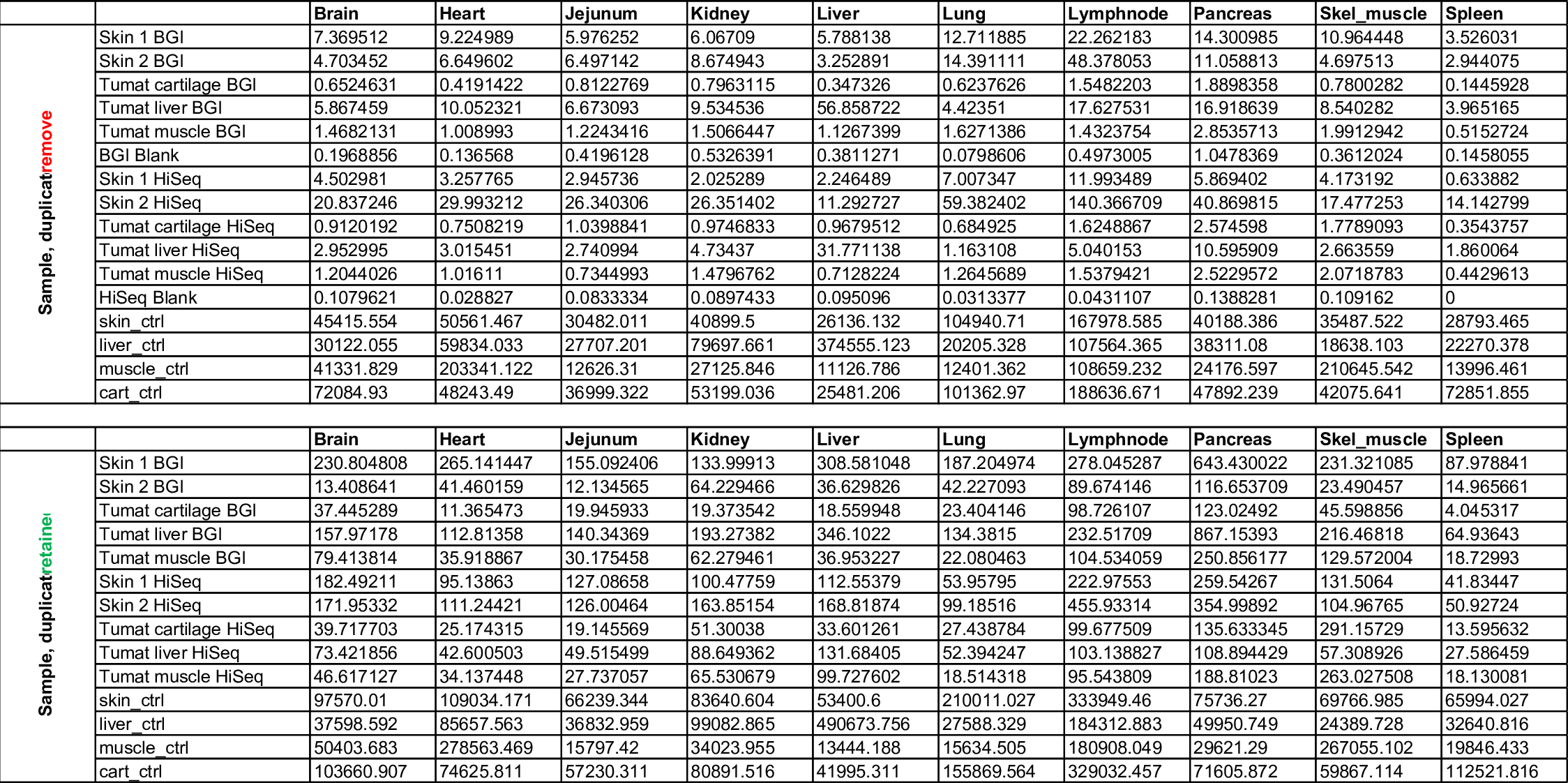

Supplement: S4 Table — Top half, scores following de-duplication. Lower half, scores with duplicate reads retained. NGS, next-generation sequencing. (TIF) [file pbio.3000166.s024.tif]

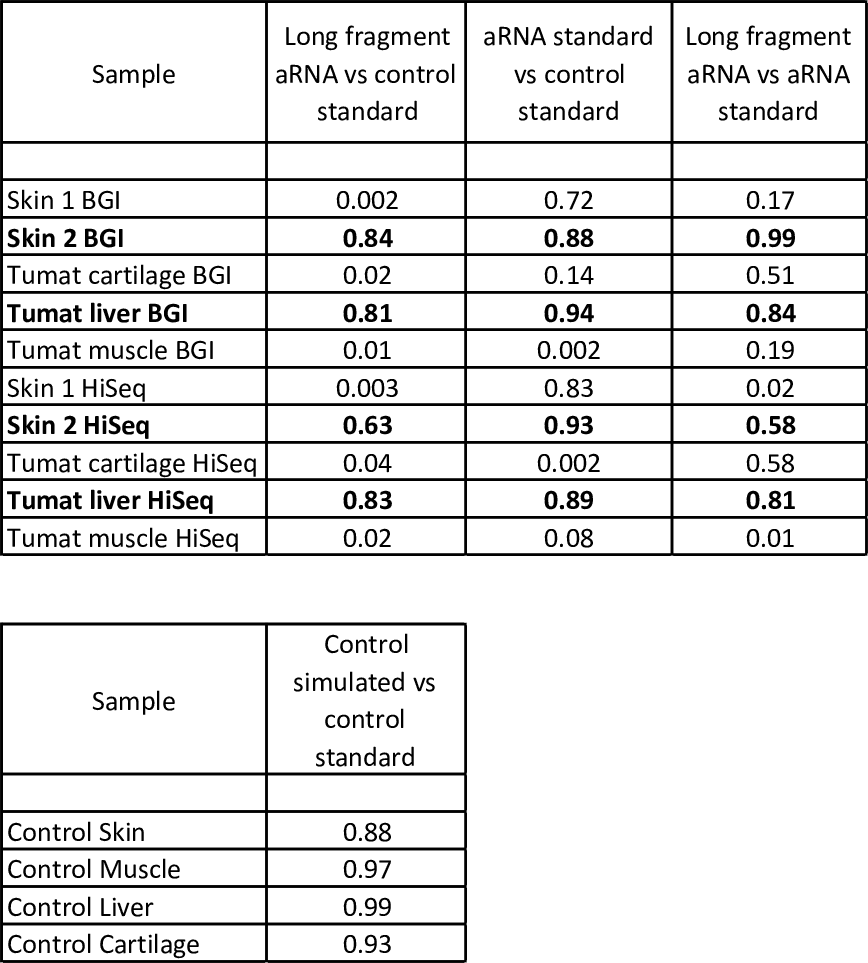

Supplement: S5 Table — The three tissues most similar to their modern counterparts are highlighted in bold. (TIF) [file pbio.3000166.s025.tif]

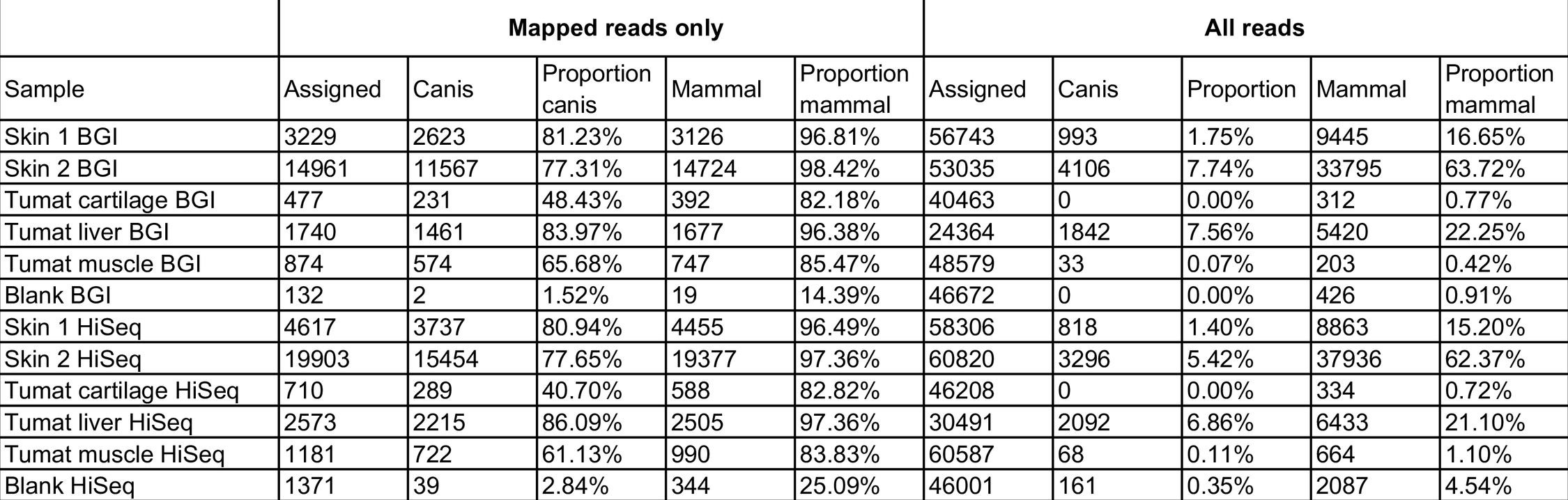

Supplement: S6 Table — (TIF) [file pbio.3000166.s026.tif]

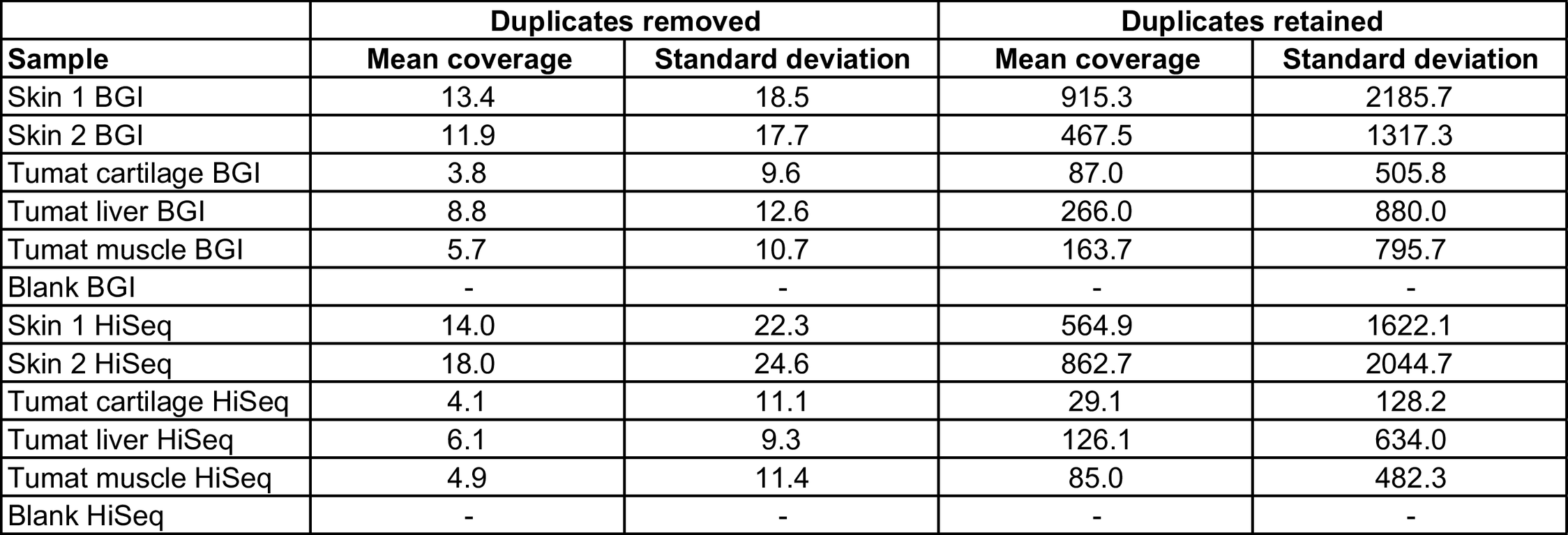

Supplement: S7 Table — Note that the rRNA proportion and overall RNA enrichment factors are significantly less than those of the RNA-seq data. NGS, next-generation sequencing; RNA-seq, RNA sequencing; rRNA, ribosomal RNA. (TIF) [file pbio.3000166.s027.tif]

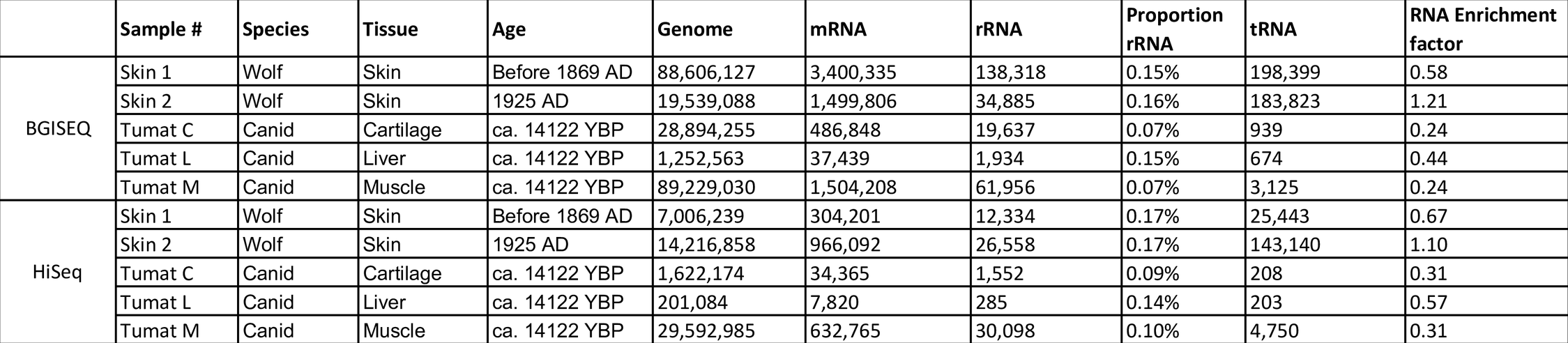

Supplement: S8 Table — rRNA, ribosomal RNA. (TIF) [file pbio.3000166.s028.tif]
