# Supplementary material for: Ancient RNA from Late Pleistocene permafrost and historical canids shows tissue-specific transcriptome survival
Source: PLoS Biol. 2019 Jul 30;17(7):e3000166. doi: 10.1371/journal.pbio.3000166 (PMC6667121; doi:10.1371/journal.pbio.3000166)
Supplement: S1 Text — aRNA, ancient RNA. (DOCX) [file pbio.3000166.s043.docx]

*Metagenomic analysis*

From our BLAST analysis of short reads mapping to the dog transcriptome, we found expected proportions being correctly assigned (see results section). However, we do not usually recommend shotgun metagenomics as a method of establishing endogenous content without secondary verification using something like a phylogenetic intersect analysis [1] because of excessive database over-representation of model organisms affecting proportions of reads which in reality belong to different organisms. Since many model organisms fall into the mammal clade, and dog is not a model organism, we urge caution with these results as they are likely to contain assignment errors.

Further checks against incorrect assignment (i.e. tRNA and rRNA read sharing within samples) showed zero overlap, again allowing us to be confident of our results. We also stress that the copy number of these types of reads should be taken into account as potential sources of error, but these, we believe, are offset by the complexity of the reference transcriptome.

*RNA damage profiles*

RNA Damage profiles, while generally low-level (consistently low with the equivalent DNA damage profiles, S18 Fig), are less consistent with earlier observations of ancient RNA damage which show consistent high-level damage across reads with elevated C>U misincorporations at both ends [2]. However, the equivalent DNA profiles are likely to be a better proxy on which to compare these damage profiles, because the source of the other RNA (in this case, desiccated seeds from southern Egypt) is wildly different in terms of tissue (plant seed endosperm) and burial context (extreme changes in temperature including highs in excess of 40°C). Additionally, these data are some of the only available NGS data derived from aRNA available. The earlier model proposed that RNAs propensity to form secondary structure by self-folding protects mid-sequence cytosines from hydrolytic attack, whereas terminal bases are more exposed and thus more likely to become deaminated. This characteristic is also seen in single-stranded ancient DNA libraries [3], and the different profiles seen in the RNA data suggest that there is little or no DNA contamination in the canine RNA libraries. This being said, we stress that because NGS data derived from aRNA are generally rare, there are very few expectations as to what a ‘typical’ aRNA damage profile would look like. However, an insight might be gained from the damage profiles of canid aRNA after removal of ultrashort fragments, which gave a more obvious symmetrical pattern of C>U transitions at both ends. From this we might conclude that ultrashort fragments mask damage signals due to misalignments or lack of reporting due to minimum seed size or allowed mismatches, resulting in false negatives. Previous transcriptome data from ancient maize kernels shows consistent, low-level damage across the strand, similar to that observed in the historical skin samples shown here [4] although less pronounced than our Pleistocene canid data. We postulate that secondary structure formation, while routinely thermodynamically predictable as *in-situ* transcripts [5], could result in inconsistent or unpredictable (dynamic) de- or re-exposure of cytosine molecules during RNA diagenesis and would thus be, unsurprisingly, a time-dependent diagenetic process. This may be compounded by stochastic fragmentation of RNA molecules, resulting in re-folding or the creation of RNA pseudoknots, the structures of which are less predictable [6]. Further data from a range of sources is needed to crystallise these expectations, and develop models to more accurately predict secondary structure formation in diagenetic assemblages. Considering our general confidence in the inclusion of ultrashort fragments for tissue-specific mapping comparisons, and the loss-of-signal resulting from likely false negatives of damaged ultrashort reads, we suggest that future aRNA datasets are subsampled to reads ≥30 nt for the purposes of damage pattern analysis and verification, and complete datasets then used for expression analysis.

*Sequence duplication in ancient RNA-seq data*

The question of whether to de-duplicate RNA-seq data is much debated [7]; potential issues surrounding type I and type II errors, the effect of greater or fewer PCR cycles, and difficulties in distinguishing a transcript duplicate from a PCR duplicate all contribute to a general uncertainty. In practice, the prevailing opinion appears to be that decisions should be based on individual samples. Some recent developments however suggest that distinguishing duplicate types may be viable under certain circumstances, either computationally [8], or through a molecular-indexing approach [9]. The data presented here however is unique in its age and origin, generated from small starting amounts of RNA and thus prone to type I errors introduced during PCR. On the other hand, random survival of short sequences over long time periods, the effect of secondary structure formation, and other biological processes *may* lend themselves to type II errors. On balance however, we decided that the most parsimonious approach, considering the high numbers of PCR cycles required and the shorter than usual nature of the fragments, would be to treat the de-duplicated dataset as the most informative. To inform this statistically, we decided to measure the mean and standard deviations of a test-set of loci, in this case the 18s rRNA gene defined by the SILVA database [10] (S8 Table). We found that in all cases, the standard deviation was much closer to the mean for de-duplicated samples, suggesting a concentration of PCR duplicates in certain regions, which would serve to skew results in favour of random read mapping. Having removed the duplicates however, we noted that the overall results make more biological sense and so further suggests that in this case at least, and possibly in a majority of aRNA datasets, de-duplicated versions should be considered to be more realistic representations of sequence diversity.

*GC content of ancient RNA data*

We noted that the GC content of reads was slightly higher than those of the transcripts to which they were mapped, and further increased when accounting for duplicate reads (S13 Fig). We believe that a combination of excess duplicates arising from the high number of PCR cycles necessary for NGS library construction (as opposed to ‘true’ transcript duplicates), the trend of transcribed regions of mammalian genomes being generally GC-rich [11] and the greater survivability of GC-rich fragments of ancient biomolecules, is responsible for this observation. We therefore suggest that in this instance, the de-duplicated datasets are more likely to be accurate approximations of the ‘true’ transcripts from these samples. We observed in both our statistical methods applied to read coverage that the de-duplicated ancient datasets showed significantly greater similarity to control dataset, regardless of de-duplication of the controls. This is likely due to the fact that duplicates in the control samples were significantly lower, and where present, representative of actual *in vivo* transcript expression as opposed to PCR biases. In all cases, the GC content was elevated in datasets with duplicates retained; however the BGISEQ-500data showed that this trend was slightly less pronounced, despite library protocols being identical apart from the platform-specific adapters used and the sequencing platform itself. It is possible that potential biases arising from the use of certain polymerases used during library construction or sequencing; however, since the library construction enzymes were identical for all samples, and the differences between platforms are slight, we do not believe that potential biases are of concern. It is possible that polymerases used for circularisation (a.k.a DNA Nanoball formation, BGI) or bridge amplification steps (Illumina) may be different, but the negligible differences between the observed GC values makes this unlikely to be a problem.

*Comparison of Illumina HiSeq-2500 and BGISEQ-500 sequencing platforms*

Following the comparison of Illumina and BGISEQ-500 platforms on aDNA, which showed little difference in standard quantitative metrics between them [12], we decided to use both platforms in this study to a) compare the two when using aRNA instead of aDNA, and b) treat one as a technical replicate for proof-of-concept purposes. Overall, we found very little difference between platforms in terms of sequence quality, GC bias and overall analytical outcomes between HiSeq-2500 and BGISEQ-500 platforms (S19 Fig), in keeping with previous comparisons of these platforms using DNA data [12]. The most noticeable difference was the fragment size distribution after adapter removal; we noted that the HiSeq-2500 gives a higher proportion of small fragments than BGISEQ-500 (S14 Fig, S15 Fig), likely due to preferential clustering of small fragments on Illumina flowcells as noted previously by the manufacturers. Crucially however, we noted that comparisons following biologically meaningful analyses retained strong correlation. In particular, we found that the calculated endogenous content and RNA enrichment factors were almost identical for both following linear regression (r^2^ = 0.98 and 0.96 respectively, S19 Fig. panels A and D, Table 2). The relationships between control and ancient tissues using Method 1 were also very similar, with BGISEQ-500 slightly outperforming HiSeq-2500 explaining 20% of the variance (compared with 16% explained with HiSeq). The standardised individual gene expression metrics and similarity between individual samples were likewise similar between the two platforms (S20 Fig).

In terms of GC content of mapped reads, we did note slightly higher discrepancies between the two sequencing platforms: Of the reads mapping to transcripts in the top 95^th^ percentile of coverage depth, we found lesser but significant correlation (r^2^ = 0.78), and GC of all reads following duplicate removal at a similar correlation (r^2^ = 0.75). A better correlation was observed in GC content of all reads prior to duplicate removal (r^2^ = 0.85), suggesting that both platforms gave data slightly biased towards GC retention. This is not to say the platforms themselves exhibit bias, but is more likely to be a function of long-term preservation favouring GC-rich molecules as previously noted [13]. We did however notice this bias to be slightly increased overall in the BGISEQ-500 platform (S13 Fig, S13 Fig. panel C), although this effect appears to be negligible in downstream analysis. We also note that the recommended library input requirements into pre-sequencing treatment are higher for BGISEQ, which is not an insignificant point considering the generally much smaller quantities of DNA / RNA available to palaeogenomic study.

In terms of read duplication, we found that the BGISEQ-500 platform slightly outperformed HiSeq-2500 by having a lower proportion of duplicated reads in all samples except Tumat liver. However, we noted that while higher, duplication levels from the HiSeq-2500 platform were more consistent with each other, varying between samples by 6.2% versus the BGISEQ-500 platform at 20.1%. Since our primary analyses and conclusions are based on de-duplicated reads, this result makes no difference to our conclusions. For the analysis of reads straddling splice or exon-exon junctions, we again found little difference between platforms, although again the BGISEQ-500 slightly outperformed HiSeq-2500 in identifying a higher proportion of exon-exon junction reads compared to splice junction reads in the RNA data. The relative proportions of the same analysis performed on the previously-sequenced DNA data showed negligible differences between the two platforms (S3 Table). While both platforms are broadly similar in terms of all metrics of the data returned, we suggest that researchers, particularly those working with low-yield ancient samples, should consider issues such as data output, cost-per-read, and library input mass, to decide on the best fit for individual projects.

1. Smith O, Momber G, Bates R, Garwood P, Fitch S, Pallen M, et al. Sedimentary DNA from a submerged site reveals wheat in the British Isles 8000 years ago. Science. 2015;347(6225):998-1001. doi: 10.1126/science.1261278.

2. Smith O, Clapham A, Rose P, Liu Y, Wang J, Allaby RG. A complete ancient RNA genome: identification, reconstruction and evolutionary history of archaeological Barley Stripe Mosaic Virus. Sci Rep. 2014;4(4003). doi: 10.1038/srep04003.

3. Gansauge M-T, Meyer M. Single-stranded DNA library preparation for the sequencing of ancient or damaged DNA. Nat Protoc. 2013;8:737. doi: 10.1038/nprot.2013.038.

4. Fordyce SL, Avila-Arcos MC, Rasmussen M, Cappellini E, Romero-Navarro JA, Wales N, et al. Deep Sequencing of RNA from Ancient Maize Kernels. PLoS One. 2013;8(1):e50961. doi: 10.1371/journal.pone.0050961.

5. Eddy SR. Computational Analysis of Conserved RNA Secondary Structure in Transcriptomes and Genomes. Annual Review of Biophysics. 2014;43(1):433-56. doi: 10.1146/annurev-biophys-051013-022950.

6. Lyngsø RB, Pedersen CNS. RNA Pseudoknot Prediction in Energy-Based Models. J Comput Biol. 2000;7(3-4):409-27. doi: 10.1089/106652700750050862.

7. Parekh S, Ziegenhain C, Vieth B, Enard W, Hellmann I. The impact of amplification on differential expression analyses by RNA-seq. Sci Rep. 2016;6:25533. doi: 10.1038/srep25533.

8. Bansal V. A computational method for estimating the PCR duplication rate in DNA and RNA-seq experiments. BMC Bioinformatics. 2017;18(3):43. doi: 10.1186/s12859-017-1471-9.

9. Klepikova AV, Kasianov AS, Chesnokov MS, Lazarevich NL, Penin AA, Logacheva M. Effect of method of deduplication on estimation of differential gene expression using RNA-seq. PeerJ. 2017;5:e3091. doi: 10.7717/peerj.3091. PubMed PMID: PMC5357343.

10. Quast C, Pruesse E, Gerken J, Peplies J, Yarza P, Yilmaz P, et al. The SILVA ribosomal RNA gene database project: improved data processing and web-based tools. Nucleic Acids Res. 2012;41(D1):D590-D6. doi: 10.1093/nar/gks1219.

11. Zhang L, Kasif S, Cantor CR, Broude NE. GC/AT-content spikes as genomic punctuation marks. Proc Natl Acad Sci U S A. 2004;101(48):16855-60. doi: 10.1073/pnas.0407821101.

12. Mak SST, Gopalakrishnan S, Carøe C, Geng C, Liu S, Sinding M-HS, et al. Comparative performance of the BGISEQ-500 vs Illumina HiSeq2500 sequencing platforms for palaeogenomic sequencing. GigaScience. 2017;6(8):1-13. doi: 10.1093/gigascience/gix049. PubMed PMID: PMC5570000.

13. Briggs AW, Stenzel U, Johnson PLF, Green RE, Kelso J, Prüfer K, et al. Patterns of damage in genomic DNA sequences from a Neandertal. Proc Natl Acad Sci U S A. 2007;104(37):14616-21. doi: 10.1073/pnas.0704665104. PubMed PMID: PMC1976210.
