# Supplementary material for: Ancient RNA from Late Pleistocene permafrost and historical canids shows tissue-specific transcriptome survival
Source: PLoS Biol. 2019 Jul 30;17(7):e3000166. doi: 10.1371/journal.pbio.3000166 (PMC6667121; doi:10.1371/journal.pbio.3000166)
Supplement: S1 Data — (ZIP) [file pbio.3000166.s029.zip › S3_Fig.zip!/S3_Fig/results_Tumat_Cart_transcriptome_srt_rmdup/Length_plot.pdf]

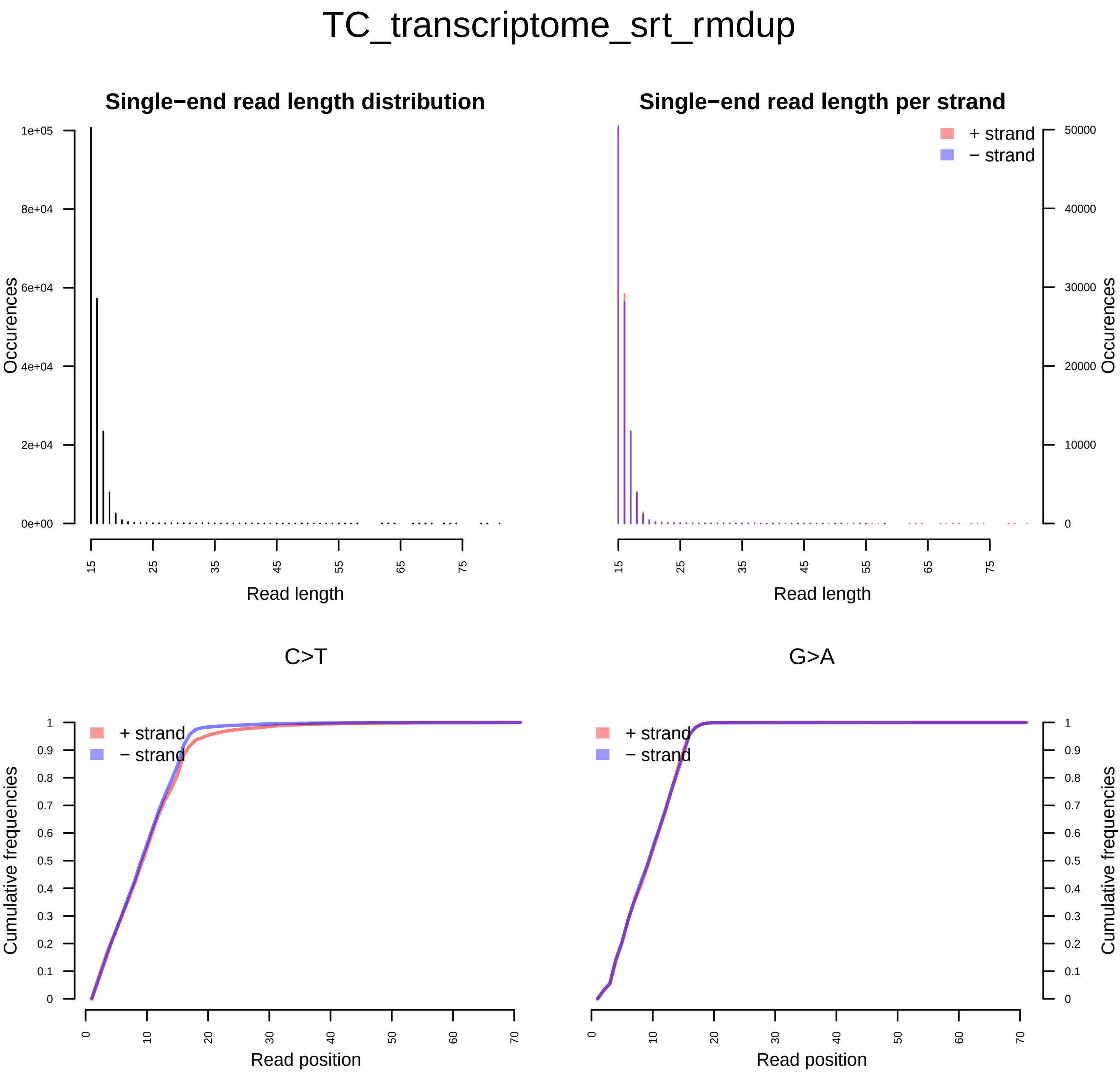

TC_transcriptome_srt_rmdup
Single−end read length distribution
Occurences
Read length
1e+05
8e+04
6e+04
4e+04
2e+04
0e+00
15
25
35
45
55
65
75
Single−end read length per strand
+ strand
− strand
50000
40000
30000
20000
10000
0
C>T
Cumulative frequencies
Read position
G>A
1
0.9
0.8
0.7
0.6
0.5
0.4
0.3
0.2
0.1
0
10
20
30
40
50
60
70
