# Supplementary material for: Ancient RNA from Late Pleistocene permafrost and historical canids shows tissue-specific transcriptome survival
Source: PLoS Biol. 2019 Jul 30;17(7):e3000166. doi: 10.1371/journal.pbio.3000166 (PMC6667121; doi:10.1371/journal.pbio.3000166)

1_transcriptome_srt

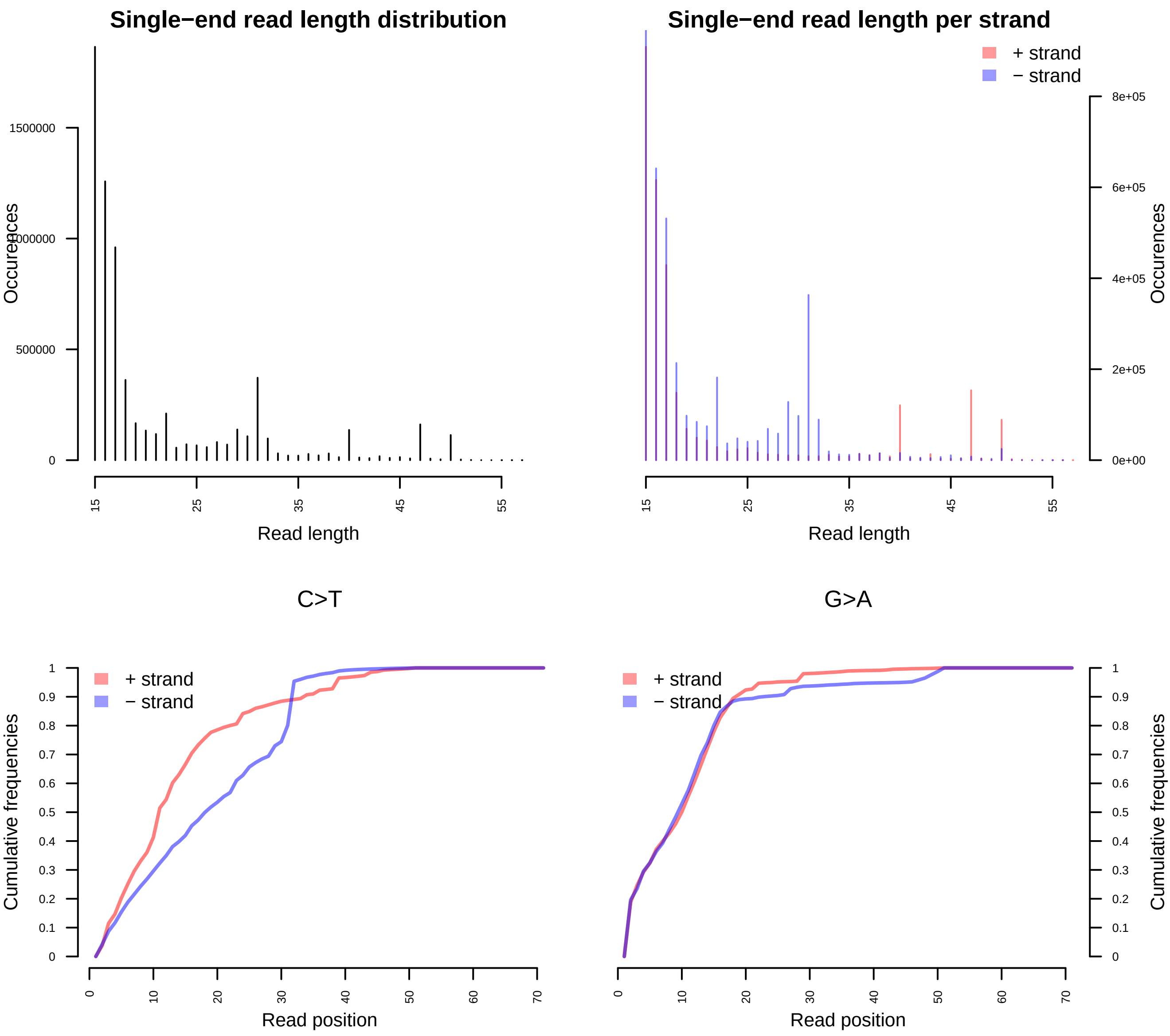

Supplement: S1 Data — (ZIP) [file pbio.3000166.s029.zip › S2_Fig.zip!/S2_Fig/results_Skin1_transcriptome_srt/Length_plot.pdf]
