# Supplementary material for: Ancient RNA from Late Pleistocene permafrost and historical canids shows tissue-specific transcriptome survival
Source: PLoS Biol. 2019 Jul 30;17(7):e3000166. doi: 10.1371/journal.pbio.3000166 (PMC6667121; doi:10.1371/journal.pbio.3000166)
Supplement: S1 Data — (ZIP) [file pbio.3000166.s029.zip › S2_Fig.zip!/S2_Fig/results_Skin1_transcriptome_srt_rmdup/Length_plot.pdf]

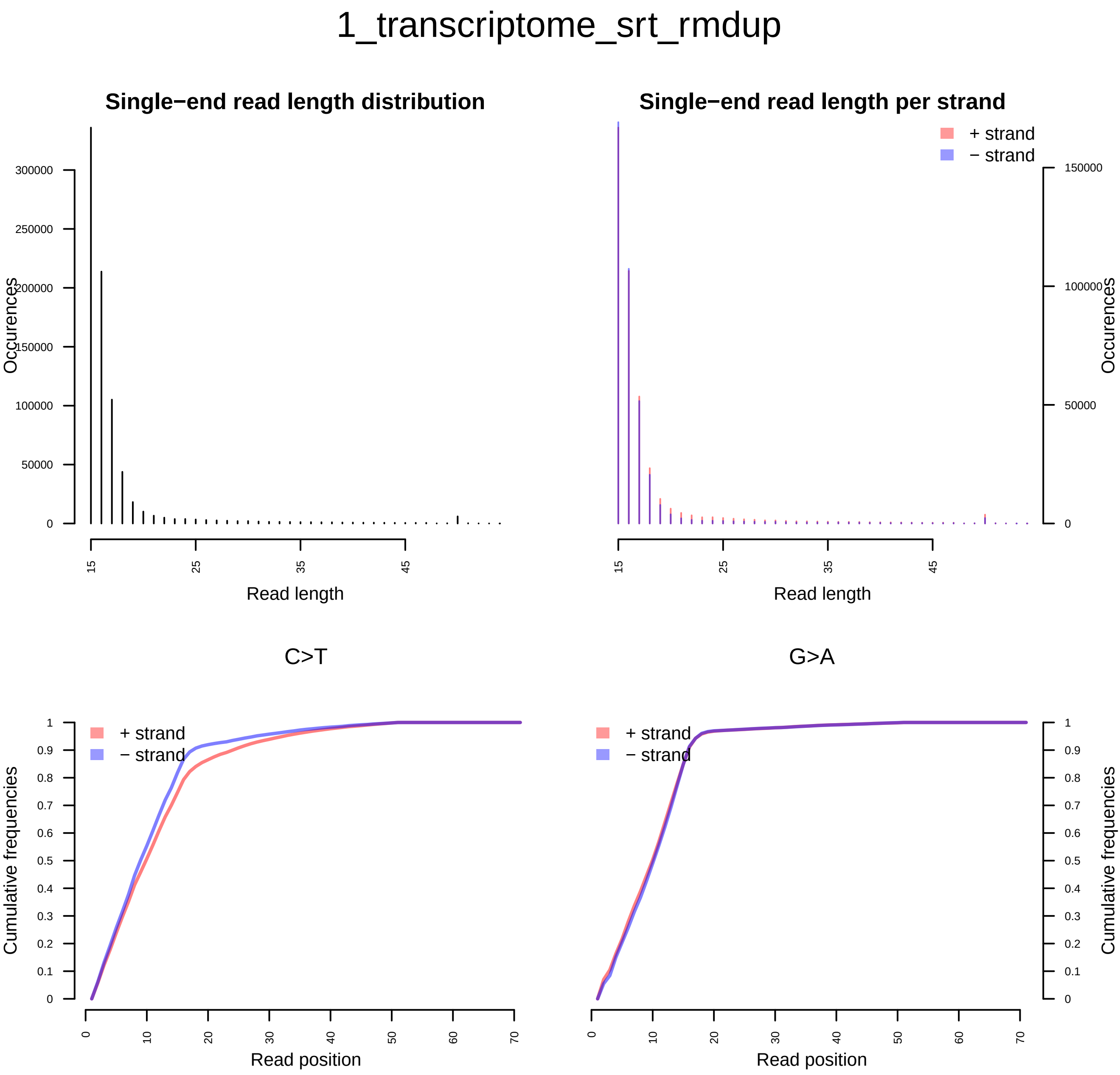
1_transcriptome_srt_rmdup
Single−end read length distribution
Occurences
0
50000
100000
150000
200000
250000
300000
15
25
35
45
Read length
Single−end read length per strand
+ strand
− strand
Occurences
0
50000
100000
150000
15
25
35
45
Read length
C>T
+ strand
− strand
Cumulative frequencies
0
0.1
0.2
0.3
0.4
0.5
0.6
0.7
0.8
0.9
1
0
10
20
30
40
50
60
70
Read position
G>A
+ strand
− strand
Cumulative frequencies
0
0.1
0.2
0.3
0.4
0.5
0.6
0.7
0.8
0.9
1
0
10
20
30
40
50
60
70
Read position
