# Supplementary material for: Ancient RNA from Late Pleistocene permafrost and historical canids shows tissue-specific transcriptome survival
Source: PLoS Biol. 2019 Jul 30;17(7):e3000166. doi: 10.1371/journal.pbio.3000166 (PMC6667121; doi:10.1371/journal.pbio.3000166)
Supplement: S1 Data — (ZIP) [file pbio.3000166.s029.zip › S4_Fig.zip!/S4_Fig/results_Tumat_liver_trans_rmdup_30up/Fragmisincorporation_plot.pdf]

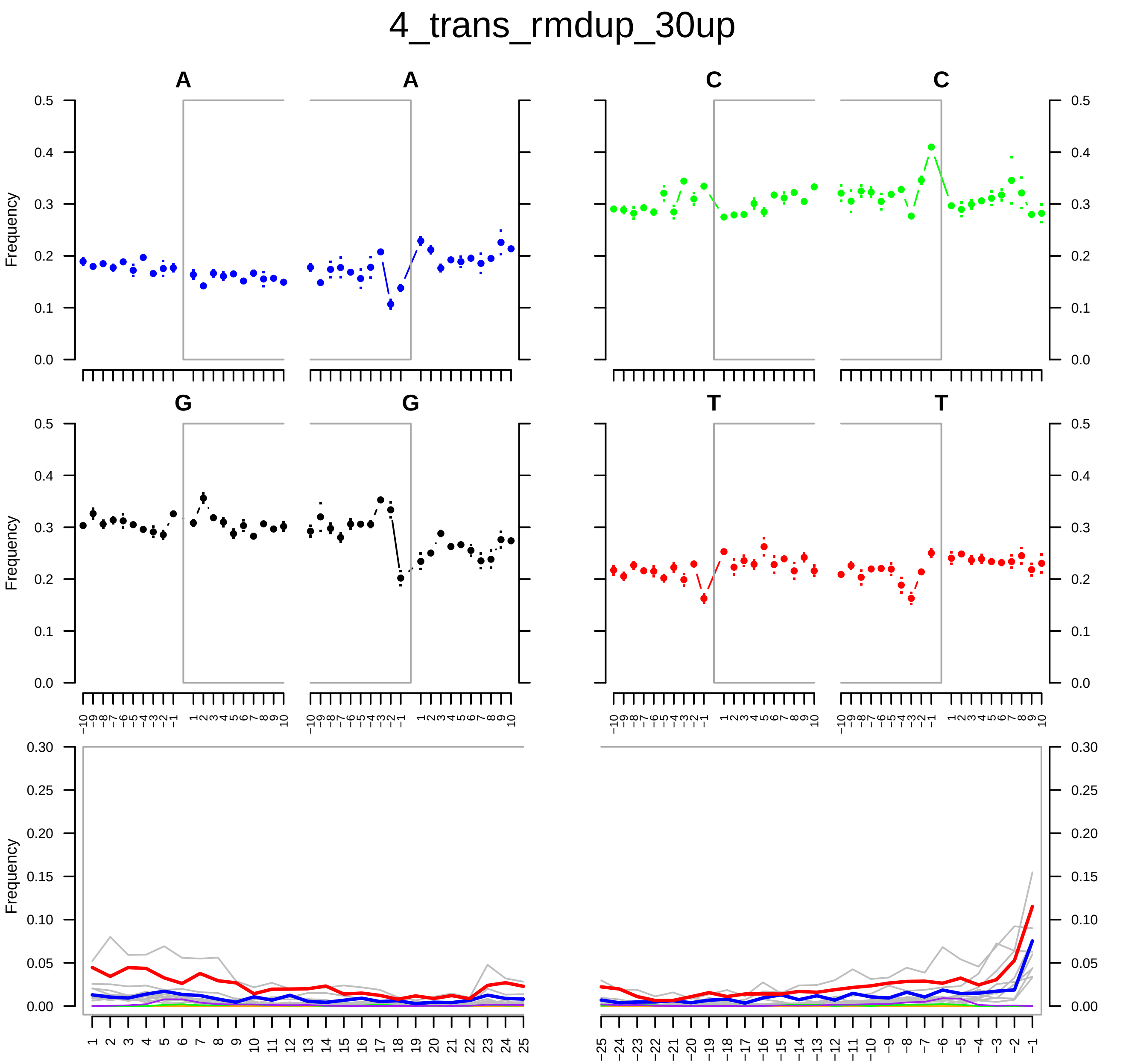

4_trans_rmdup_30up
A
A
C
C
G
G
T
T
Frequency
