# Supplementary material for: Ancient RNA from Late Pleistocene permafrost and historical canids shows tissue-specific transcriptome survival
Source: PLoS Biol. 2019 Jul 30;17(7):e3000166. doi: 10.1371/journal.pbio.3000166 (PMC6667121; doi:10.1371/journal.pbio.3000166)
Supplement: S1 Data — (ZIP) [file pbio.3000166.s029.zip › S5_Fig.zip!/S5_Fig/results_Tumat_Liver_trans_rmdup_30up/Length_plot.pdf]

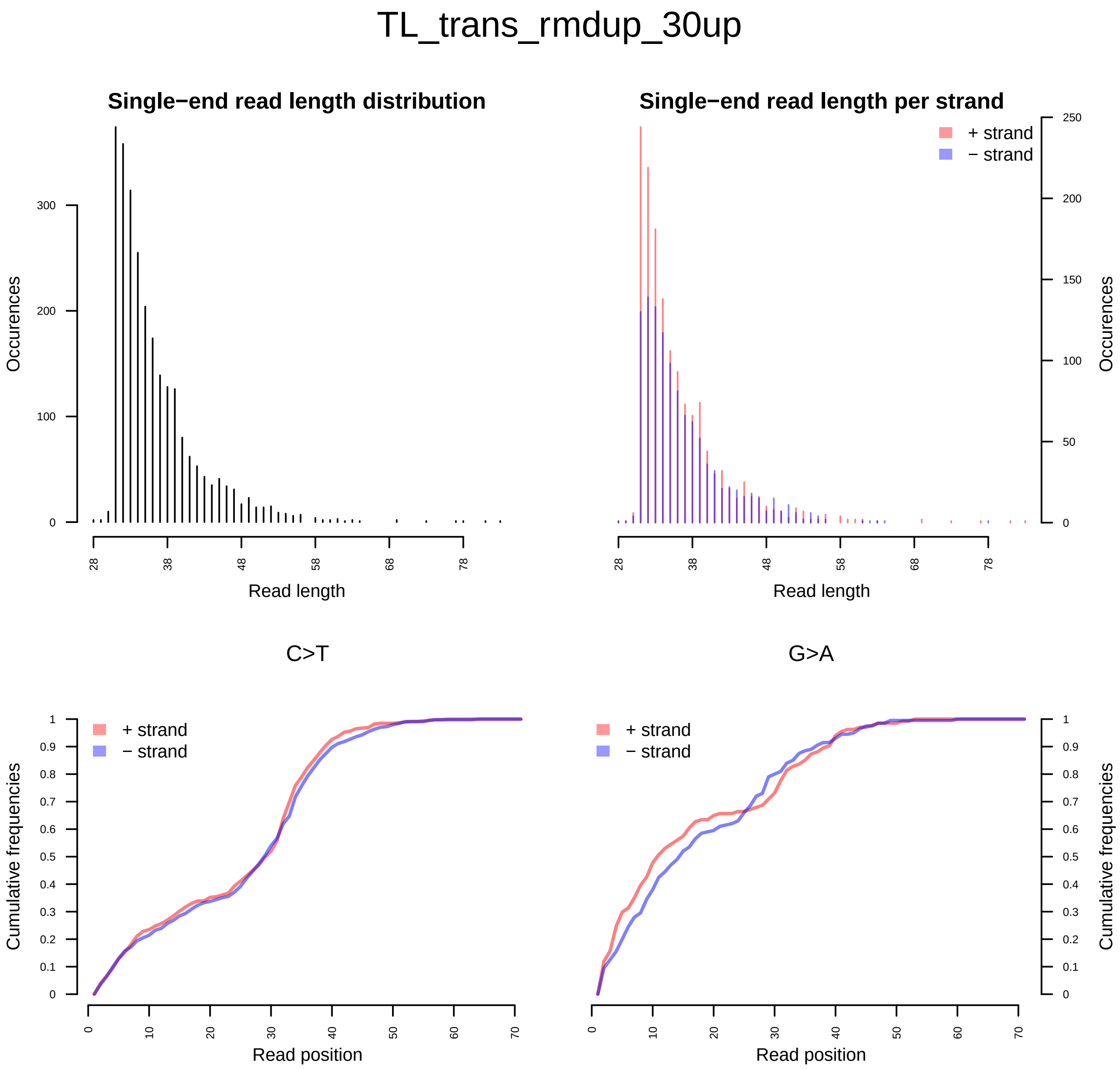
TL_trans_rmdup_30up
Single−end read length distribution
Occurences
Read length
Single−end read length per strand
+ strand
− strand
Occurences
Read length
C>T
+ strand
− strand
Cumulative frequencies
Read position
G>A
+ strand
− strand
Cumulative frequencies
Read position
