# Supplementary material for: Ancient RNA from Late Pleistocene permafrost and historical canids shows tissue-specific transcriptome survival
Source: PLoS Biol. 2019 Jul 30;17(7):e3000166. doi: 10.1371/journal.pbio.3000166 (PMC6667121; doi:10.1371/journal.pbio.3000166)
Supplement: S1 Data — (ZIP) [file pbio.3000166.s029.zip › S5_Fig.zip!/S5_Fig/results_Skin1_trans_rmdup_30up/Fragmisincorporation_plot.pdf]

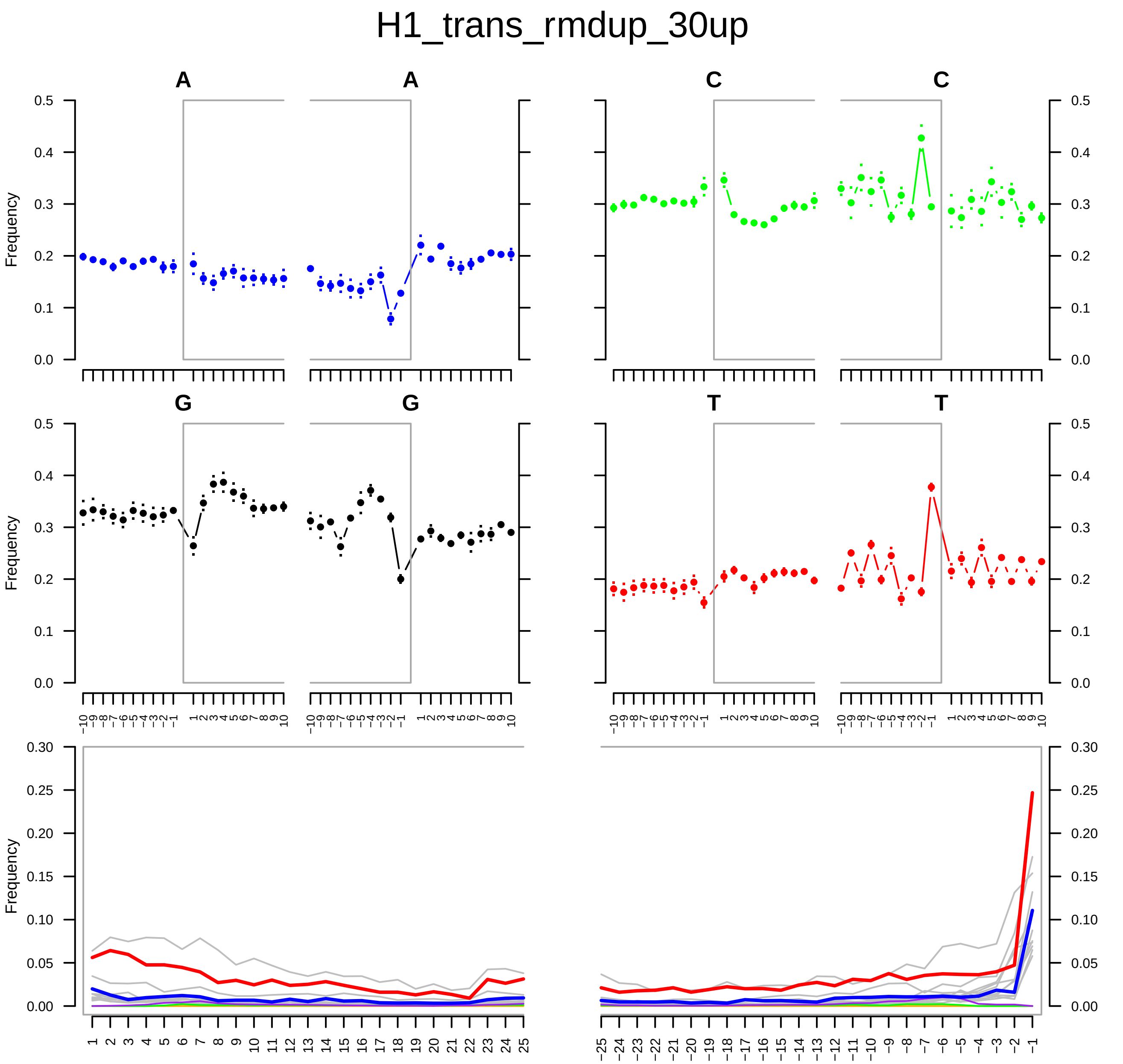

H1_trans_rmdup_30up
A
A
C
C
G
G
T
T
Frequency
0.0
0.1
0.2
0.3
0.4
0.5
0.00
0.05
0.10
0.15
0.20
0.25
0.30
-10 -9 -8 -7 -6 -5 -4 -3 -2 -1 1 2 3 4 5 6 7 8 9 10
1 2 3 4 5 6 7 8 9 10 11 12 13 14 15 16 17 18 19 20 21 22 23 24 25
-25 -24 -23 -22 -21 -20 -19 -18 -17 -16 -15 -14 -13 -12 -11 -10 -9 -8 -7 -6 -5 -4 -3 -2 -1
